# The Assembly of a High-Efficiency Tris-benzotriazolate-Based Metal–Organic Framework Solid-State Electrolyte

**DOI:** 10.1021/acscentsci.5c00567

**Published:** 2025-06-27

**Authors:** Zhangyi Xiong, Shitao Wu, Liang Gu, Mengyang Zhai, Yuke Pan, Yanhang Ma, Zhijie Chen

**Affiliations:** † Stoddart Institute of Molecular Science, Department of Chemistry, Zhejiang Key Laboratory of Excited-State Energy Conversion and Energy Storage, State Key Laboratory of Silicon and Advanced Semiconductor Materials, 12377 Zhejiang University, Hangzhou 310058, P. R. China; ‡ Zhejiang-Israel Joint Laboratory of Self-Assembling Functional Materials, ZJU-Hangzhou Global Scientific and Technological Innovation Center, 12377 Zhejiang University, Hangzhou 311215, P. R. China; § School of Physical Science and Technology & Shanghai Key Laboratory of High-resolution Electron Microscopy, 387433 ShanghaiTech University, Shanghai 201210, China

## Abstract

Metal–organic frameworks (MOFs) with tunable ion transport pathways are considered promising solid-state electrolyte (SSE) candidates for developing lithium or sodium metal batteries. However, their low ionic conductivity and inferior stability with metal anodes limit practical applications. Herein we synthesized a high-stability tris-benzotriazolate-based MOF—Cu-TTBT—with ordered pore channels for SSE applications via a network-directed approach. Cu-TTBT, overcoming the synthetic challenge of tritopic benzotriazolate-based linkers, greatly advances the field of azolate-based MOFs. The resultant framework displays fast ion transport pathways with a high ionic conductivity of 1.83 × 10^–4^ S cm^–1^ and 1.1 × 10^–4^ S cm^–1^ at 298 K for Cu-TTBT-Li and Cu-TTBT-Na, respectively, among the highest in azolate-based MOFs. The Li|SSE|LiFePO_4_ and Na|SSE|Na_3_V_2_(PO_4_)_3_ coin cells exhibit stable cycling performances over 200 cycles at 1.0 C and 298 K. This research advances the synthetic chemistry of azolate-based MOFs and paves the way for the development of robust frameworks with high-efficiency SSE performances.

## Graphical abstract

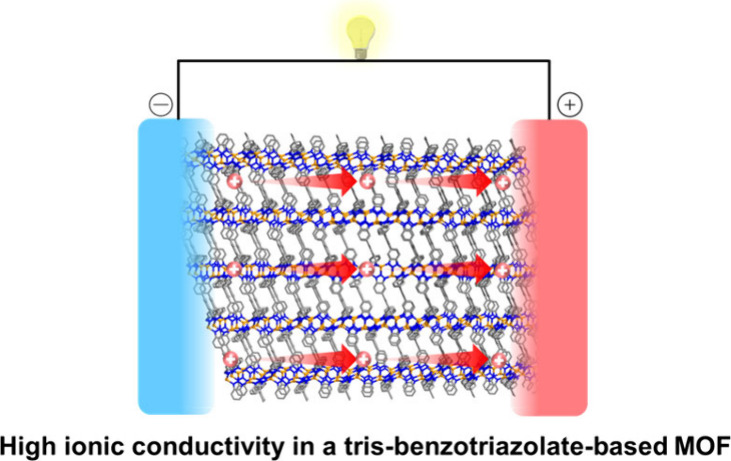

## Introduction

The lithium metal batteries (LMBs) with high theoretical energy density are promising next-generation energy storage candidates alternative to commercial lithium-ion batteries.^1^ However, the safety concerns of LMBs based on flammable organic electrolytes including Li dendrite growth and unstable electrolyte interphase layer have limited their practical implementation.^2^ The replacement of organic electrolytes with solid-state electrolytes (SSEs) is among the most compelling strategies to realize LMBs with synergistic high energy density and safety.^3^ The inorganic SSEs display high ionic conductivities, yet limited stability against air components and lithium metal anodes, limiting the performance of solid-state batteries. On the other hand, the polymeric SSEs exhibit outstanding interfacial compatibility and high stability, but the low ionic conductivity and Li^+^ transference number restrict their large-scale applications.^4–7^ Moreover, solid-state sodium metal batteries (SMBs) exhibit huge potential prospect in large-scale energy storage systems owing to their low-cost, safety and high energy density.^8^ To this end, it is highly demanding to develop high-performance Li-ion and Na-ion SSEs with high ionic conductivity and desirable stability toward the real-world applications of LMBs and SMBs.^4^

Metal–organic frameworks (MOFs), assembled from metal-containing nodes and organic ligands, are crystalline porous materials with high porosity, designability and well-defined pore structures.^9–15^ These intrinsic features enable MOFs with tunable ion transport pathways to be promising SSE platforms.^16–18^ In particular, anionic MOFs with charge-balancing cations serve as single-ion conductors to enhance Li^+^ or Na^+^ transference number.^17,19,20^ For example, MIT-20,^21^ a benzotriazolate-based anionic MOF with strong coordination bonds based on the Pearson’s hard/soft acid/base (HSAB) principle,^22^ displays high ionic conductivities of 4.4 × 10^–5^ S cm^–1^, 1.8 × 10^–5^ S cm^–1^, and 8.8 × 10^–7^ S cm^–1^ for Li^+^, Na^+^, and Mg^2+^ at 298 K, respectively. Other benzotriazolate-based MOFs also show exceptional SSEs performances owing to high stability and tunable coordination environments.^23–27^ Unfortunately, the current benzotriazolate-based MOFs are limited to ditopic linker systems,^27–30^ significantly limiting tunable ion transport pathways, potentially owing to the synthetic challenges of organic ligands and the further difficult crystallization between ligands and transition metal ions. It is expected that the increase of linker connectivity will significantly boost the synthesis of stable MOFs with diverse pore structures and tunable ion passways. However, it remains a challenge to synthesize such an MOF based on multitopic benzotriazolate-based ligands due to the challenging synthetic strategy for such linkers.

On our quest to develop a multitopic benzotriazolate linker, we synthesized a tritopic tris-benzotriazole -5,5′,5″-(2,4,6-trimethylbenzene-1,3,5-triyl)­tris­(1H-benzo­[d]­[1,2,3]­triazole (H_3_TTBT) via Suzuki coupling reaction as a proof-of-concept. This newly developed strategy paves a way for the development of multitopic benzotriazolate-based ligands on account of the well-established Suzuki coupling strategy.^31,32^ Different from previous benzotriazolate-based MOFs, the network-directed assembly of 3-connected TTBT linkers with copper ions under the optimized conditions allows the synthesis of azolate-based Cu-TTBT microcrystals, and three-dimensional electron diffraction (3D ED) data permit the atomic-level crystal structural determination. The single crystal structural analysis revealed that Cu-TTBT consisted of infinite copper-containing rod-packing building units formed by copper ions coordinated with tris-benzotriazolate linkers. The ion-exchanged Cu-TTBT exhibits efficient Li-ion and Na-ion transport pathways with high ionic conductivities of 1.83× 10^–4^ S cm^–1^ and 1.1× 10^–4^ S cm^–1^ at 298 K, respectively. The Li|SSE|LiFePO_4_ and Na|SSE|Na_3_V_2_(PO_4_)_3_ coin cells using Cu-TTBT as an SSE exhibit stable cycling performances over 200 cycles at 1.0 C and 298 K. This work, providing a viable strategy for the design of a next-generation of azolate-based MOFs with high stability, diverse pore structures, and high ionic conductivity of these frameworks, allows them to be promising SSEs of LMBs and SMBs.

## Results and Discussion

### Synthesis and Structural Analysis

Currently, the reported benzotriazolate-based MOFs are based on ditopic ligands, including MFU-4, MFU-4*l*, MAF-X27, CFA-1, V_2_Cl_2.8_(btdd), MIT-20, NU-2100 (Table S6).^21,28,29,33–35^ Inspired by carboxylate-based MOF chemistry, the increase of linker connectivity will boost the synthesis of structures with good stability, diverse pore sizes, and directed functionalities. As a proof-of-concept, we designed and synthesized a 3-connected tris-benzotriazole ligand (H_3_TTBT) via Suzuki coupling reaction,^32^ Boc-deprotection reaction and further diazotization and cyclization reactions^33,36^ (Figure 1a). We believe this network-directed approach presents a viable method for the development of multitopic benzotriazolate-based ligands. The purity of H_3_TTBT is confirmed by nuclear magnetic resonance (NMR) spectroscopy and mass spectrum (Figures S1–S6). Reactions between CuCl_2_·2H_2_O and the tris-benzotriazole ligand in the presence of trifluoroacetic acid in an *N*, *N*-dimethylformamide solution yield crystalline Cu-TTBT powders. The large-size single crystal of Cu-TTBT is difficult to obtain due to the poor reversibility of strong coordination bonds formed in this robust MOF.

**Figure 1:**
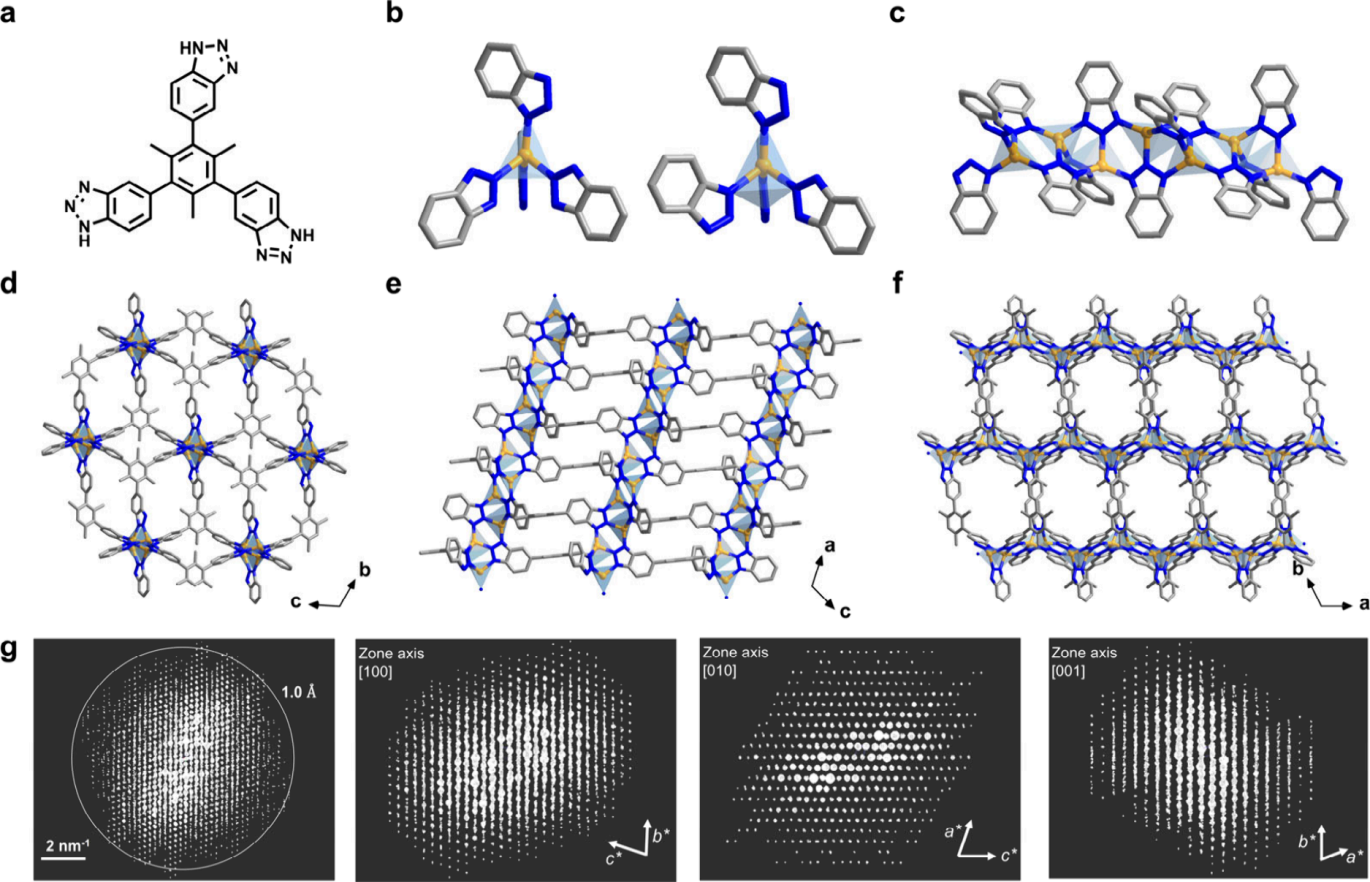
(a) Tris-benzotriazolate ligand (H_3_TTBT) for the assembly of Cu-TTBT. (b) Two copper-based nodes with different coordination environments. (c) Infinite copper-containing rod-like secondary building units. (d-f) Structures of Cu-TTBT at different directions. (g) Projections of 3D ED data for Cu-TTBT along different directions. H atoms were omitted for clarity; color code for structure: Cu, yellow; C, gray; N, blue.

The powder X-ray diffraction (PXRD) pattern of Cu-TTBT (Figure S11) shows sharp and intense reflection peaks, suggesting a high crystallinity of the sample. The scanning electron microscopy (SEM) images of Cu-TTBT reveal the morphology and size of the particles (Figure S12). The single crystal structure of Cu-TTBT was solved using 3D ED.^37^ 3D ED data with a resolution up to 1.0 Å were collected from single nanocrystals. A triclinic unit cell was determined from 3D ED data: *a* = 11.24 Å, *b* = 15.16 Å, *c* = 15.49 Å, α = 106.91°, β = 109.69°, γ = 107.40°, and *V* = 2133 (9) Å^3^ (Table S1). *Ab initio* structure solution was obtained using the space group of *P*1̅. It turns out that Cu-TTBT framework consists of one-dimensional infinite rod-like secondary building units (SBU) constructed from two tetrahedral copper clusters of different coordination environments and benzotriazolate linkers (Figures 1b-f).^38–40^ The calculated PXRD pattern using the solved crystal structure matches well with the experimental one (Figure S13), indicating the pure phase of Cu-TTBT. Fourier transform infrared (FTIR) spectroscopy revealed that the ligands may remain partially protonated in Cu-TTBT (Figure S14).^41^ X-ray photoelectron spectroscopy (XPS) illustrated the existence of copper with mixed valence states, 70% Cu (I) species and 30% Cu (II) species (Figure S15).^28^ Combining 3D ED with XPS and FTIR results, the formula could be defined as Cu_2_H_0.4_TTBT.

Topological analysis revealed that Cu-TTBT, constructed from 3-connected linkers and infinite rod-packing SBUs, is based on a rare underlying (3,3,5,7)-connected net, which is different from the 5-connected network of MIT-20 (Figure 2 and Figures S19–S21). This (3,3,5,7)-connected net has a compact point symbol expressed as {3.8^2^}_3_{3^5^.4.8^2^.9^2^}­{3^8^.4^5^.5^2^.8^3^.9^3^}_2_. Overall, the incorporation of a 3-connected tris-benzotriazolate linker into the azolate-based MOF system affords a new type of crystalline framework with unique pore channels that is amenable to diverse applications.

**Figure 2:**
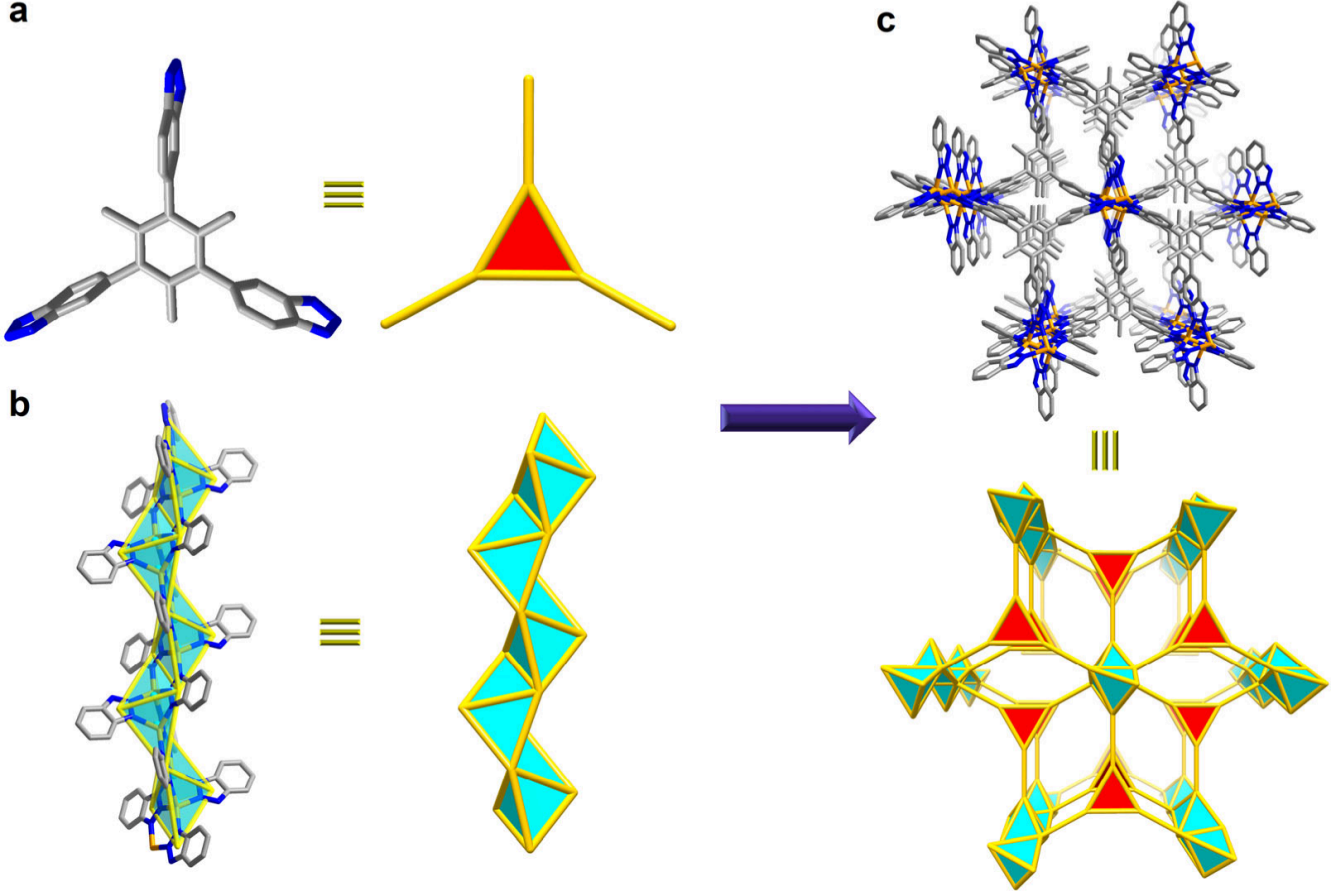
(a) Triangular core of the TTBT linker. (b) Infinite rod-like secondary building units. (c) The crystal structure and topological network of Cu-TTBT.

The porosity of Cu-TTBT was examined by N_2_ adsorption–desorption measurement at 77 K. Cu-TTBT exhibits an apparent Brunauer–Emmett–Teller (BET) surface area of 1140 m^2^ g^–1^ satisfying all four consistency criteria (Figures S16 and S17) and an experimental total pore volume of 0.47 cm^3^ g^–1^ at *P*/*P*_0_ = 0.90. The pore size distribution from a density functional theory model with cylinder pore geometry revealed one type of pore centered at 6.3 Å, which is consistent with the determined structure (Figure S10). Benefiting from multiconnected Cu–N coordinative bonds, Cu-TTBT exhibits high thermal and chemical stability. The high thermal stability of Cu-TTBT was supported by high N_2_ adsorption capacity at 77 K and PXRD patterns at activated temperature up to 180 °C (Figures 3a and 3b). Additionally, the PXRD patterns and N_2_ sorption isotherms of Cu-TTBT were maintained well after being soaked in the aqueous solutions (pH = 3 and pH = 12) for 24 h (Figures 3c and 3d), indicating the high chemical stability. Thermogravimetric analysis of Cu-TTBT displays a mass loss occurring at 280 °C, at which point the materials degrade (Figure S18).

**Figure 3:**
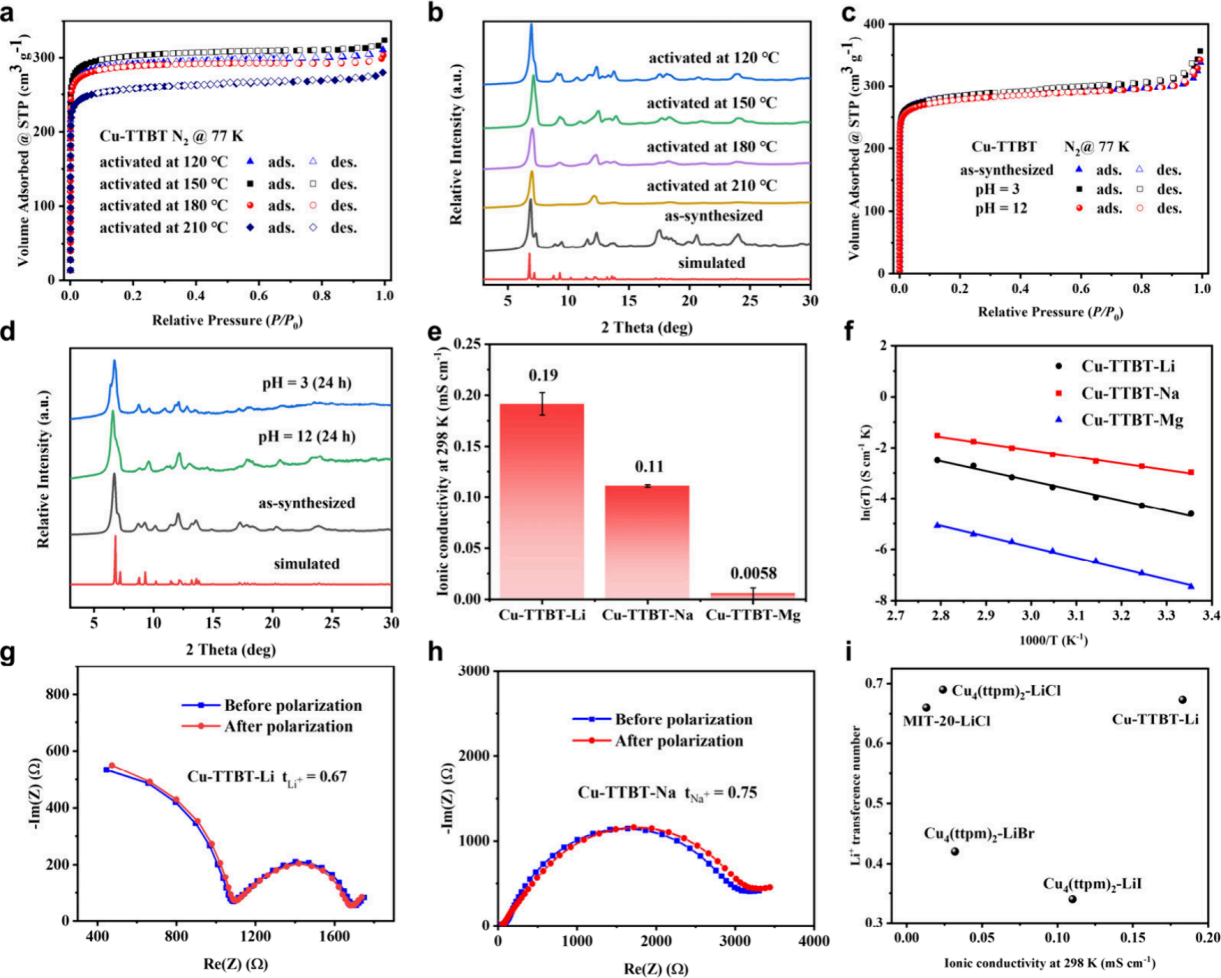
(a-d) N_2_ sorption isotherms at 77 K and PXRD patterns of Cu-TTBT. (e-f) Ionic conductivities and temperature-dependent conductivity plots of Cu-TTBT-X (X = Li, Na, and Mg). (g) *t*_Li+_ of Cu-TTBT-Li. (h) *t*_Na+_ of Cu-TTBT-Na. (i) Comparison of the ionic conductivities of azolate-based MOFs.

### Ionic Conductivity Performance

On account of its stability and ion exchange capacity, Cu-TTBT can serve as a promising platform for high-efficiency SSEs applications. Soaking Cu-TTBT powder in tetrahydrofuran solutions of various metal salts (i.e., LiCl, NaSCN, and MgBr_2_) results in ion-exchanged MOFs by substituting the remaining proton with metal cations.^42^ The obtained MOFs were further washed with propylene carbonate (PC) to solvate the metal cations and improve interparticle conductivity.^43^ PXRD patterns of Cu-TTBT-X (X = Li, Na, and Mg) showed similar diffraction peaks, consistent with the exchange of different ions having the same structure (Figure S22). The structures of Cu-TTBT-Li and Cu-TTBT-Na resolved by 3D ED experiment are similar to that of Cu-TTBT with a slight expansion of the unit cell (Table S1). The ionic conductivities of Cu-TTBT-Li, Cu-TTBT-Na, and Cu-TTBT-Mg at 298 K are 1.83 × 10^–4^ S cm^–1^, 1.1 × 10^–4^ S cm^–1^, 1.92 × 10^–6^ S cm^–1^, respectively (Figures 3e, Figures S23–S25, and Table S2). The activation energy (*E*_a_) was fitted based on alternating current impedance data of various temperature from 25 to 85 °C and the Arrhenius equation (Figure 3f and Figures S26–S28). The *E*_a_ of Cu-TTBT-Li, Cu-TTBT-Na, and Cu-TTBT-Mg were tested as 0.30, 0.22, and 0.36 eV, respectively. On account of their good SSE performances, we further focus on the detailed study of Cu-TTBT-Li and Cu-TTBT-Na. The apparent BET areas and pore volumes of Cu-TTBT-Li and Cu-TTBT-Na are significantly lower than those of the parent materials, suggesting Li-ions or Na-ions can occupy the pore space (Figure S29). The energy-dispersive X-ray spectroscopy (EDX) mapping of Cu-TTBT-Li and Cu-TTBT-Na displays the corresponding element distribution, respectively (Figures S30 and S31). The Cu valence state of Cu-TTBT-Li and Cu-TTBT-Na was investigated by XPS, which exhibit a similar rate of Cu (I) and Cu (II) (Figure S32 and Table S3). The corresponding Li and Na elements can be detected for Cu-TTBT-Li and Cu-TTBT-Na (Figure S33). The inductively coupled plasma mass-spectrometry analysis of Cu-TTBT-Li and Cu-TTBT-Na confirmed a Li: Cu ratio of 1:9 and a Na: Cu ratio of 1.5:8.5, respectively (Tables S4 and S5). The content of PC was determined by ^1^H NMR spectroscopy of digested Cu-TTBT-Li and Cu-TTBT-Na (Figures S34 and S35). The final electrolyte formula was estimated to be Li_0.23_Cu_2_H_0.17_TTBT·3PC for Cu-TTBT-Li and Na_0.35_Cu_2_H_0.05_TTBT·5.5PC for Cu-TTBT-Na.

The transfer numbers of Cu-TTBT-Li and Cu-TTBT-Na were calculated to evaluate the contribution of Li^+^ and Na^+^ to the ionic conductivity. The transference number was *t*_Li+_ = 0.67 for Cu-TTBT-Li and *t*_Na+_ = 0.75 for Cu-TTBT-Na, comparable to the values of the reported benzotriazolate-based MOF, ionic COFs, and ionic MOFs solid electrolytes (Figures 3g–i, Figures S36–S37 and Tables S7–S8). The high ionic diffusion kinetics of Cu-TTBT-Li and Cu-TTBT-Na was further investigated by combining solid-state nuclear magnetic resonance (SSNMR) spectroscopy and molecular dynamics (MD) simulations. The saturation recovery spectra of Cu-TTBT-Li and Cu-TTBT-Na present the spin–lattice relaxation time of 71.55 and 7.03 ms, respectively (Figure S38). The low spin–lattice relaxation time can be associated with high ionic mobility for Cu-TTBT-Li and Cu-TTBT-Na. Furthermore, the temperature-dependent ^7^Li SSNMR and ^23^Na SSNMR spectra in a temperature range from 270 to 340 K were performed to monitor ion diffusion dynamics of Cu-TTBT-Li and Cu-TTBT-Na, respectively. Both ^7^Li SSNMR spectra of Cu-TTBT-Li and ^23^Na SSNMR spectra of Cu-TTBT-Na display the uniform line shape resonance for all temperatures, implying the high Li^+^ and Na^+^ ions migration in Cu-TTBT-Li and Cu-TTBT-Na, respectively (Figure S39).^44^ The full widths at half-maximum gradually shrink with the increase of temperature due to the narrowing effect of the ^7^Li–^7^Li and ^23^Na–^23^Na (Figure S40). 2D exchange SSNMR spectra was further conducted to study the chemical environments of spontaneous Li^+^ and Na^+^ exchange during a certain mixing time (*t*_mix_). ^7^Li–^7^Li and ^23^Na–^23^Na exchange spectra at different *t*_mix_ maintain equivalent intensity (Figures S41 and S42), suggesting the spontaneous and continuous ion conduction in only one chemical environment and further revealing the nature of single ion conductors.^45^ The ion diffusion kinetics are further analyzed by molecular dynamics (MD) simulations. Based on the mean-square displacements simulation, the diffusion coefficients of Cu-TTBT-Li and Cu-TTBT-Na were estimated as 5.67 × 10^–7^ cm^2^ s^–1^ and 2.17 × 10^–7^ cm^2^ s^–1^, respectively (Figures S43 and S44). These computational simulations and experimental results support the high ion transfer kinetics and high ionic conductivity of Cu-TTBT.

The cross-sectional EDX mapping images of Cu-TTBT-Li and Cu-TTBT-Na exhibit the presence of C, N, and Cu elements, respectively (Figures S45 and S46). The Cu-TTBT-Li and Cu-TTBT-Na display good electrochemical stabilities with a voltage window of 0–4.4 V and 0–4.6 V, respectively (Figures S47 and S48). The long-term Li and Na plating/stripping cycle stabilities of Cu-TTBT-Li and Cu-TTBT-Na were investigated by operating Li|SSE|Li and Na|SSE|Na symmetrical cells, respectively. The symmetrical cells with Cu-TTBT-Li and Cu-TTBT-Na can operate for at least 300 h without short circuit (Figures S49 and S50). The PXRD patterns of Cu-TTBT-Li and Cu-TTBT-Na after plating/stripping cycles show diffraction peaks similar to those of pristine Cu-TTBT-Li and Cu-TTBT-Na (Figure S51). N_2_ adsorption–desorption isotherms at 77 K illustrate that Cu-TTBT-Li and Cu-TTBT-Na after plating/stripping cycles show lower adsorption capacity than the parent materials owing to the pore space filled with metal ions and propylene carbonate molecules after plating/stripping cycles (Figure S52). The SEM images reveal that Cu-TTBT-Li and Cu-TTBT-Na after plating/stripping cycles exhibit the smooth and compacting surface, suggesting good stability with Li and Na metal (Figures S53 and S54). In addition, the issues of Li dendrites and Na dendrites on the surface of Li and Na electrode are investigated, respectively. The Li and Na electrode after plating/stripping cycles present a smooth surface (Figures S55 and S56), indicating uniform deposition behavior for Li|SSE|Li and Na|SSE|Na symmetrical cells.^46^ These results suggest Cu-TTBT-Li and Cu-TTBT-Na can be employed as SSEs for lithium and sodium metal battery.

### Electrochemical Performance of Assembled Battery

A quasi-solid-state LMB with LiFePO_4_ cathode, Cu-TTBT solid electrolyte and lithium anode were assembled.^47^ The SSE performance was investigated by long-term cycling measured at ambient temperature and a voltage window between 2.8 and 3.8 V with a current density of 1.0 C. The initial discharge capacity is 105 mAh g^–1^. After 200 cycles, the capacity slowly decreases to 101 mAh g^–1^, with a retention of 96% and a capacity loss of 0.03 mAh g^–1^ per cycle (Figures 4d and 4e). The rate performances are 150, 125, 96, and 44 mAh g^–1^ at 0.2, 0.5, 1.0, and 2.0 C, respectively. After cycling at 2.0 C, the capacity can be recovered, presenting an excellent rate performance (Figure 4f). The depth-resolved XPS experiments with different etching time were conducted to investigate the compositional evolution of the solid electrolyte interphase (SEI) formation at the electrolyte/Li anode interface. The C 1s spectral analysis reveals that the intensity of O–C=O peak increased and maintained stability as the sputtering time increases, mainly attributed to the decomposition of PC solvent (Figure S57).^48^ This evolution is also presented in the O 1s spectra, which exhibit parallel evolution of Li_2_O species. Concurrently, the F 1s and S 2p spectra demonstrate a systematic enhancement of LiF and -SO_x_ species with the increased sputtering depths, respectively (Figure S58). Collectively, these depth-resolved XPS experiments confirm the presence of SEI layer comprised of inorganic components (LiF, Li_2_CO_3_, LiOH, Li_2_SO_4_) and organic derivatives.^48^ We further used the depth-resolved time-of-flight secondary ion mass spectrometry to study the spatiotemporal evolution of the SEI layer.^49^ The Li^–^ fragment intensity exhibited a progressive increase throughout the sputtering period, suggesting a limited initial lithium concentration at the electrode–electrolyte interface. In contrast, the characteristic signals for C^–^, F^–^, and CH^–^ fragments demonstrated distinct transient behavior, reaching maximum intensities at approximately 30 s before gradually attenuating with the sputtering time increasing (Figure S59). This temporal decoupling between inorganic (F^–^) and organic (C^–^, CH^–^) species indicates a stratified SEI architecture, where near-surface regions are dominated by metastable organic decomposition products, while inorganic lithium-rich compounds preferentially accumulate in deeper interfacial layers. These results collectively reveal the formation of SEI layer with enhanced interfacial stability and uniform passivation characteristics on the lithium metal substrate.^49^

**Figure 4:**
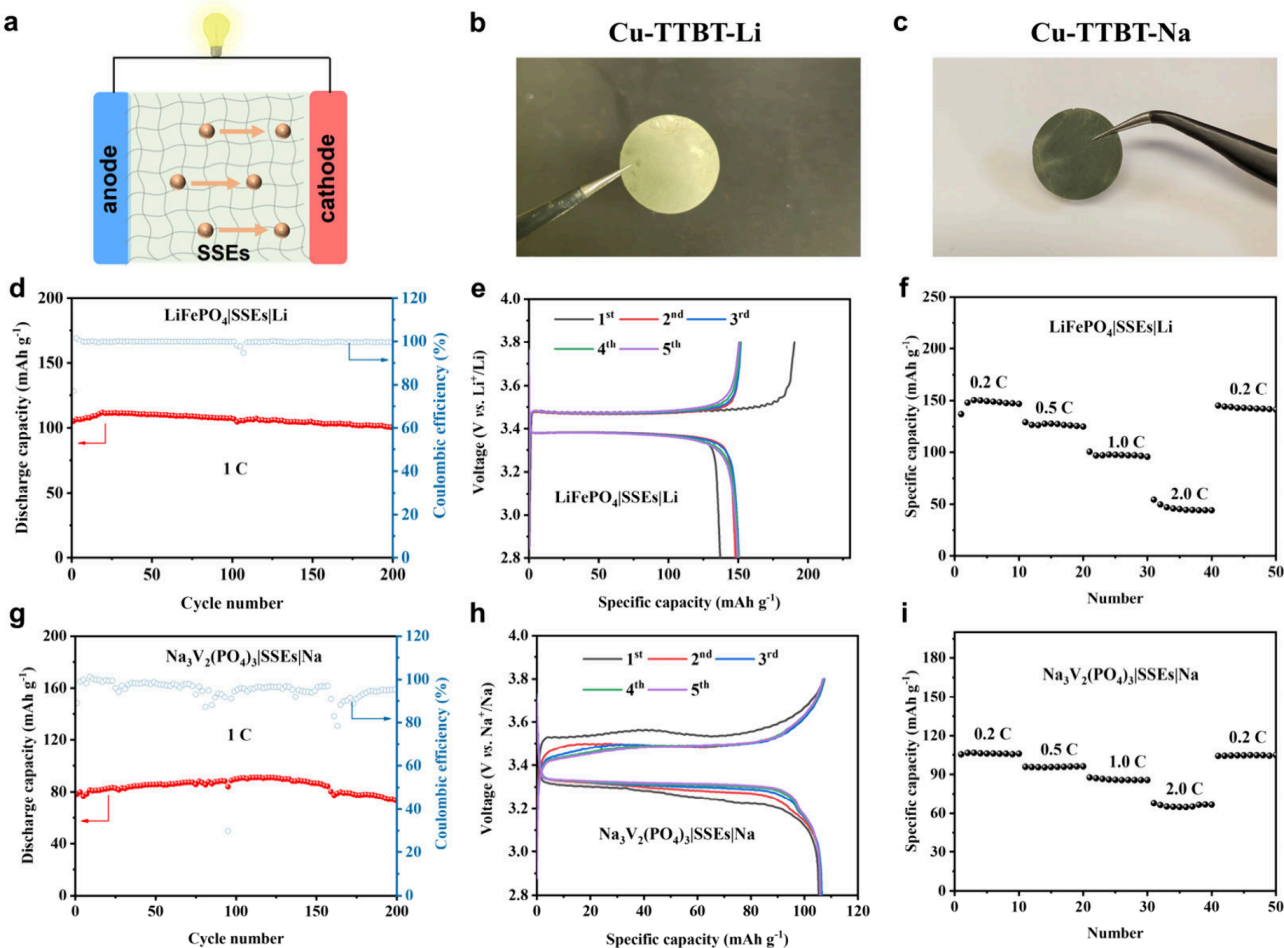
(a) Illustration of lithium metal battery and sodium metal battery assembly structure. (b, c) Optical images of SSE films based on Cu-TTBT-Li and Cu-TTBT-Na. (d, g) Cycling performance of the LMB and SMB. (e, h) Charge–discharge voltage profiles of the LMB and SMB at different cycles. (f, i) Rate performance of the LMB and SMB at different current densities.

Beyond LMBs, SMBs are promising candidates for large-scale energy storage systems due to low costs and abundant sodium sources. The quasi-solid-state SMB with Na_3_V_2_(PO_4_)_3_ cathode, Cu-TTBT-Na solid electrolyte, and sodium anode were assembled to evaluate the Na ion conductivity performance. The cycling performance of SMB was also confirmed by long cycling at 1.0 C between 2.8 and 3.8 V. SMB delivers a stable capacity retention of 94% after 200 cycles, which reveals the electrochemical stability of Cu-TTBT-Na toward the sodium metal anode (Figures 4g and 4h). The rate performances are 164, 105, 96, 88, and 68 mAh g^–1^ at 0.2, 0.5, 1.0, and 2.0 C, respectively. After cycling at 2.0 C, the capacity was recovered (Figure 4i).

## Conclusions

In summary, we have applied a network-directed strategy to rationally synthesize a 3-connected tris-benzotriazolate, and the further assembly of this linker with copper ions yields a next-generation robust MOF based on multitopic benzotriazolate linkers. The resultant high-stability Cu-TTBT displays fast Li-ion and Na-ion conductivities, suggesting a promising SSE for LMBs and SMBs. Our results demonstrate the validity of the high-connectivity benzotriazolate strategy to expand the boundaries of azolate-based MOF crystal chemistry. Finally, we envision that our strategy introduces a synthetic route to MOF-based SSEs with both concurrently high ionic conductivities and high stability for lithium and sodium metal batteries via regulation of linker connectivity and pore structures.

## Supplementary Material









## Notes

Additional descriptions of general methods and materials, synthetic procedures, powder X-ray diffraction analysis, scanning electron microscopy, transmission electron microscopy, crystallography, N_2_ sorption measurements, thermogravimetric analysis, inductively coupled plasma-mass spectrometry, time-of-flight secondary ion mass spectrometry, solid-state nuclear magnetic resonance, X-ray photoelectron spectroscopy, molecular dynamic simulations, and electrochemical performance data (PDF)
X-ray data for Cu-TTBT (CIF)
X-ray data for Cu-TTBT-Li (CIF)
X-ray data for Cu-TTBT-Na (CIF)

## References

[1] Yu P., Zhang H., Hussain F., Luo J., Tang W., Lei J., Gao L., Butenko D., Wang C., Zhu J., Yin W., Zhang H., Han S., Zou R., Chen W., Zhao Y., Xia W., Sun X. (2024). Lithium metal-compatible
antifluorite electrolytes for solid-state batteries. J. Am. Chem. Soc..

[2] Lee M. J., Han J., Lee K., Lee Y. J., Kim B. G., Jung K.-N., Kim B. J., Lee S. W. (2022). Elastomeric electrolytes for high-energy
solid-state lithium batteries. Nature.

[3] Zhou L., Zuo T.-T., Kwok C. Y., Kim S. Y., Assoud A., Zhang Q., Janek J., Nazar L. F. (2022). High areal capacity,
long cycle life 4 V ceramic all-solid-state Li-ion batteries enabled
by chloride solid electrolytes. Nat. Energy.

[4] Chen R., Li Q., Yu X., Chen L., Li H. (2020). Approaching practically
accessible solid-state batteries: stability issues related to solid
electrolytes and interfaces. Chem. Rev..

[5] Manthiram A., Yu X., Wang S. (2017). Lithium battery chemistries enabled by solid-state
electrolytes. Nat. Rev. Mater..

[6] Zhao Q., Stalin S., Zhao C.-Z., Archer L. A. (2020). Designing solid-state
electrolytes for safe, energy-dense batteries. Nat. Rev. Mater..

[7] Chi X., Li M., Chen X., Xu J., Yin X., Li S., Jin Z., Luo Z., Wang X., Kong D., Han M., Xu J.-J., Liu Z., Mei D., Wang J., Henkelman G., Yu J. (2023). Enabling high-performance all-solid-state
batteries via guest wrench in zeolite strategy. J. Am. Chem. Soc..

[8] Zhang F., He B., Xin Y., Zhu T., Zhang Y., Wang S., Li W., Yang Y., Tian H. (2024). Emerging chemistry for wide-temperature
sodium-ion batteries. Chem. Rev..

[9] Shi L., Xiong Z., Wang H., Cao H., Chen Z. (2024). Quasicrystal
approximants in isoreticular metal-organic frameworks via Cairo pentagonal
tiling. Chem..

[10] Chen Z., Jiang H., Li M., O’Keeffe M., Eddaoudi M. (2020). Reticular chemistry 3.2: typical
minimal edge-transitive
derived and related nets for the design and synthesis of metal–organic
frameworks. Chem. Rev..

[11] Shi L., Yang Z., Sha F., Chen Z. (2023). Design, synthesis and
applications of functional zirconium-based metal-organic frameworks. Sci. China Chem..

[12] Hanikel N., Pei X., Chheda S., Lyu H., Jeong W., Sauer J., Gagliardi L., Yaghi O. M. (2021). Evolution of water structures in
metal-organic frameworks for improved atmospheric water harvesting. Science.

[13] Yuan S., Feng L., Wang K., Pang J., Bosch M., Lollar C., Sun Y., Qin J., Yang X., Zhang P., Wang Q., Zou L., Zhang Y., Zhang L., Fang Y., Li J., Zhou H. C. (2018). Stable
metal–organic frameworks: design, synthesis, and applications. Adv. Mater..

[14] Liang B., Zhang X., Xie Y., Lin R.-B., Krishna R., Cui H., Li Z., Shi Y., Wu H., Zhou W., Chen B. (2020). An ultramicroporous metal–organic framework for high sieving
separation of propylene from propane. J. Am.
Chem. Soc..

[15] Shi L., Zhong Y., Cao H., Wang H., Xiong Z., Wang K., Shen H., Chen Z. (2024). A hetero-supermolecular-building-block
strategy for the assembly of porous (3,12,24)-connected uru metal–organic
frameworks. Nat. Synth..

[16] Han Z., Zhang R., Jiang J., Chen Z., Ni Y., Xie W., Xu J., Zhou Z., Chen J., Cheng P., Shi W. (2023). High-efficiency
lithium-ion transport in a porous coordination chain-based
hydrogen-bonded framework. J. Am. Chem. Soc..

[17] Wiers B. M., Foo M.-L., Balsara N. P., Long J. R. (2011). A solid lithium
electrolyte via addition of lithium isopropoxide to a metal–organic
framework with open metal sites. J. Am. Chem.
Soc..

[18] Kim S., Jamal H., Khan F., Al-Ahmed A., Abdelnaby M. M., Al-Zahrani A., Chun S.-E., Kim J. H. (2024). Achieving high durability
in all-solid-state lithium metal batteries using metal–organic
framework solid polymer electrolytes. J. Mater.
Chem. A.

[19] Fischer S., Roeser J., Lin T. C., DeBlock R. H., Lau J., Dunn B. S., Hoffmann F., Fröba M., Thomas A., Tolbert S. H. (2018). A metal–organic framework
with tetrahedral aluminate sites as a single-ion Li^+^ solid
electrolyte. Angew. Chem., Int. Ed..

[20] Xu W., Pei X., Diercks C. S., Lyu H., Ji Z., Yaghi O. M. (2019). A metal–organic
framework of organic vertices and polyoxometalate linkers as a solid-state
electrolyte. J. Am. Chem. Soc..

[21] Park S. S., Tulchinsky Y., Dincă M. (2017). Single-ion Li^+^, Na^+^, and Mg^2+^ solid electrolytes supported by a mesoporous
anionic Cu–azolate metal–organic framework. J. Am. Chem. Soc..

[22] Chen Z., Kirlikovali K. O., Shi L., Farha O. K. (2023). Rational design
of stable functional metal–organic frameworks. Mater. Horiz..

[23] Chen Z., Mian M. R., Lee S.-J., Chen H., Zhang X., Kirlikovali K. O., Shulda S., Melix P., Rosen A. S., Parilla P. A., Gennett T., Snurr R. Q., Islamoglu T., Yildirim T., Farha O. K. (2021). Fine-tuning a robust metal–organic
framework toward enhanced clean energy gas storage. J. Am. Chem. Soc..

[24] Tulchinsky Y., Hendon C. H., Lomachenko K. A., Borfecchia E., Melot B. C., Hudson M. R., Tarver J. D., Korzyński M. D., Stubbs A. W., Kagan J. J., Lamberti C., Brown C. M., Dincă M. (2017). Reversible capture and release of
Cl2 and Br2 with
a redox-active metal–organic framework. J. Am. Chem. Soc..

[25] Oppenheim J. J., Mancuso J. L., Wright A. M., Rieth A. J., Hendon C. H., Dincǎ M. (2021). Divergent
adsorption behavior controlled by primary
coordination sphere anions in the metal–organic framework Ni_2_X_2_BTDD. J. Am. Chem. Soc..

[26] Wright A. M., Sun C., Dincă M. (2021). Thermal cycling of a MOF-based NO disproportionation
catalyst. J. Am. Chem. Soc..

[27] Denysenko D., Grzywa M., Jelic J., Reuter K., Volkmer D. (2014). Scorpionate-type
coordination in MFU-4l metal–organic frameworks: small-molecule
binding and activation upon the thermally activated formation of open
metal sites. Angew. Chem., Int. Ed..

[28] Sengupta D., Melix P., Bose S., Duncan J., Wang X., Mian M. R., Kirlikovali K. O., Joodaki F., Islamoglu T., Yildirim T., Snurr R. Q., Farha O. K. (2023). Air-stable Cu­(I)
metal–organic framework for hydrogen storage. J. Am. Chem. Soc..

[29] Jaramillo D. E., Reed D. A., Jiang H. Z. H., Oktawiec J., Mara M. W., Forse A. C., Lussier D. J., Murphy R. A., Cunningham M., Colombo V., Shuh D. K., Reimer J. A., Long J. R. (2020). Selective
nitrogen adsorption via backbonding in a metal–organic framework
with exposed vanadium sites. Nat. Mater..

[30] Liao P. Q., Li X. Y., Bai J., He C. T., Zhou D. D., Zhang W. X., Zhang J. P., Chen X. M. (2014). Drastic enhancement
of catalytic activity via post-oxidation of a porous MnII triazolate
framework. Chem.—Eur. J..

[31] Miyaura N., Yamada K., Suzuki A. (1979). A new stereospecific cross-coupling
by the palladium-catalyzed reaction of 1-alkenylboranes with 1-alkenyl
or 1-alkynyl halides. Tetrahedron Lett..

[32] Miyaura N., Suzuki A. (1995). Palladium-catalyzed cross-coupling reactions of organoboron
compounds. Chem. Rev..

[33] Denysenko D., Grzywa M., Tonigold M., Streppel B., Krkljus I., Hirscher M., Mugnaioli E., Kolb U., Hanss J., Volkmer D. (2011). Elucidating gating
effects for hydrogen sorption in
MFU-4-type triazolate-based metal–organic frameworks featuring
different pore sizes. Chem.—Eur. J..

[34] Schmieder P., Denysenko D., Grzywa M., Baumgärtner B., Senkovska I., Kaskel S., Sastre G., van Wüllen L., Volkmer D. (2013). CFA-1: the first chiral metal–organic framework
containing Kuratowski-type secondary building units. Dalton Trans..

[35] Liao P.-Q., Chen H., Zhou D.-D., Liu S.-Y., He C.-T., Rui Z., Ji H., Zhang J.-P., Chen X.-M. (2015). Monodentate hydroxide
as a super strong yet reversible active site for CO2 capture from
high-humidity flue gas. Energy Environ. Sci..

[36] Rieth A. J., Wright A. M., Skorupskii G., Mancuso J. L., Hendon C. H., Dincă M. (2019). Record-setting
sorbents for reversible water uptake
by systematic anion exchanges in metal–organic frameworks. J. Am. Chem. Soc..

[37] Sun W., Chen P., Zhang M., Ma J. X., Sun J. (2023). Locating hydrogen
positions for COF-300 by cryo-3D electron diffraction. Angew. Chem., Int. Ed..

[38] Schoedel A., Li M., Li D., O’Keeffe M., Yaghi O. M. (2016). Structures of metal–organic
frameworks with rod secondary building units. Chem. Rev..

[39] Zhang J.-P., Zhang Y.-B., Lin J.-B., Chen X.-M. (2012). Metal azolate
frameworks:
from crystal engineering to functional materials. Chem. Rev..

[40] Gándara F., Uribe-Romo F. J., Britt D. K., Furukawa H., Lei L., Cheng R., Duan X., O’Keeffe M., Yaghi O. M. (2012). Porous, conductive metal-triazolates and their structural
elucidation by the charge-flipping method. Chem.—Eur.
J..

[41] Zhu H.-L., Chen H.-Y., Han Y.-X., Zhao Z.-H., Liao P.-Q., Chen X.-M. (2022). A porous π–π stacking framework
with dicopper­(I) sites and adjacent proton relays for electroreduction
of CO2 to C2+ products. J. Am. Chem. Soc..

[42] Iliescu A., Andrews J. L., Oppenheim J. J., Dincă M. (2023). A solid Zn-ion
conductor from an all-zinc metal–organic framework replete
with mobile Zn2+ cations. J. Am. Chem. Soc..

[43] Miner E. M., Park S. S., Dincă M. (2019). High Li^+^ and Mg^2+^ conductivity in a Cu-azolate metal–organic framework. J. Am. Chem. Soc..

[44] Wang X. X., Guan D. H., Ma X. Y., Yuan X. Y., Zhu Q. Y., Deng H. T., Wang H. F., Xu J. J. (2025). Coordination-driven
crosslinking electrolytes for fast lithium-ion conduction and solid-state
sattery applications. Angew. Chem., Int. Ed..

[45] Zhao G., Xu L., Jiang J., Mei Z., An Q., Lv P., Yang X., Guo H., Sun X. (2022). COFs-based electrolyte
accelerates the Na^+^ diffusion and restrains dendrite growth
in quasi-solid-state organic batteries. Nano
Energy.

[46] Zhang R., Chen Z., Jiang J., Liao X., Guo J., Huang W., Liu J., Dang S., Xu J., Wang H., Zhou Z., Zhang K., Cheng P., Shi W. (2025). Ordered lithiophilic
crown ether sites in a two-dimensional coordination
network for dendrite-free lithium deposition. CCS Chem..

[47] Xiong Z., Gu L., Liu Y., Wang H., Shi L., Wu X., Liu L., Chen Z. (2024). Isoreticular regulation of two-dimensional redox-active
covalent organic framework cathodes for enhanced lithium-ion storage. CCS Chem..

[48] Jamal H., Khan F., Kim J. H., Kim E., Lee S. U., Kim J. H. (2024). Compact solid electrolyte interface
realization employing
surface-modified fillers for long-lasting, high-performance all-solid-state
Li-metal batteries. Small.

[49] Kim E., Jamal H., Jeon I., Khan F., Chun S. E., Kim J. H. (2023). Functionality of
1-butyl-2,3-dimethylimidazolium bromide
(BMI-Br) as a solid plasticizer in PEO-based polymer electrolyte for
highly reliable lithium metal batteries. Adv.
Energy Mater..

