# Supplementary material for: The Assembly of a High-Efficiency Tris-benzotriazolate-Based Metal–Organic Framework Solid-State Electrolyte
Source: ACS Cent Sci. 2025 Jun 27;11(7):1199–206. doi: 10.1021/acscentsci.5c00567 (PMC12291110; doi:10.1021/acscentsci.5c00567)
Supplement: Supplementary file 1 [file oc5c00567_si_001.pdf]

Supporting Information

The Assembly of a High-Efficiency Tris-benzotriazolate-Based Metal-Organic Framework Solid-State Electrolyte

Zhangyi Xiong[1,2⊥], Shitao Wu[3⊥], Liang Gu[1,2], Mengyang Zhai[1,2], Yuke Pan[1,2], Yanhang Ma[3]*, Zhijie Chen[1,2]*

[1]Stoddart Institute of Molecular Science, Department of Chemistry, Zhejiang Key Laboratory of Excited-State Energy Conversion and Energy Storage, State Key Laboratory of Silicon and Advanced Semiconductor Materials, Zhejiang University, Hangzhou 310058, P. R. China

[2]Zhejiang-Israel Joint Laboratory of Self-Assembling Functional Materials, ZJU-Hangzhou Global Scientific and Technological Innovation Center, Zhejiang University, Hangzhou 311215, P. R. China

[3]School of Physical Science and Technology & Shanghai Key Laboratory of High-resolution Electron Microscopy, ShanghaiTech University, Shanghai 201210, China

[⊥]These authors contributed equally to this work.

*Corresponding author. Email: zhijiechen@zju.edu.cn, mayh2@shanghaitech.edu.cn

Table of Contents

1. Materials and general procedures 3

2. Synthesis of Ligands 6

3. Synthesis of MOFs 17

4. 3D ED Measurements 19

5. Additional structural figures 24

6. Characterization 25

7. Topological analysis 33

8. Ionic conductivities of Cu-TTBT-X 36

9. Li metal battery and Na metal battery 80

10. References 86

1. Materials and general procedures

All reagents were obtained from commercial sources and used without further purification, unless otherwise noted.

Nuclear magnetic resonance (^{1}H NMR)

^{1}H NMR spectra were recorded using a Bruker Avance III DMX 400 spectrometer. ^{13}C NMR were recorded on a Bruker 600 MHz system.

Solid-state nuclear magnetic resonance (SSNMR)

^{7}Li SSNMR experiments were performed on a Bruker Avance III HD spectrometer with a wide-bore magnet [B_0 = 14.1 T, $\nu_0(^{1}H)$ = 600.0 MHz, $\nu_0(^{7}Li)$ = 233.3 MHz]. A Bruker 3.2 mm triple-resonance probe was used to perform magic angle spinning (MAS) experiments at a spinning rate of 15 kHz.

^{23}Na SSNMR experiments were performed on a Bruker Avance III HD spectrometer with a wide-bore magnet [B_0 = 14.1 T, $\nu_0(^{1}H)$ = 400.0 MHz, $\nu_0(^{23}Na)$ = 105.9 MHz]. A Bruker 3.2 mm triple-resonance probe was used to perform MAS experiments at a spinning rate of 8 kHz.

X-Ray Diffraction Analyses

Powder X-ray diffraction (PXRD) experiments were measured on a Rigaku smartlab x-ray diffractometer operating at 40 kV/30 mA using the Cu Kα line (λ = 1.5418Å). Data were measured over the range of 3-30° in 15°/min steps over two minutes.

Thermogravimetric analyses (TGA)

TGA was performed on TA-Q500 apparatus. Samples were heated from 50 °C to 600 °C at a rate of 5 °C/min under air with flow rate 40 mL/min.

N_2 Sorption Measurements

N_2 adsorption and desorption experiments on activated materials were measured at 77 K on a JW-BK200C instrument.

Inductively coupled plasma-mass spectrometry (ICP-MS)

ICP-MS experiment was conducted on NexION 2000 (PerkinElmer Health Science Inc.).

Time-of-flight secondary ion mass spectrometry (TOF-SIMS)

TOF-SIMS was conducted on IONTOF GmbH using Bi cluster ion source (30 KeV). The typical analysis area was ~50×50 μm^2.

X-ray photoelectron spectroscopy (XPS)

XPS experiments were performed with Thermo K-Alpha XPS at Shiyanjia Lab.

Scanning Electron Microscope (SEM)

SEM images were taken using a Hitachi SU8010 and ZEISS Sigma 360. Samples were dispersed in acetone solution and then deposited onto silicon wafer with specification of 10×10×0.65 mm.

Transmission electron microscopy (TEM)

The TEM images were either taken in a TVIPS XF-416 camera on a JEOL JEM-2100 Plus or a Cheetah-1800 camera on a JEOL JEM-F200, under acceleration voltage of 200 kV. The specimens were first dispersed in ethanol to make suspensions. 1~3 drops of the suspensions were dropped onto carbon film supported copper grids, and after the evaporation of the ethanol, the copper grids were transferred to a high-tilt (±70 °) specimen holder and inserted to microscope.

3D ED data collection, data processing

The 3D ED data were collected using a modified INSTAMATIC[1] program equipped on either a JEOL JEM-2100 Plus or a JEOL JEM-F200 transmission electron microscope. Each dataset contains *ca.* 300 frames which are taken using 0.5 s exposure time to cover a *ca.* 110° of specimen rotation. The datasets were processed and integrated into reflection files by an XDS[2] data processing program. 3D ED data were reconstructed and viewed using a PETS2.0[3] software.

***Ab initio* structure solution and refinement**

The initial structures were obtained by a SHELXT[4] program, followed by a structure refinement using SHELXL[5] program in OLEX2[6].

Molecular dynamic (MD) simulations

Molecular dynamic (MD) simulations were performed on the Forcite module adopting the COMPASS force field to calculate the mean square displacement (MSD) and diffusion coefficients of Li^+ ions and Na^+ ion in Cu-TTBT-Li and Cu-TTBT-Na with PC molecule present, respectively. Based on the molar ratio among the components, the amorphous boxes for Cu-TTBT-Li and Cu-TTBT-Na were composed of Cu-TTBT-Li, 80 PC, 100 Li^+ and Cu-TTBT-Na, 80 PC, 100 Na^+, respectively. The long-range electrostatic interactions were calculated according to the Ewald method. All the snapshots were carried out at the NPT pattern with a coupling constant of 500 ps. After that, the snapshots were performed at NVT pattern for 500 ps to obtain an equilibrium state and then date collection at NVT pattern for another 1000 ps.

The diffusion coefficients of Li^+ and Na^+ can be estimated from the slope of the MSD curves by the Einstein relationship:

$$D = \frac{1}{6N_{\mathrm{a}}} \lim_{t\to\infty} \left(\frac{d}{d_t}\right) \sum_{i=t}^{N_{\mathrm{a}}} \langle [r_i(t) - r_i(0)]^2 \rangle$$

where $r_i(t)$ and the $r_i(0)$ are the position of molecules at time t and reference time 0.

2. Synthesis of Ligands

2.1 General Information

All reagents were purchased from commercial suppliers and used without further purification. 4-Bromo-1,2-diaminobenzene, di-tert-butyl decarbonate, and bis(pinacolato)diboron were purchased from ShangHai BiDe pharmaceutical Technology Co., Ltd. Pd(dppf)Cl_2 and 1,3,5-trimethylbenzene was purchased from Anhui Zesheng Technology Co., Ltd. $HIO_4 \cdot 2H_2O$ and CH_3COOH was purchased from Sinopharm Chemical Reagent Co., Ltd. The sodium metal was purchased from Changgao New Materials Co., Ltd.

2.2 Synthetic Protocols for TTBT

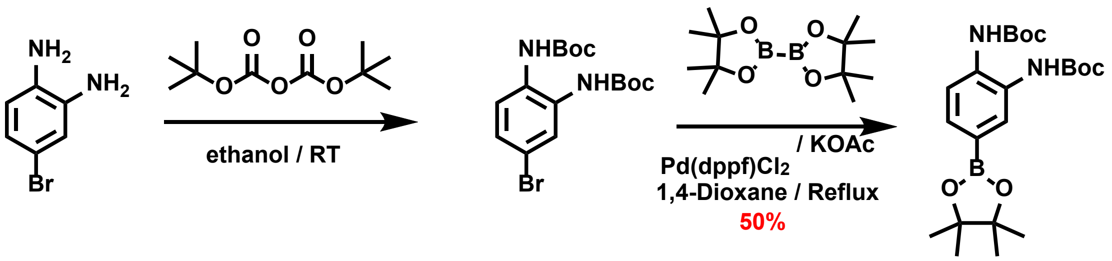

S1

Scheme S1. Synthesis of key intermediates **S1.**

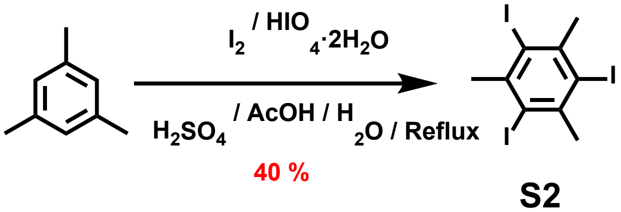

S2

Scheme S2. Synthesis of key intermediates **S2.**

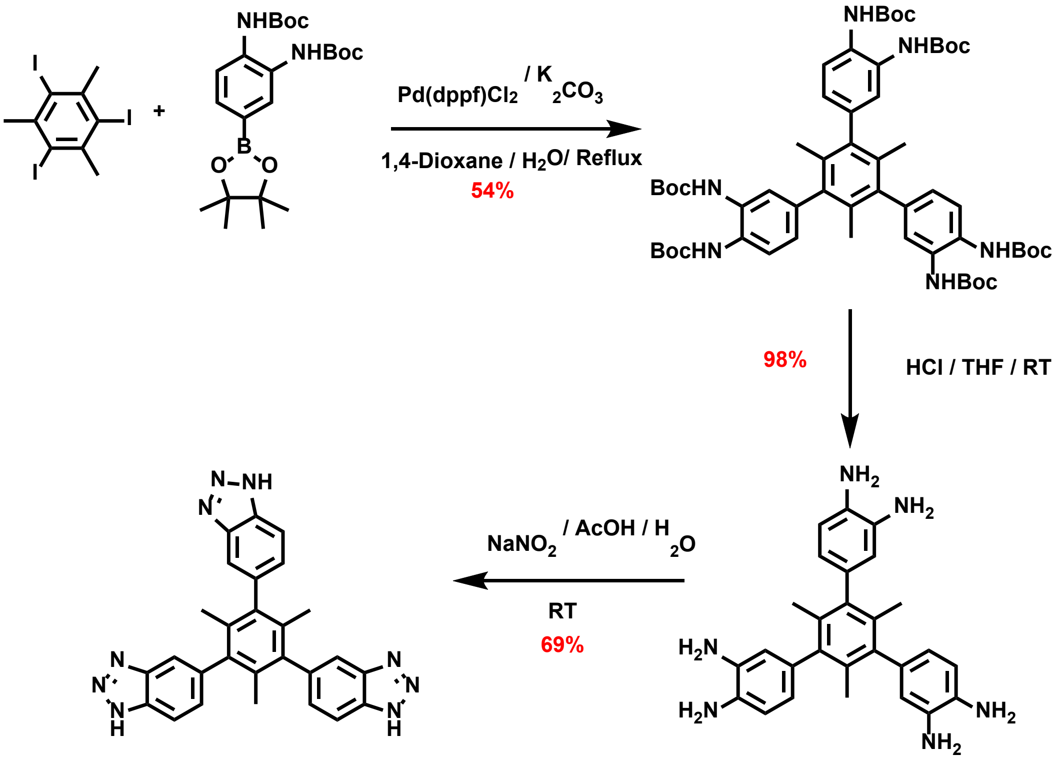

Scheme S3. Synthesis of H_3TTBT**.**

2.2.1) Synthesis of di-tert-butyl (4-bromo-1,2-phenylene)dicarbamate

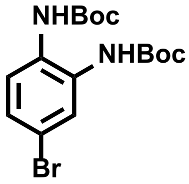

4-bromo-1,2-diaminobenzene (50 g, 0.27 mol), di-tert-butyl dicarbonate (150 g, 0.69 mol), and ethanol (500 mL) were added to a 1000 mL round-bottomed flask equipped with a magnetic stirrer bar. A condenser was attached to the flask and the mixture was stirred at room temperature for 24 h. The precipitate was collected by filtration and dried in an oven.

2.2.2) Synthesis of di-tert-butyl (4-(2,2,5,5-tetramethylborolan-1-yl)-1,2-phenylene)dicarbamate

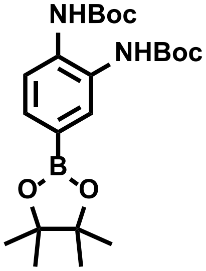

di-tert-butyl (4-bromo-1,2-phenylene)dicarbamate (3.87 g, 10 mmol), bis(pinacolato)diboron (10.16 g, 40 mmol), KOAc (3 g, 30 mmol), Pd(dppf)Cl_2 (365 mg, 0.5 mmol) was dissolved in 1, 4-dioxane (150 mL) in a 500 mL round-bottomed flask equipped with a magnetic stirrer bar. A condenser was attached to the flask and the mixture was degassed by bubbling N_2 for 30 min. The reaction was heated under reflux in an N_2 atmosphere for 48 h. After cooling to room temperature, 1, 4-dioxane was removed under reduced pressure and the residue was dissolved in CH_2Cl_2 (200 mL). The solution was washed with saturated brine solution (100 mL) and dried (Mg_2SO_4). After removing the solvent, the crude solid was washed with petroleum ether and filtrate to give the product as a pale purple solid. (2.16 g, yield = 50 %) ^{1}H NMR (400 MHz, DMSO-*d6*) δ 8.56 (d, *J* = 23.7 Hz, 2H), 7.77 (s, 1H), 7.59 (d, *J* = 8.1 Hz, 1H), 7.40 – 7.34 (m, 1H), 1.47 (s, 18H), 1.28 (s, 12H).

2.2.3) Synthesis of 2,4,6-triiodo-1,3,5-trimethylbenzene

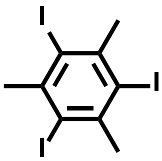

1,3,5-Trimethylbenzene (12.0 g, 0.1 mol), iodine (38.8 g, 0.305 mol), $HIO_4 \cdot 2H_2O$ (14.0 g, 0.0614 mol), CH_3COOH (100 mL), and H_2O (20 mL) were charged into a 250 mL round-bottomed flask equipped with a magnetic stirrer bar. After the mixture had been stirred, 3 mL of concentrated H_2SO_4 was added dropwise. The mixture was heated to reflux at 90 °C for 10 h, filtered with a vacuum line, repeatedly washed with distilled water, purified with solvent acetone, and suction

filtered to yield 2,4,6-triiodo-1,3,5-trimethylbenzene. (20.8 g, yield = 40 %) ^{1}H NMR (400 MHz, $CDCl_3$) δ 3.01 (s, 1H).

2.2.4) Synthesis of tetra-tert-butyl (5'-(3,4-bis((tert-butoxycarbonyl)amino)phenyl)-2',4',6'-trimethyl-[1,1':3',1''-terphenyl]-3,3'',4,4''-tetrayl)tetracarbamate

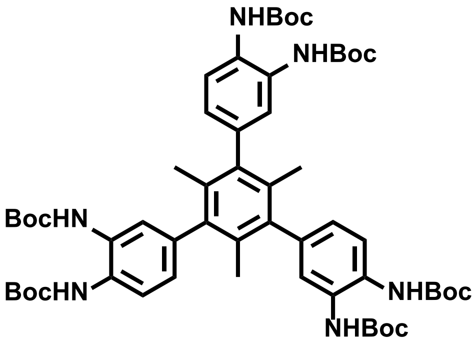

2,4,6-triiodo-1,3,5-trimethylbenzene (4.98 g, 10 mmol), di-tert-butyl (4-(2,2,5,5-tetramethylborolan-1-yl)-1,2-phenylene)dicarbamate (17.3 g, 40 mmol), Pd(dppf)Cl_2 (730 mg, 1 mmol), KOAc (6 g, 60 mmol), 1, 4-dioxane (200 mL), and H_2O (50 mL) were added to a 500 mL round bottom flask equipped with a magnetic stirrer bar. A condenser was attached to the flask and the mixture was degassed by bubbling N_2 for 30 min. The reaction was kept stirring at 105°C for three days. After cooling to room temperature, solvent was removed under reduced pressure. The crude solid was purified by chromatography (EA: PE=1:4) to give the product as a white powder. (5.6 g, yield = 54%) ^{1}H NMR (400 MHz, DMSO-d_6) δ 8.56 (d, J = 38.2 Hz, 6H), 7.54 (d, J = 6.2 Hz, 3H), 7.32 (s, 3H), 6.91 (d, J = 6.7 Hz, 3H), 1.66 (s, 9H), 1.47 (d, J = 15.1 Hz, 54H).

2.2.5) Synthesis of 5'-(3,4-diaminophenyl)-2',4',6'-trimethyl-[1,1':3',1''-terphenyl]-3,3'',4,4''-tetraamine

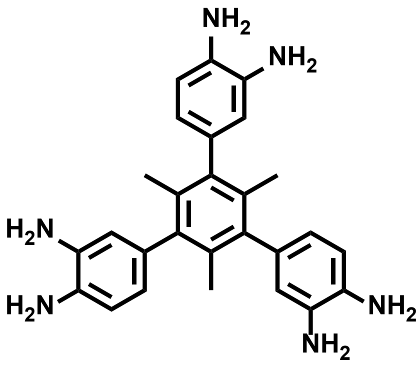

tetra-tert-butyl (5'-(3,4-bis((tert-butoxycarbonyl)amino)phenyl)-2',4',6'-trimethyl-[1,1':3',1''-terphenyl]-3,3'',4,4''-tetrayl)tetracarbamate (10 g, 10 mmol) was dissolved in THF (30 mL) in a 100 mL round-bottomed flask equipped with a magnetic stirrer bar. Concentrated HCl solution (15 ml) was added and the resulting mixture was stirred at room temperature overnight. The resulting mixture was filtrated and washed with THF (100 mL) to give the product as a pale-yellow solid (4.3 g, yield = 98%). ^{1}H NMR (400 MHz, DMSO-d_6) δ 7.17 (d, J = 8.5 Hz, 3H), 6.83 (s, 3H), 6.69 (d, J = 8.6 Hz, 3H), 1.66 (s, 9H).

2.3.2) Synthesis of 5,5',5''-(2,4,6-trimethylbenzene-1,3,5-triyl)tris(1H-benzo[d][1,2,3]triazole) (H_3TTBT)

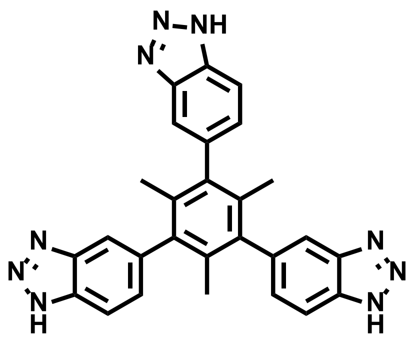

5'-(3,4-diaminophenyl)-2',4',6'-trimethyl-[1,1':3',1''-terphenyl]-3,3'',4,4''-tetraamine (4.71 g, 3.45 mmol), acetic acid (3.5 mL), and water (1 mL) were added to a 250 mL round-bottomed flask equipped with a magnetic stirrer bar. The mixture was cooled in an ice/water bath and a solution of sodium nitrite (0.29 g, 4.2 mmol) in water (1 mL) was added slowly while keeping the temperature below 10°C. The mixture was diluted with water (100 mL). The precipitate was harveted by filtration and washed with water (3.72 g, yield = 69%). ^{1}H NMR (400 MHz, DMSO-*d*6) δ 15.77 (s, 3H), 8.13 (s, 2H), 7.90 (s, 3H), 7.66 (s, 2H), 7.33 (d, J = 51.0 Hz, 3H), 1.67 (s, 9H). ^{13}C NMR (151 MHz, DMSO-d_6) δ 139.42, 139.27, 138.71, 133.34, 127.68, 115.89, 115.04, 19.76. ESI-HRMS calculated for $C_{27}H_{21}N_9$ $[M + H]^+$, m/z = 471.19, found 472.1992.

2.4. NMR Spectroscopy

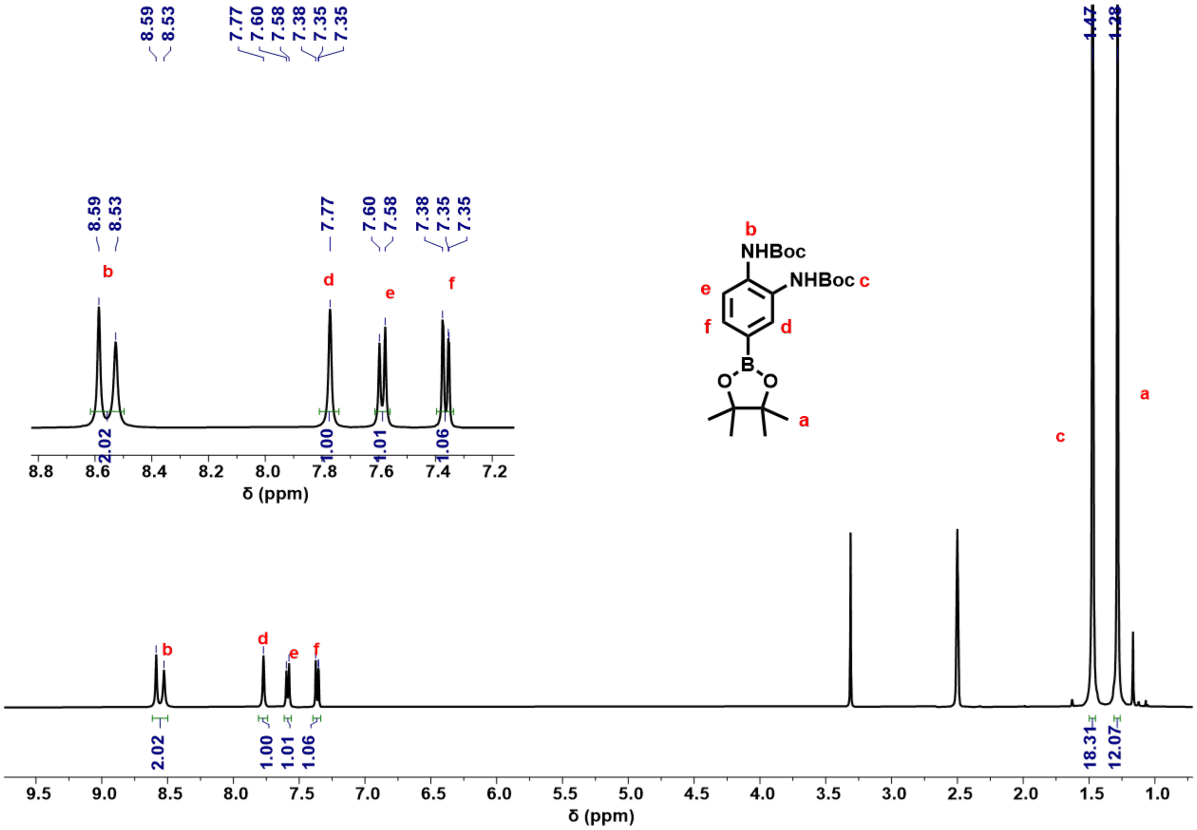

Figure S1. ^{1}H NMR Spectrum (400 MHz, DMSO-d_6, 298 K) of di-tert-butyl (4-(2,2,5,5-tetramethylborolan-1-yl)-1,2-phenylene)dicarbamate.

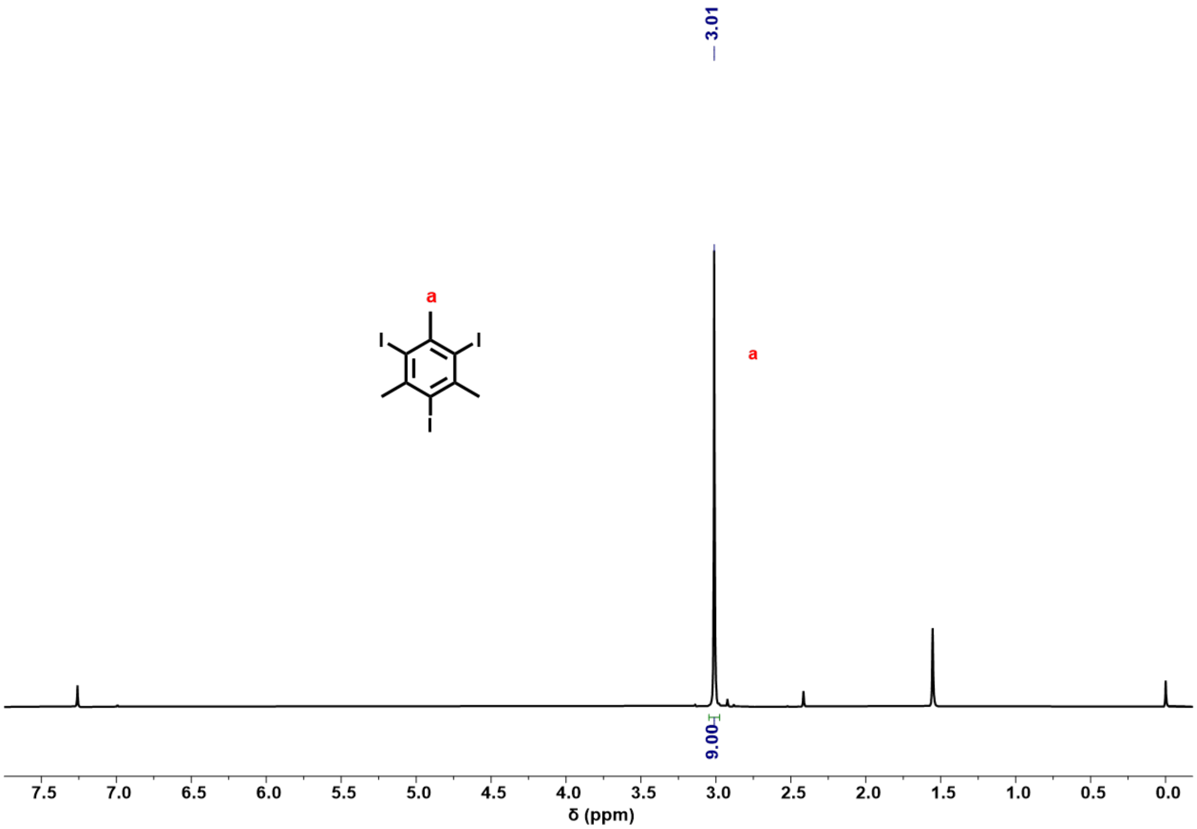

Figure S2. ^{1}H NMR Spectrum (400 MHz, $CDCl_3$, 298 K) of 2,4,6-triiodo-1,3,5-trimethylbenzene.

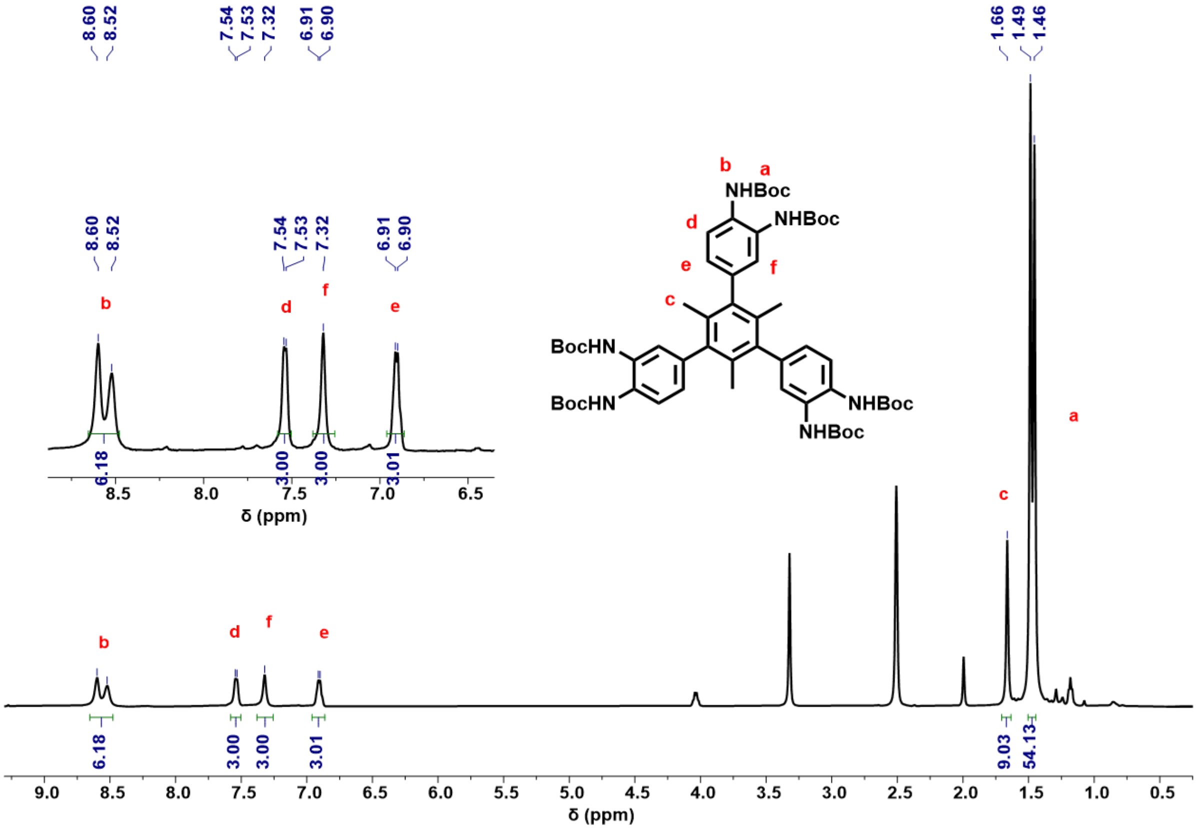

Figure S3. ^{1}H NMR Spectrum (400 MHz, DMSO-d_6, 298 K) of tetra-tert-butyl (5'-(3,4-bis((tert-butoxycarbonyl)amino)phenyl)-2',4',6'-trimethyl-[1,1':3',1''-terphenyl]-3,3'',4,4''-tetrayl)tetracarbamate.

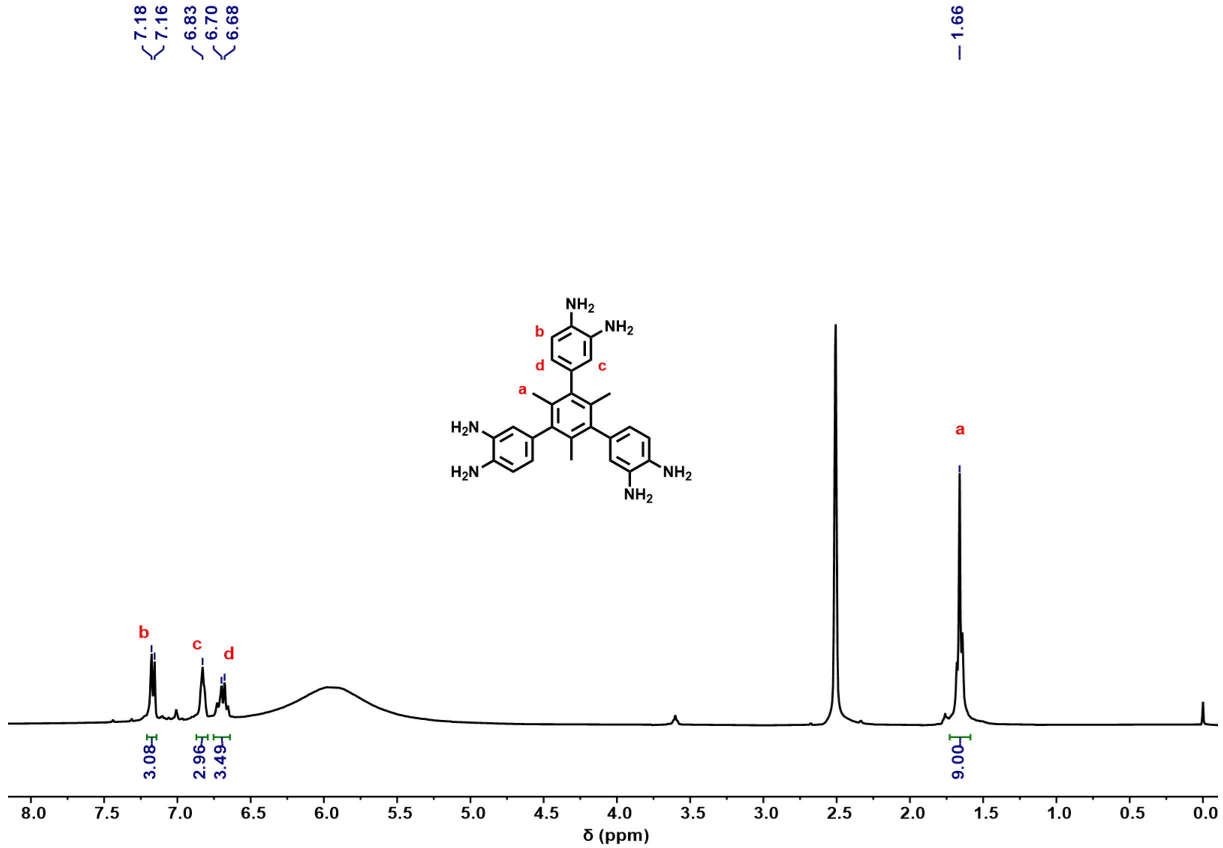

Figure S4. ^{1}H NMR Spectrum (400 MHz, DMSO-d_6, 298 K) of 5'-(3,4-diaminophenyl)-2',4',6'-trimethyl-[1,1':3',1''-terphenyl]-3,3'',4,4''-tetraamine.

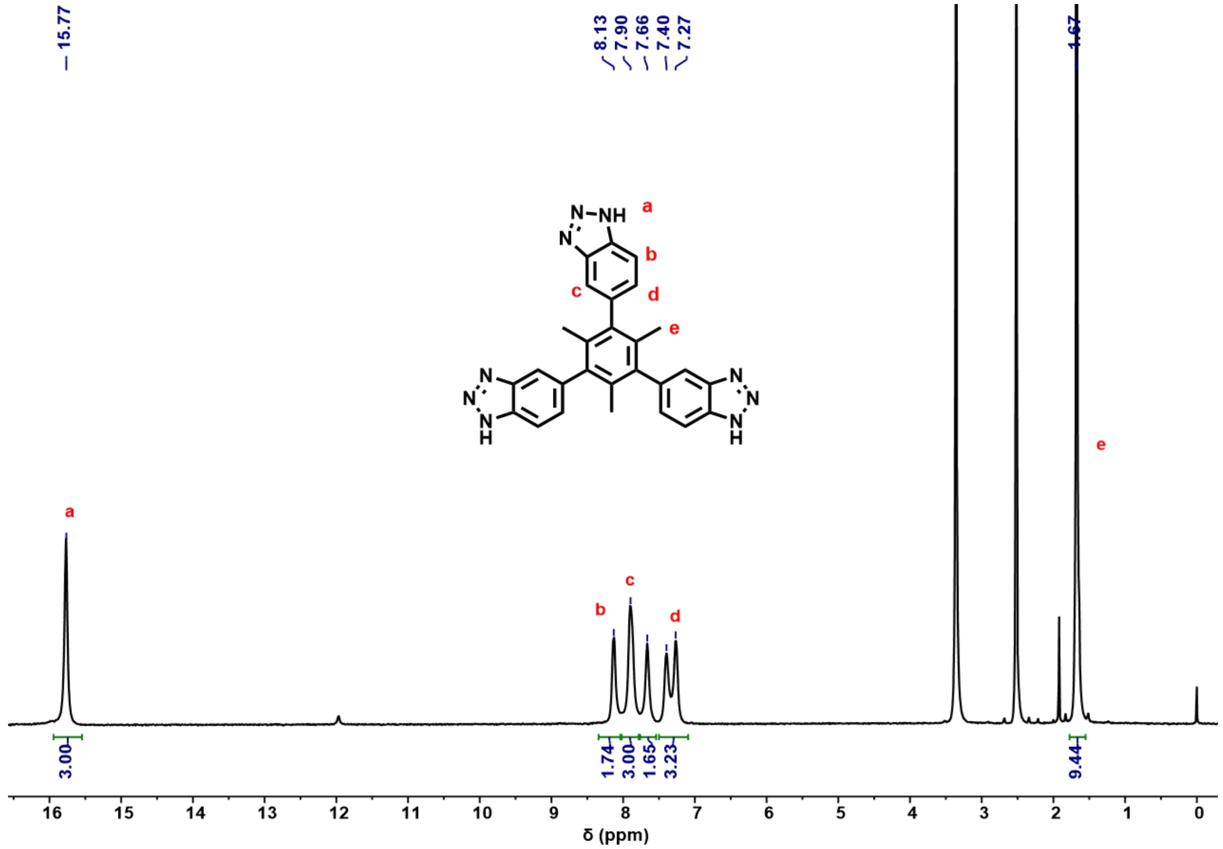

Figure S5. ^{1}H NMR Spectrum (400 MHz, DMSO-d_6, 298 K) of H_3TTBT.

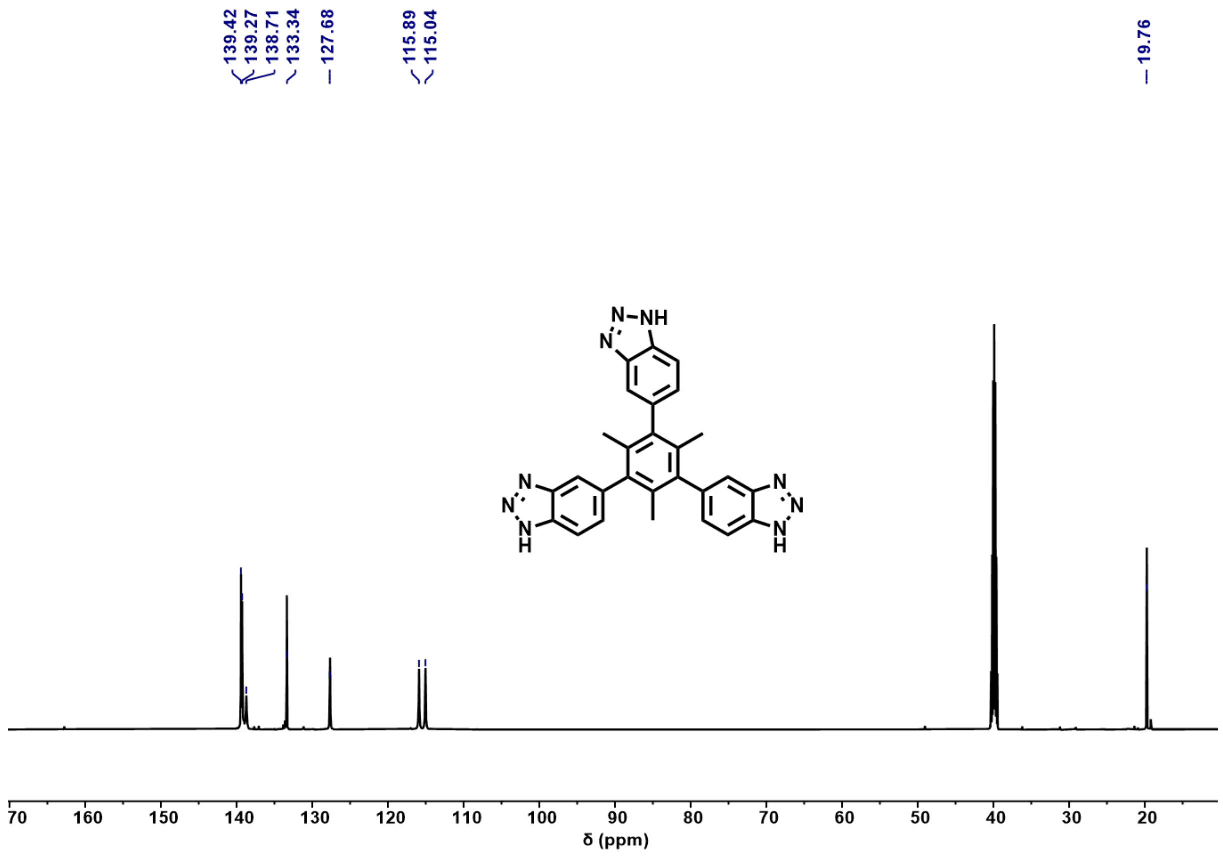

Figure S6. ^{13}C NMR Spectrum (400 MHz, DMSO-d_6, 298 K) of H_3TTBT.

3. Synthesis of MOFs

3.1 Synthesis of Cu-TTBT for the structural determination

5 mg H_3TTBT and 10 mg $CuCl_2·2H_2O$ were dissolved in 2 ml *N*, *N*-dimethylformamide (DMF) and 2 ml trifluoroacetic acid in a 15 mL vial. Then, the mixture was sonicated for 10 min. The resultant mixture was sealed and heated to 150 °C for 12 h. The yellow powder was obtained by centrifugation and washed with DMF and acetone, respectively.

3.2 Synthesis of Cu-TTBT on the gram scale

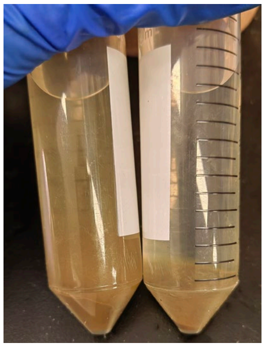

H_3TTBT (1 g, 0.031 mmol), $CuCl_2·2H_2O$ (2 g, 0.186 mmol), trifluoroacetic acid (40 mL, 10.57 mol), and 40 mL of DMF were added into a 200 mL Teflon autoclave. The autoclave was heated in an oven at 150 ℃ for 12 h and cooled to room temperature. The yellow powders were collected by centrifugation (8000 rpm for 5min). As-synthesized samples were washed with DMF and MeOH, respectively. Cu-TTBT MOF (~ 1 g) was obtained under air-dried. (yield: ~ 79%)

3.2 Synthesis of activated MOF

The as-synthesized Cu-TTBT powder was subjected to a Soxhlet extraction in MeOH for 2 days. The resulting powder was activated under dynamic vacuum at 100 °C.

3.3 Synthesis of Cu-TTBT-Li

100 mg of activated MOF were suspended in 0.1 M LiCl/tetrahydrofuran (THF) solution. The reaction mixture was stirred for 48 hours at room temperature. Then, the ion exchanged MOF was immersed in fresh THF (40 mL × 6) over two days to remove excess lithium salt in the pores. The

sample was soaked in anhydrous propylene carbonate (PC) (10 mL) for 24 h with stirring, vacuum filtered and dried under vacuum to obtain Cu-TTBT-Li.

3.4 Synthesis of Cu-TTBT-Na

100 mg of activated MOF were suspended in 2 mg/mL NaSCN/THF solution. The reaction mixture was stirred for 48 hours at room temperature. Then, the ion exchanged MOF was immersed in fresh THF (40 mL × 6) over two days to remove excess sodium salt in the pores. The sample was soaked in anhydrous PC (10 mL) for 24 h with stirring, vacuum filtered and dried under vacuum to obtain Cu-TTBT-Na.

3.5 Synthesis of Cu-TTBT-Mg

100 mg of activated MOF were suspended in 5 mg/mL $MgBr_2$/THF solution. The reaction mixture was stirred for 48 hours at room temperature. Then, the ion exchanged MOF was immersed in fresh THF (40 mL × 6) over two days to remove excess magnesium salt in the pores. The sample was soaked in anhydrous PC (10 mL) for 24 h with stirring, vacuum filtered and dried under vacuum to obtain Cu-TTBT-Mg.

4. 3D ED Measurements

Table S1. Crystal data collection and crystallographic data for Cu-TTBT, Cu-TTBT-Li, and Cu-TTBT-Na.

	Cu-TTBT	Cu-TTBT-Li	Cu-TTBT-Na
Formula	$C_{27}H_{18}Cu_2N_9$	$C_{27}H_{18}Cu_2N_9$	$C_{27}H_{18}Cu_2N_9$
Temperature (K)	293	293	293
Crystal system	Triclinic	Triclinic	Triclinic
Space group	$P\bar{1}$	$P\bar{1}$	$P\bar{1}$
a (Å)	11.24	11.14	11.28
b (Å)	15.16	15.56	15.49
c (Å)	15.49	15.59	15.33
α	106.91°	108.73°	107.71°
β	109.70°	105.21°	106.10°
γ	107.40°	109.36°	108.82°
V($Å^3$)	2134.048	2199.232	2195.876
Z	2	2	2
Diffractometer	JEOL JEM-F200	JEOL JEM-2100Plus	JEOL JEM-2100Plus
Radiation	Electron (λ = 0.02508 Å)	Electron (λ = 0.02508 Å)	Electron (λ = 0.02508 Å)
Tilting range	-42.50° ~ 59.73°	-58.38°~58.12° (dataset 1)<br>-58.71°~57.80° (dataset 2)<br>-60.65°~54.83° (dataset 3)	-49.79° ~ 29.44°
Frame step	0.3664°	~ 0.34°	0.3357°
Data reduction	XDS	XDS	XDS
Total reflection	11091	14696	6570
Unique reflections	3818	5751	2918
Max indices	(10, 14, 15)	(10, 16, 17)	(10, 14, 14)
Min indices	(-11, -14, 0)	(-11, -18, 0)	(-11, -15, 0)
Completeness	85.6 % (to 1.00 Å)	75.2 % (to 0.85 Å)	63.6 % (to 1.00 Å)

Solution method	SHELXT using OLEX2	SHELXT using OLEX2	SHELXT using OLEX2
Refinement method	SHELXL using OLEX2	SHELXL using OLEX2	SHELXL using OLEX2
R_1	15.58 %	24.53 %	25.65 %
wR_2	41.39 %	60.30 %	61.82 %
Rint	24.54 %	21.91 %	15.42 %
GooF	1.147	1.036	1.322
I/σ	3.6	3.0	4.3

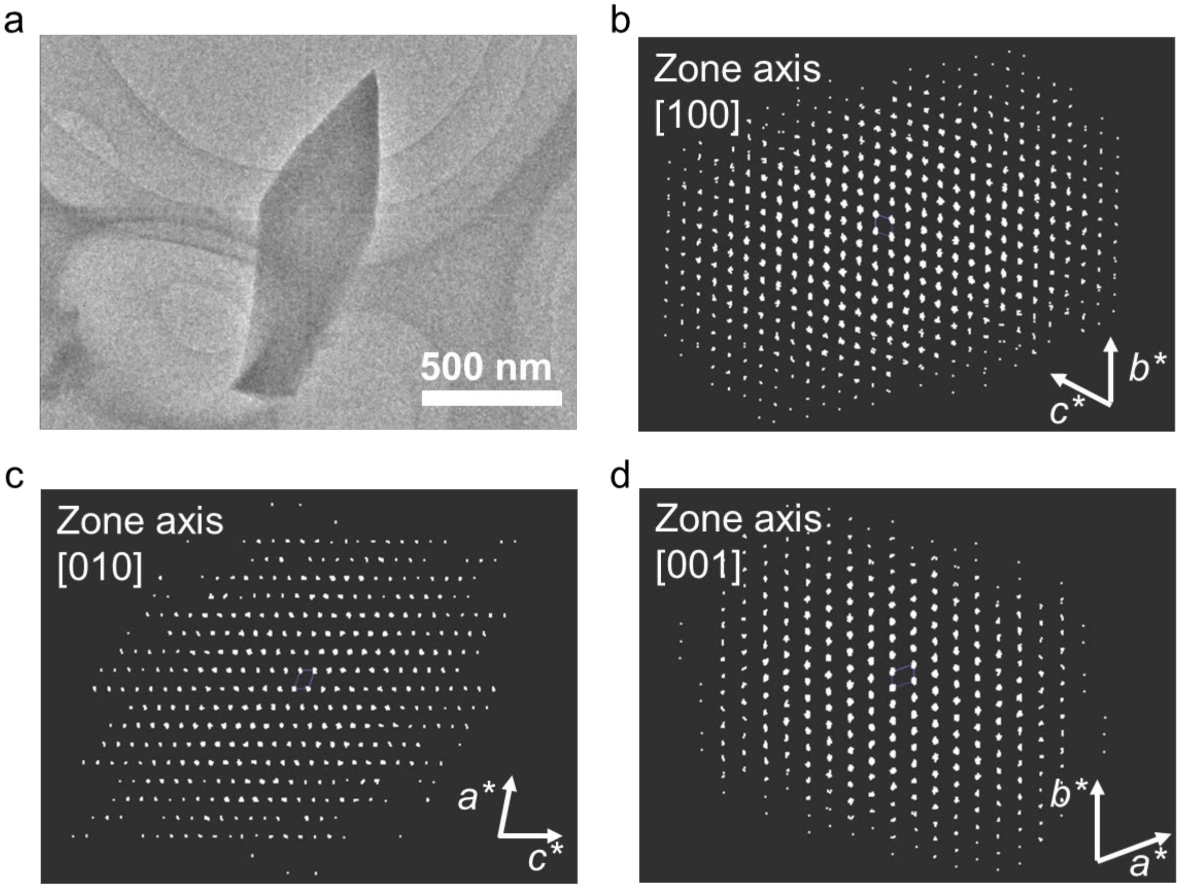

Figure S7. The projections of 3D ED data for Cu-TTBT viewed along the [100] (b), [010] (c), and [001] (d) directions, respectively.

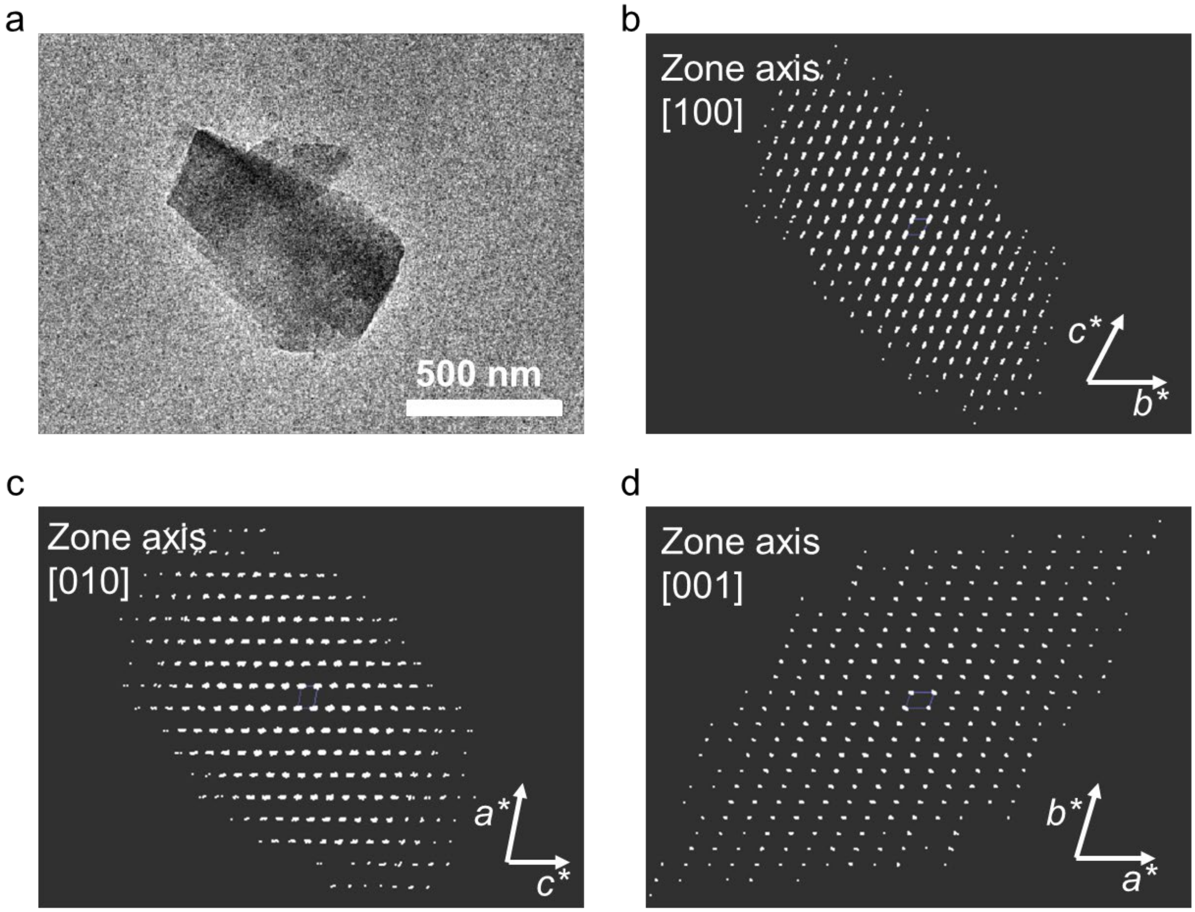

Figure S8. The projections of 3D ED data for Cu-TTBT-Li viewed along the [100] (b), [010] (c), and [001] (d) directions, respectively.

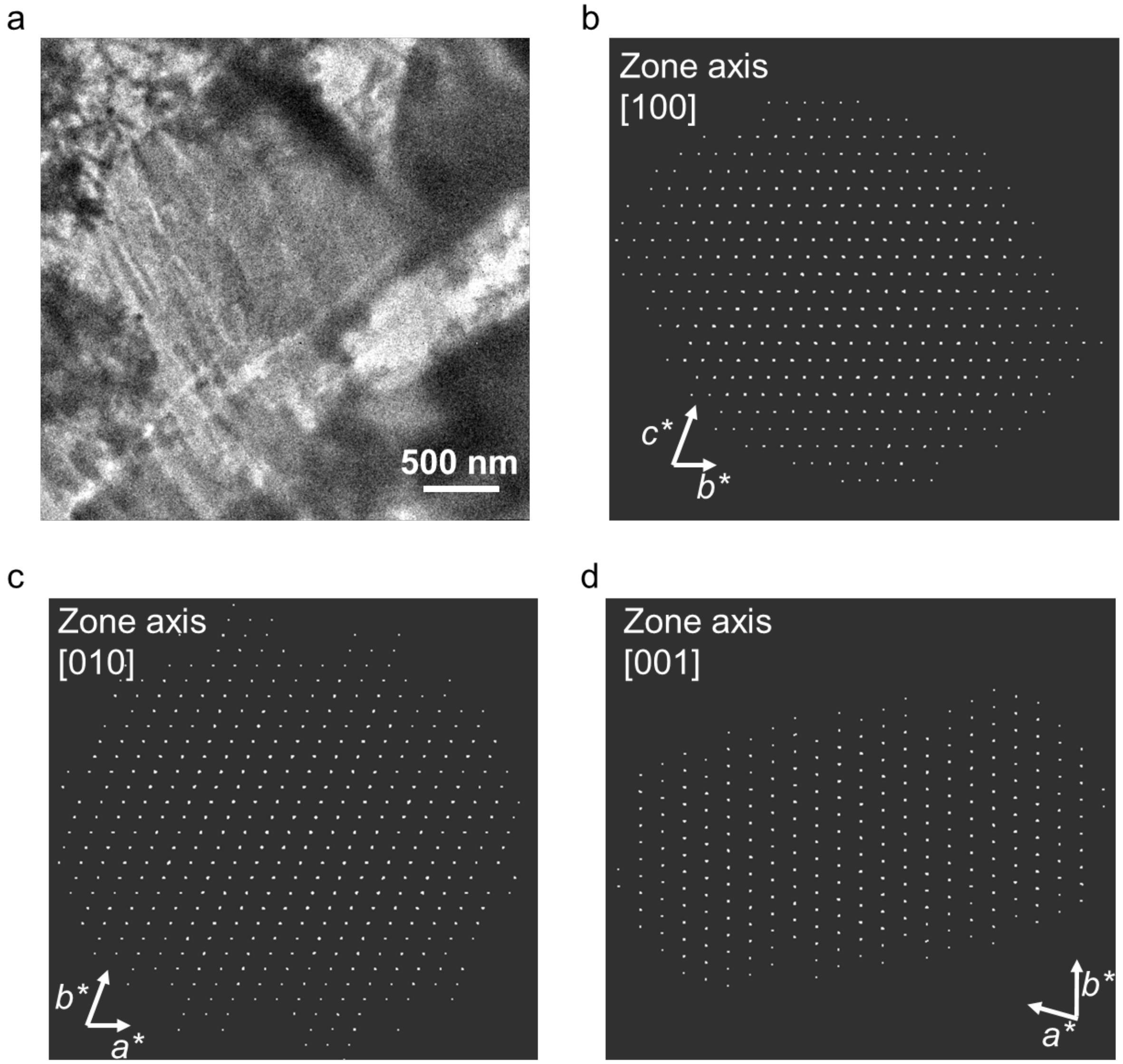

Figure S9. The projections of 3D ED data for Cu-TTBT-Na viewed along the [100] (b), [010] (c), and [001] (d) directions, respectively.

5. Additional structural figures

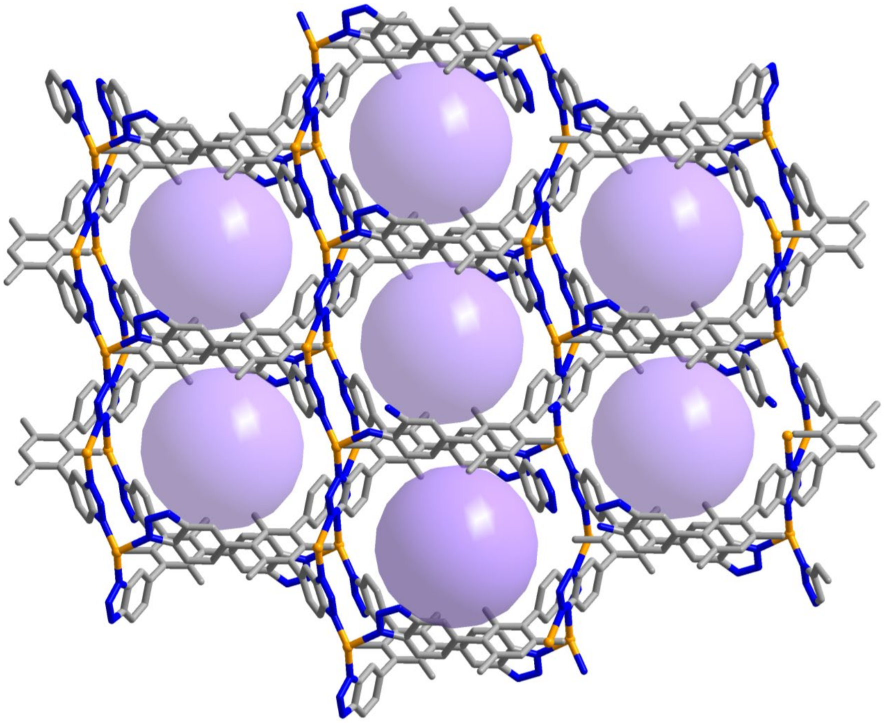

Figure S10. The pore structure of Cu-TTBT.

6. Characterization

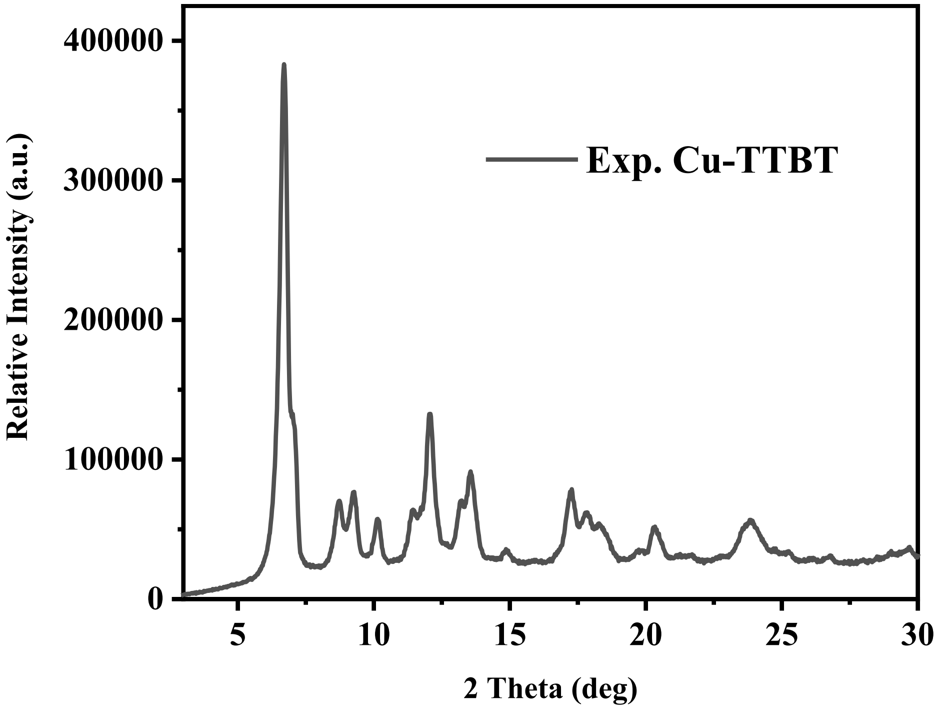

Figure S11. The PXRD pattern of Cu-TTBT using the Cu Kα line (λ = 1.5418Å).

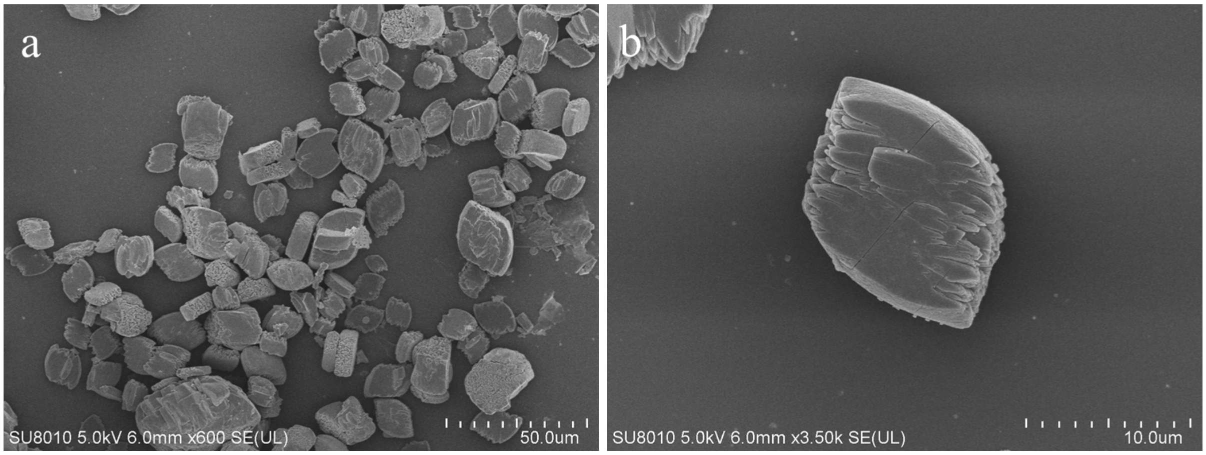

Figure S12. The SEM images of Cu-TTBT.

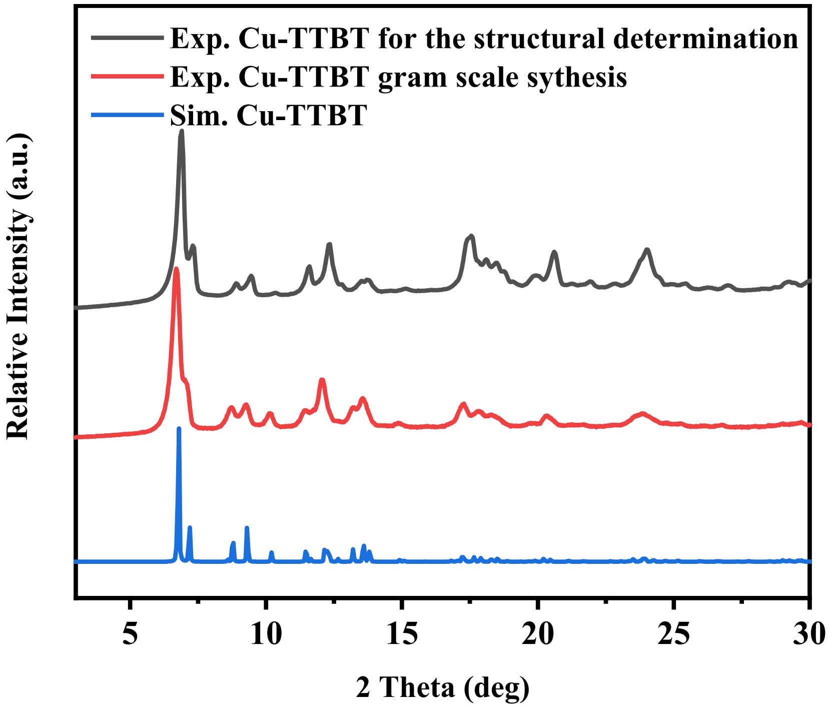

Figure S13. Experimental and simulated PXRD patterns of Cu-TTBT using the Cu Kα line (λ = 1.5418Å).

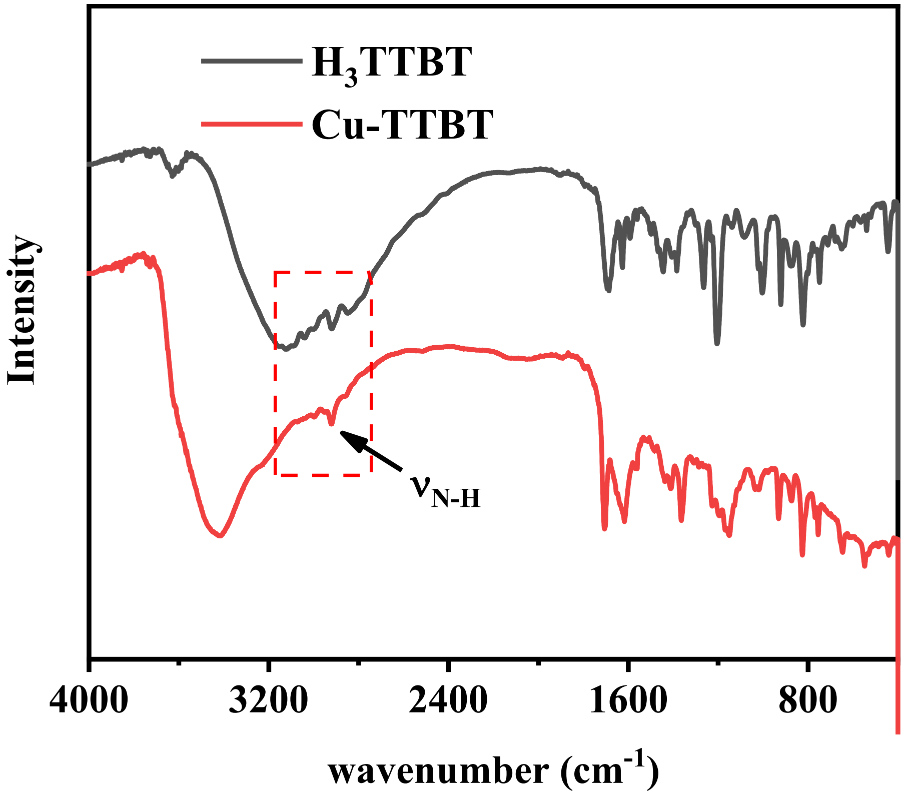

Figure S14. FTIR spectra of Cu-TTBT and H_3TTBT. Cu-TTBT and H_3TTBT exhibit absorption peak at ~3000 cm^{-1}, suggesting the ligands in Cu-TTBT MOF remain protonated.[7]

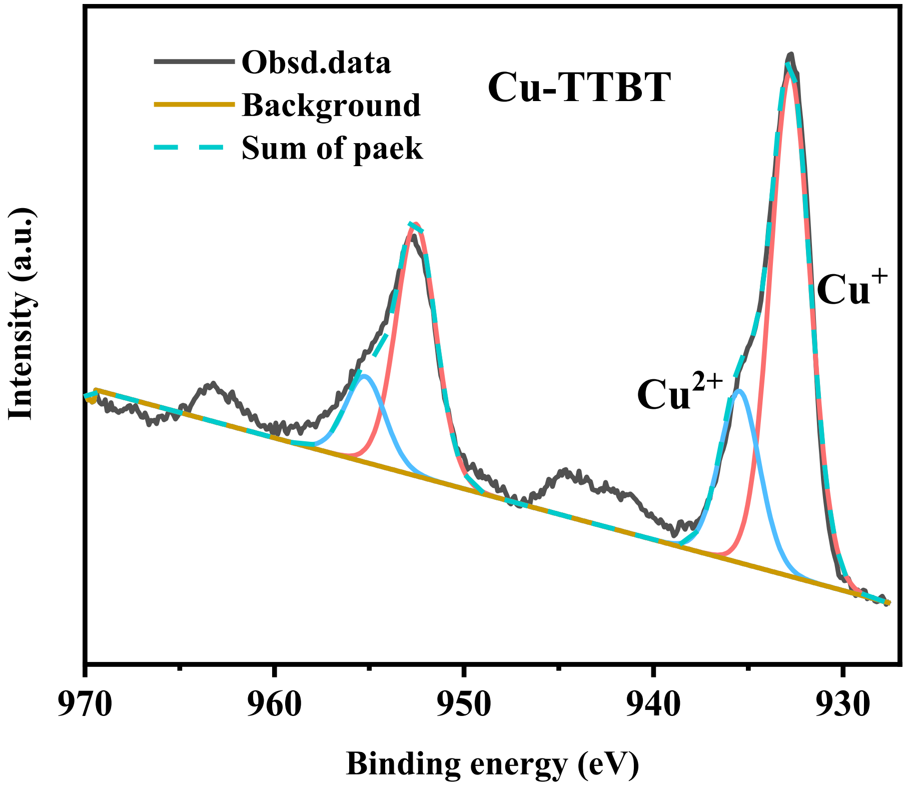

Figure S15. Cu 2p XPS spectra of Cu-TTBT.

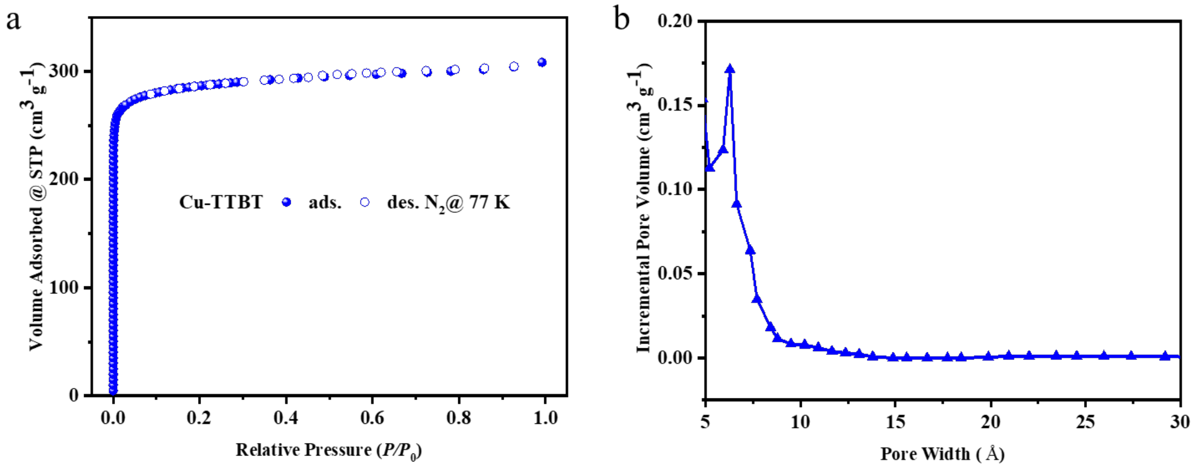

Figure S16. (a) N_2 adsorption and desorption isotherms at 77 K of Cu-TTBT. (b) Pore size distribution of Cu-TTBT from a nonlocal density functional theory (NLDFT) model with cylinder pore geometry.

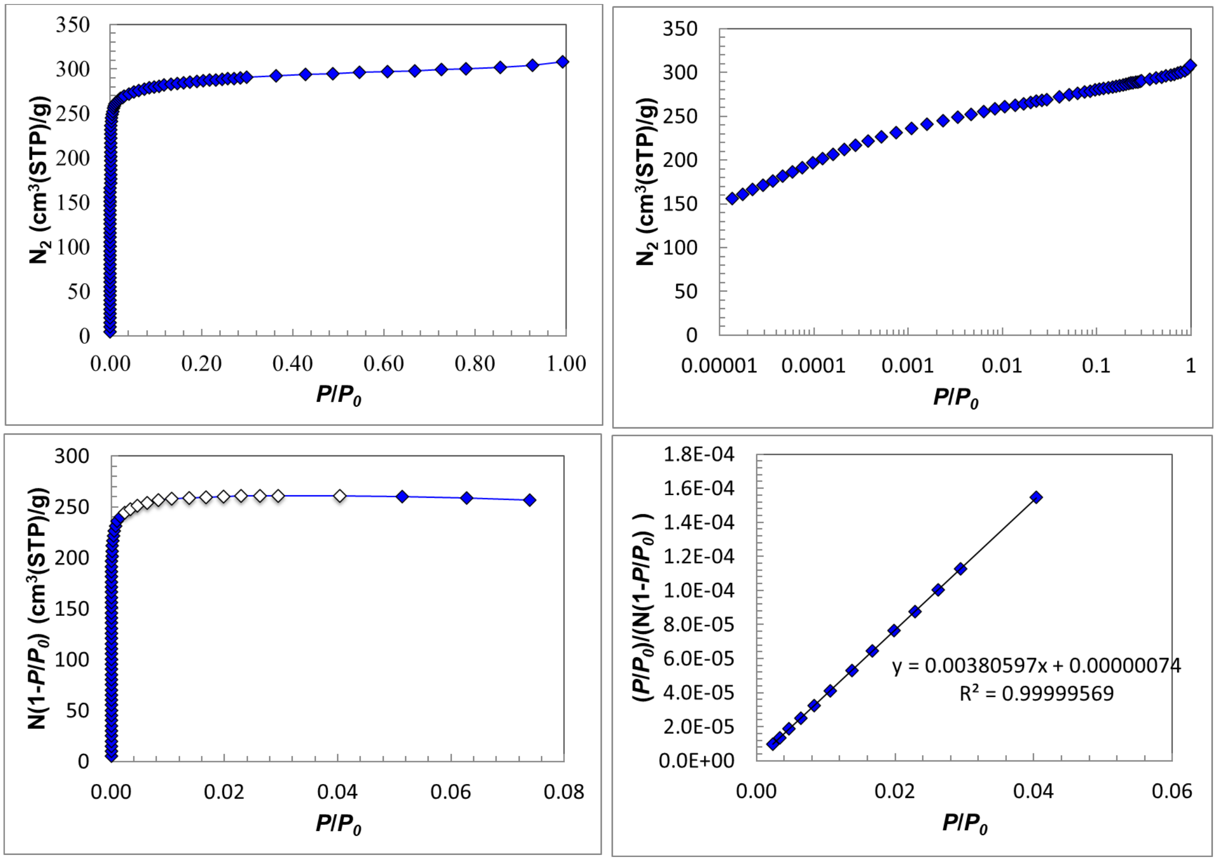

Figure S17. Experimental N_2 adsorption isotherm at 77 K of Cu-TTBT, and the region of selected points (white) used for the calculation of BET area, fulfilling all four BET criteria with R^2 higher than 0.995.

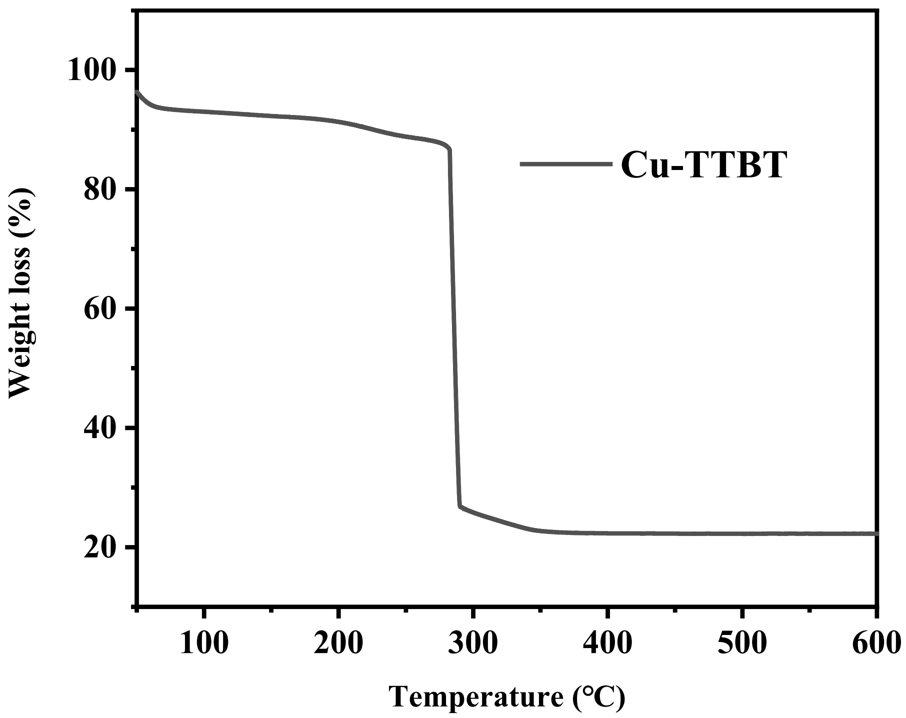

Figure S18. Thermogravimetric curve tested at air flow of Cu-TTBT.

7. Topological analysis

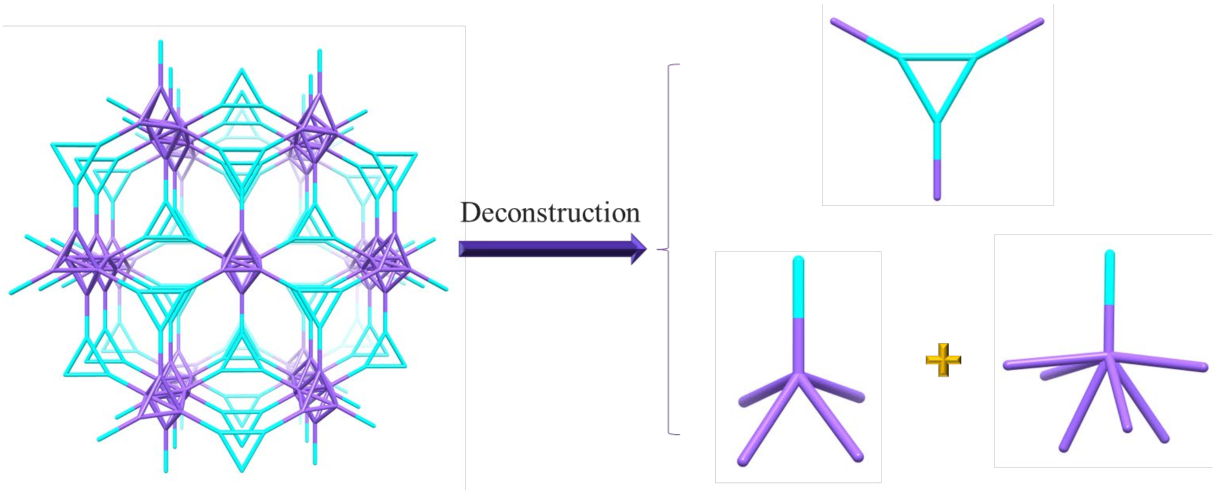

Figure S19. Topological analysis of Cu-TTBT.

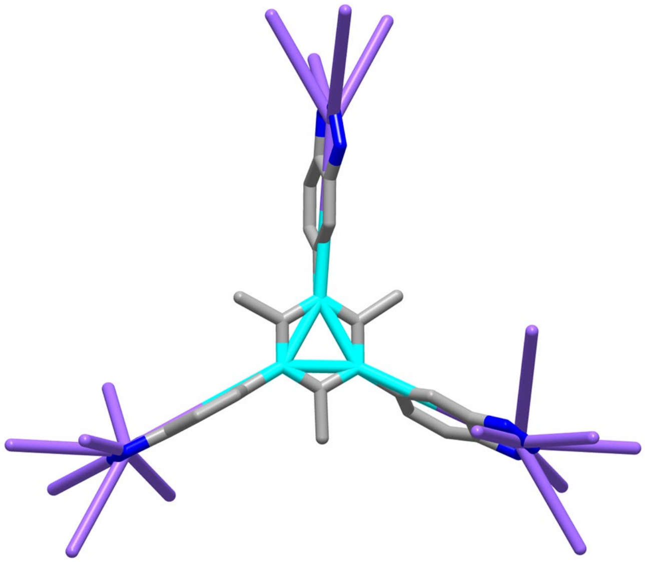

Figure S20. The central triangle can be divided into two kinds of 3-connected nodes due to different coordination environments of ligands. One 3-connected node connects to a 5-connected node and the other 3-connected node connects to a 7-connected node.

Point symbol for net: {3.8^2}3{3^5.4.8^2.9^2}{3^8.4^5.5^2.8^3.9^3}2

3^2,5,7-c net with stoichiometry (3-c)2(3-c)(5-c)(7-c)2; 4-nodal net

Topological terms for each node:

(a) Point symbol: {3^8.4^5.5^2.8^3.9^3}

Extended point symbol: [3.3.3.3.3.3.3.3.4.4.4.4.4(2).5(2).5(2).8.8.8.9.9.9(2)]

Coordination sequence: 8 22 48 91 168 272 406 581 814 1096

(b) Point symbol: {3^5.4.8^2.9^2}

Extended point symbol: [3.3.3.3.3.4(2).8.8.9(2).9(2)]

Coordination sequence: 6 18 40 82 156 256 389 563 796 1068

(c) Point symbol: {3.8^2}

Extended point symbol: [3.8.8(2)]

Coordination sequence: 4 12 34 77 142 241 380 557 775 1047

(d) Point symbol: {3.8^2}

Extended point symbol: [3.8.8]

Coordination sequence: 4 10 32 74 140 234 375 553 766 1040

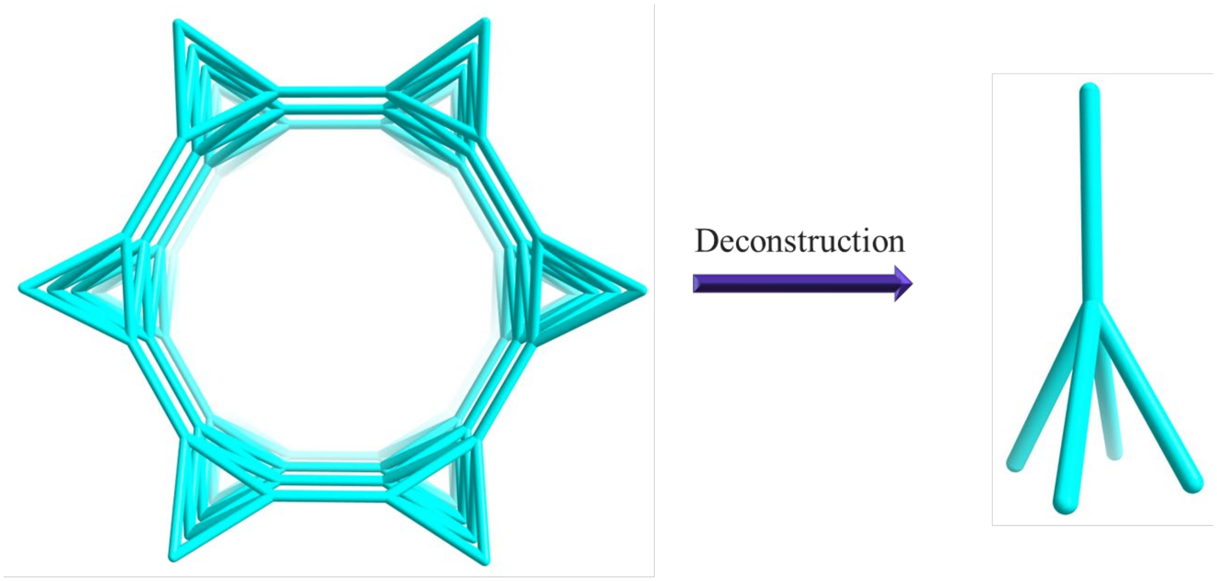

MIT-20
sqc1539

Figure S21. Topological analysis of MIT-20.

Point symbol for net: {3^3.4^2.5.6^4}

5-c net; uninodal net

Topological terms for each node:

(a) Point symbol: {3^3.4^2.5.6^4}

Extended point symbol: [3.3.3.4.4.5.6(2).6(2).6(2).6(2)]

Coordination sequences: 6 18 44 86 160 264 401 581 816 1098

----

8. Ionic conductivities of Cu-TTBT-X

8.1 Electrochemical Impedance Spectroscopy

Electrochemical impedance spectroscopy (EIS) was performed on Biologic VSP 3e electrochemical workstation with a frequency range of 1 MHz-1 Hz. In detail, the pellet was prepared in a split-able test cell by pressing powder into a PTFE cylindrical with 10 mm diameter. The pellet was then sandwiched between two stainless steel blocking electrodes. The thickness of the pellet controlled between 0.3 mm and 0.6 mm.

The ionic conductivity was calculated using equation (1):

$$\sigma = \frac{L}{RA}$$

where σ is ionic conductivity, L is the pellet thickness, A is the pellet area, and R is the resistance obtained by fitting the Nyquist plot to the model circuit shown in Scheme S4.

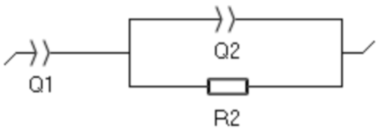

Scheme S4. The equivalent circuit used for fitting impedance spectra. R2 is a resistor and Q1 and Q2 are constant phase elements which are imperfect capacitors.

The activation energy was calculated with the Nernst-Einstein relation:

$$\sigma = \frac{\sigma_0}{T} e^{-\frac{E_a}{KT}}$$

where σ_0 is a pre-exponential factor, T is the temperature, E_a is the activation energy and k is the Boltzmann constant.

8.2 Electrochemical measurements of transference numbers

MOF and Polytetrafluoroethylene (PTFE) with a weight ratio of 9:1 was mixed in ethanol. After removing ethanol, the obtained solid powder was pressed into a pellet (19 mm diameter) under 3 MPa pressure for 30 s. The pellet was immersed in anhydrous propylene carbonate over three days

before characterization. The pellet was assembled into the cell between two lithium chips of Φ = 16 mm in an Ar filled glovebox.

The direct current (DC) polarization measurement was performed on cells with 50 mV and the current response was measured for 60 min at 25°C. The EIS was measured with an input voltage amplitude of 100 mV between 1 MHz and 1 Hz before and after the DC polarization.

Li^+ transference number was calculated using the Bruce-Vincent-Evans (BVE) equation (2):

$$t_{Li^+} = \frac{I_S(\Delta V - I_0 R_0)}{I_0(\Delta V - I_S R_S)}$$

where ΔV is the applied potential, I_0 and I_s are the initial and steady-state current, and R_0 and R_S are the initial and steady-state interfacial resistance, which are determined by fitting the Nyquist plot using EC-Lab software.

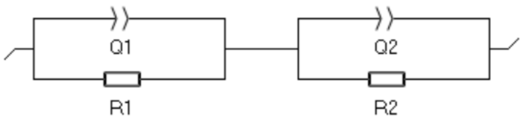

Scheme S5. The equivalent circuit used for fitting impedance spectra. R1 and R2 are resistors and Q1 and Q2 are constant phase elements which are imperfect capacitors.

8.3 Linear sweep voltammetry measurements

Linear sweep voltammetry (LSV) measurements were conducted on stainless steel|SSE|Li cells with a voltage range from the open-circuit voltage to 5 V at a scan rate of 0.5 mV s^{-1}.

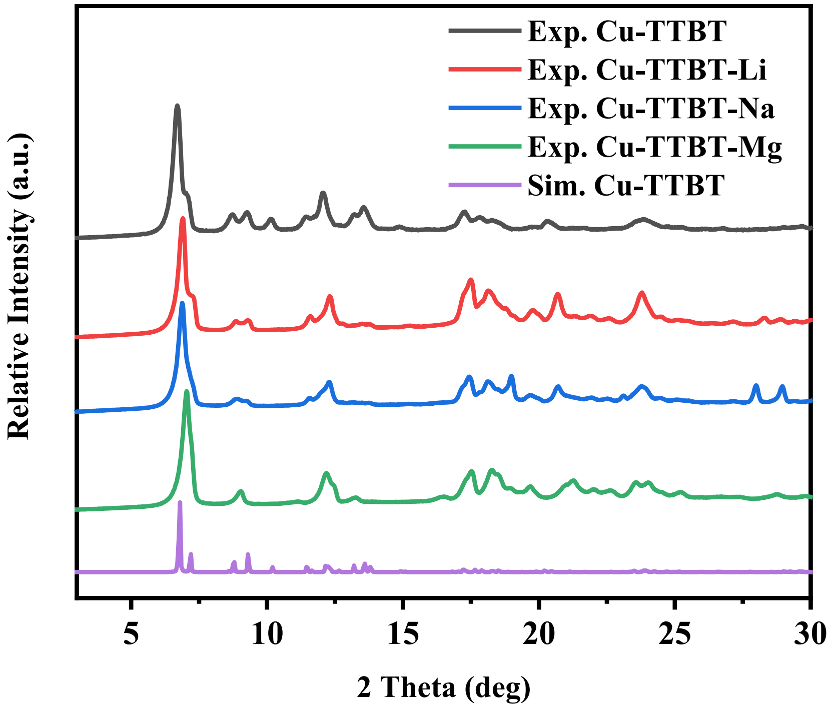

Figure S22. Experimental PXRD patterns of Cu-TTBT, Cu-TTBT-Li, Cu-TTBT-Na, and Cu-TTBT-Mg, in comparison to the simulated pattern from the crystal structure.

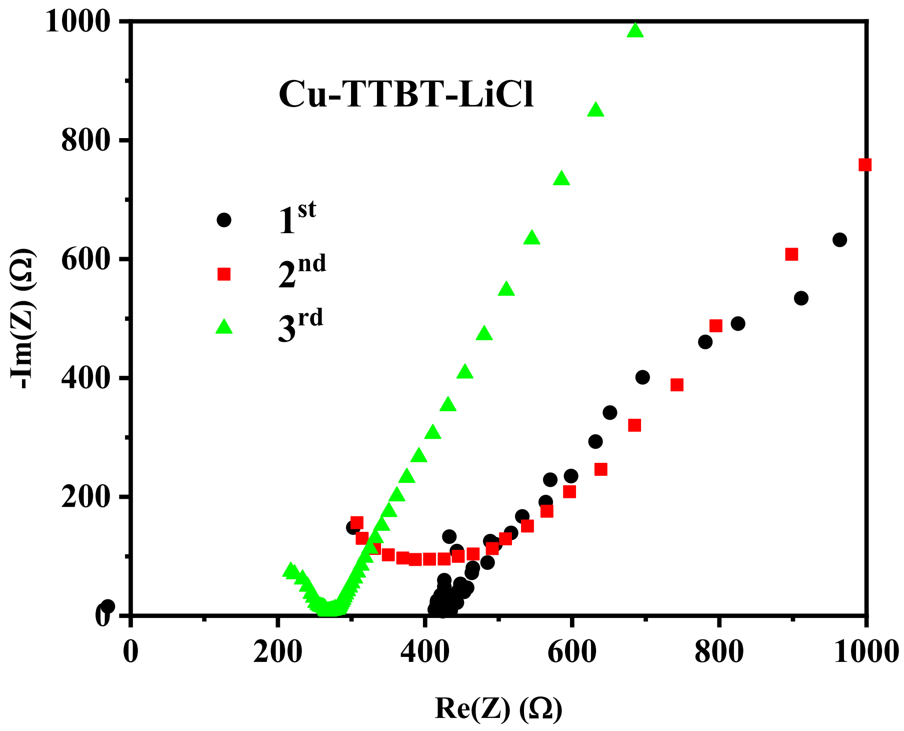

.

Figure S23. Ionic conductivity of Cu-TTBT-Li at 25°C collected from three different batches.

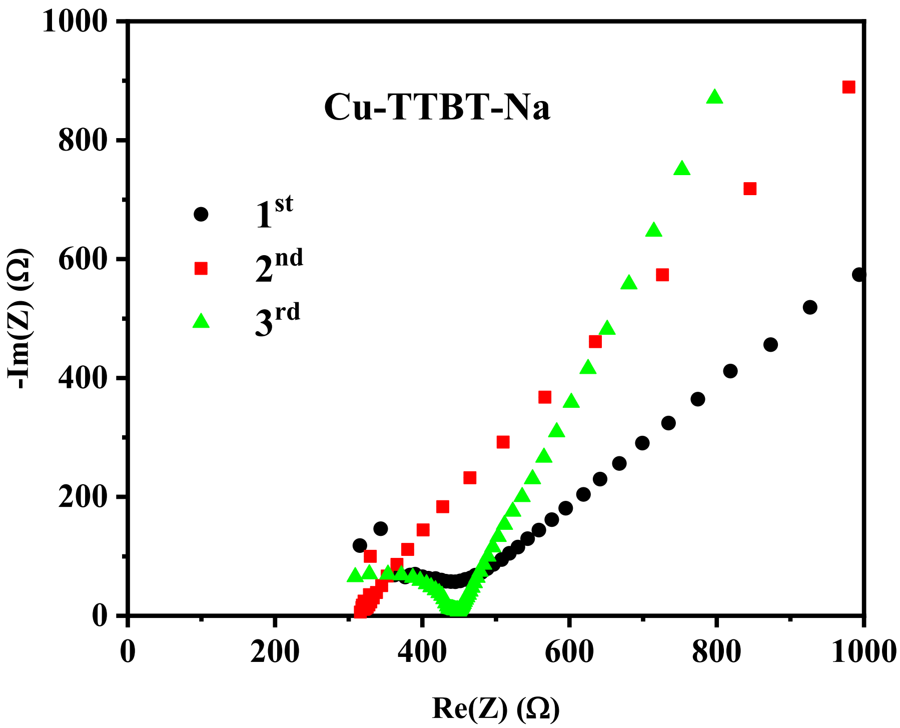

Figure S24. Ionic conductivity of Cu-TTBT-Na at 25°C collected from three different batches.

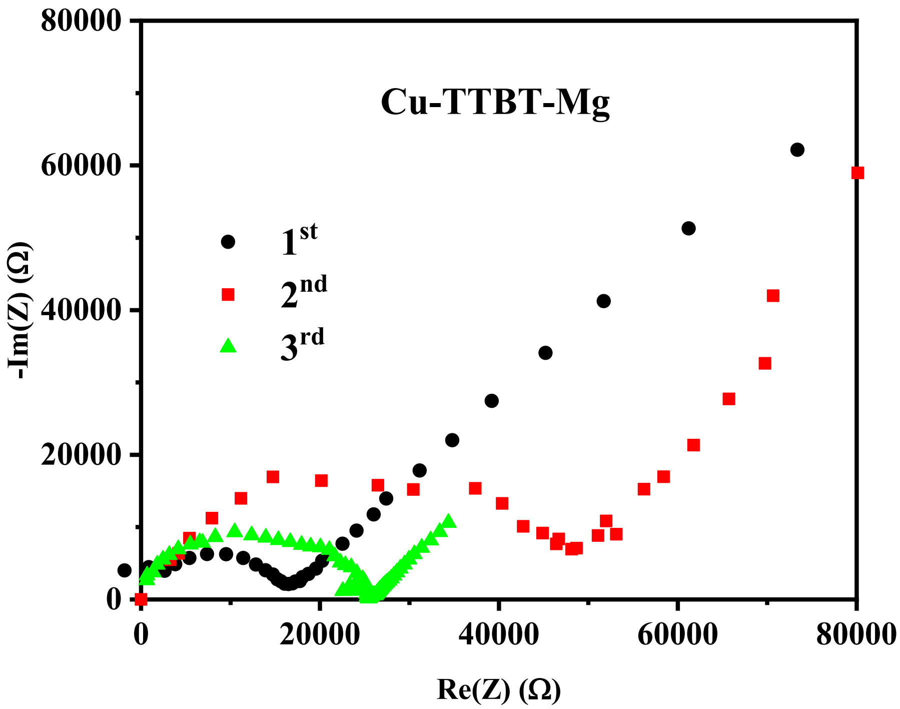

Figure S25. Ionic conductivity of Cu-TTBT-Mg at 25°C collected from three different batches.

Table S2. The summary of ionic conductivity of Cu-TTBT-X at 298 K collected from different batches.

	Batch	T/ °C	Pellet thickness/ cm	Pellet area/ cm^2	Resistance/ Ohm	Ionic conductivity/ S cm^{-1}
Li	1st	25	0.0384	0.785	267	1.83E-04
Li	2nd	25	0.0621	0.785	387.1	2.04E-04
Li	3rd	25	0.0621	0.785	422	1.87E-04
Na	1st	25	0.0384	0.785	444	1.10E-04
Na	2nd	25	0.0304	0.785	350.2	1.11E-04
Na	3rd	25	0.0309	0.785	350.2	1.12E-04
Mg	1st	25	0.0204	0.785	2196	1.18E-05
Mg	2nd	25	0.0504	0.785	15000	4.28E-06
Mg	3rd	25	0.0468	0.785	50322	1.18E-06

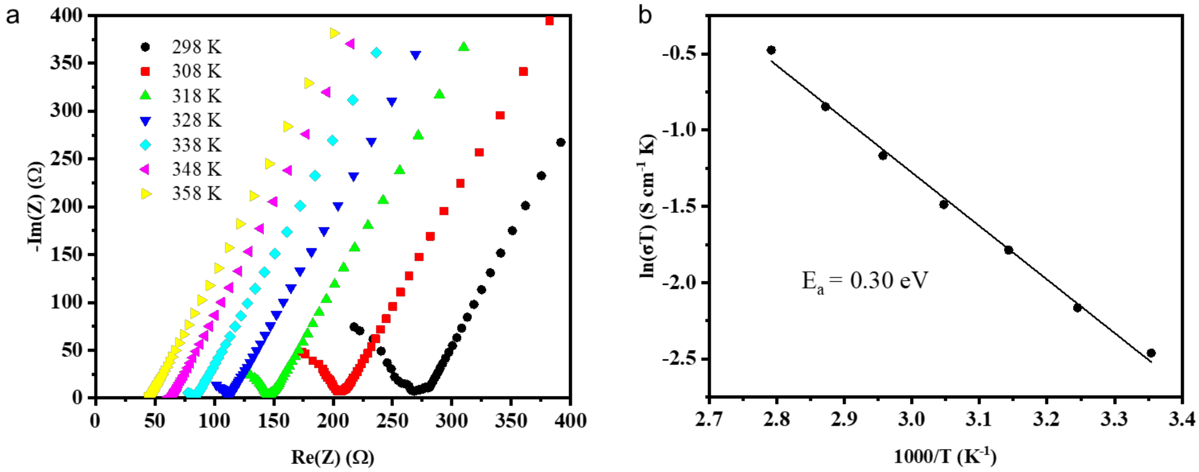

Figure S26. (a) Nyquist plots of Cu-TTBT-Li at different temperature. (b) Temperature-dependent conductivity plots for Cu-TTBT-Li in the range of 25 °C to 85 °C with 10 °C intervals.

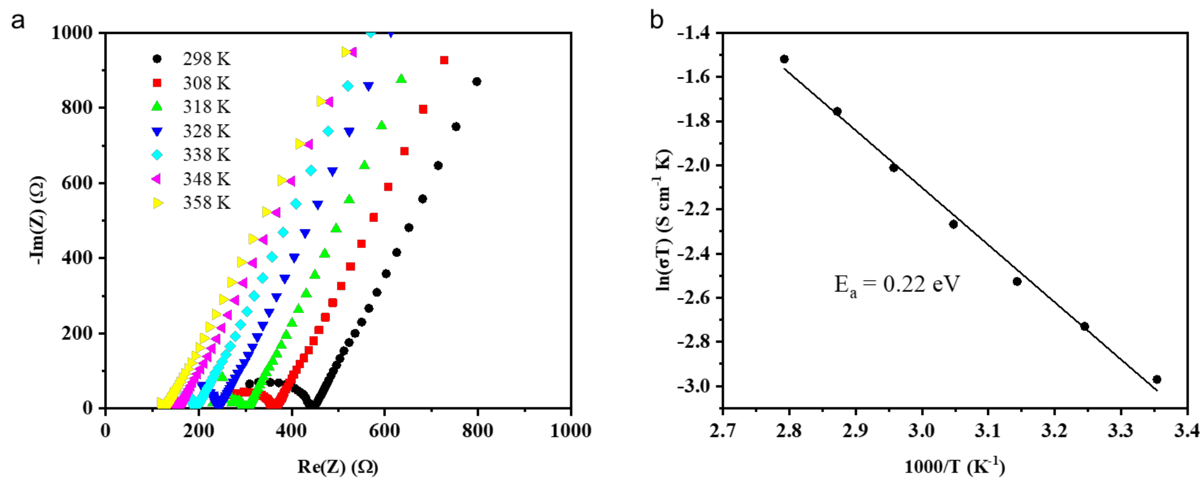

Figure S27. (a) Nyquist plots of Cu-TTBT-Na at different temperature. (b) Temperature-dependent conductivity plots for Cu-TTBT-Na in the range of 25 °C to 85 °C with 10 °C intervals.

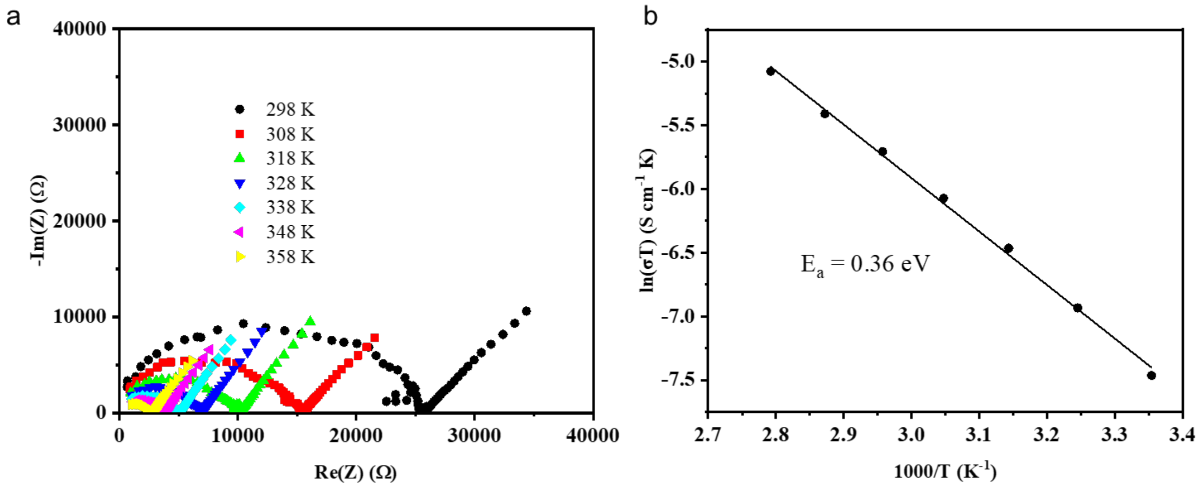

Figure S28. (a) Nyquist plots of Cu-TTBT-Mg at different temperature. (b) Temperature-dependent conductivity plots for Cu-TTBT-Mg in the range of 25 °C to 85 °C with 10 °C intervals.

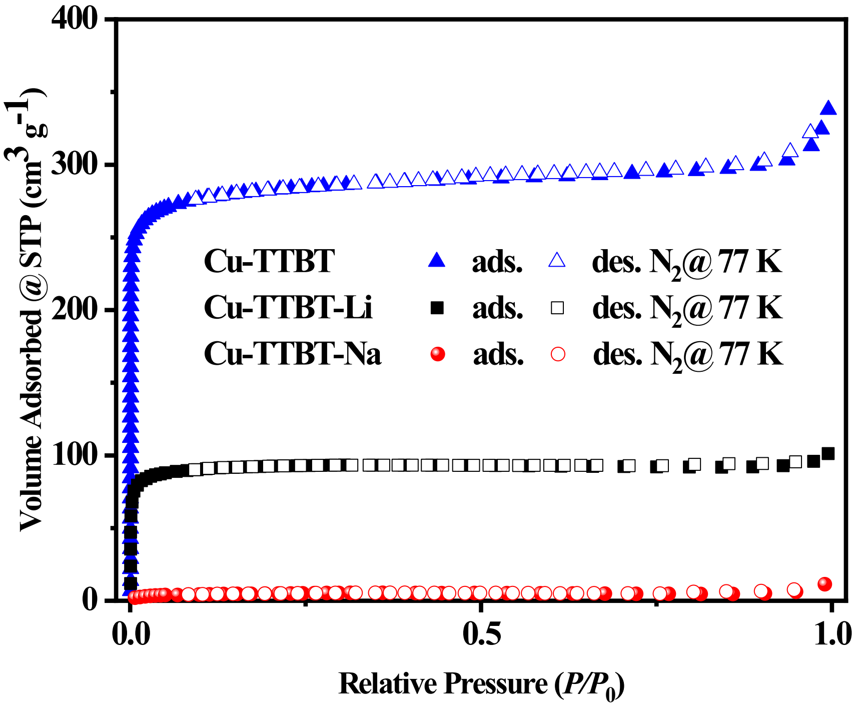

Figure S29. Nitrogen sorption isotherms of Cu-TTBT, Cu-TTBT-Li, and Cu-TTBT-Na recorded at 77 K.

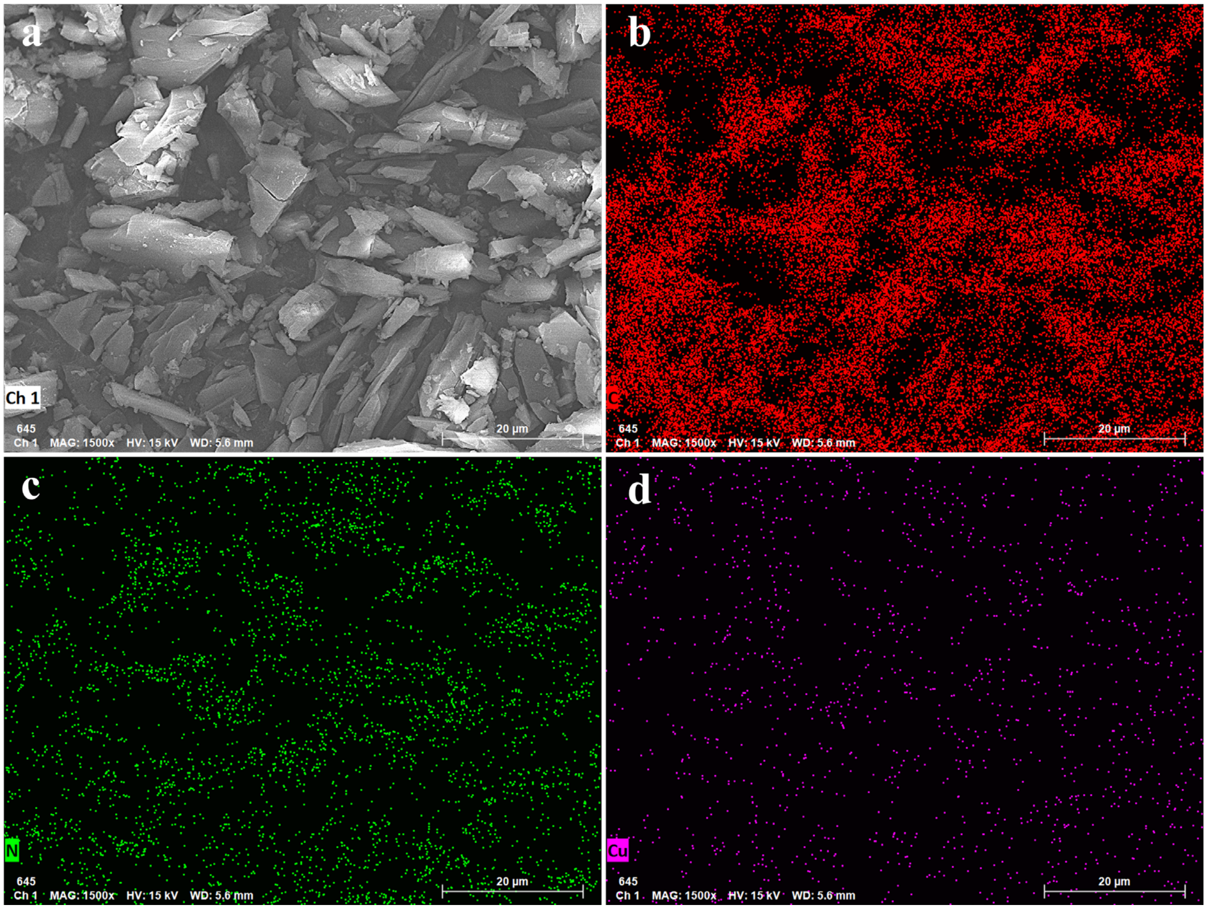

Figure S30.The energy-dispersive X-ray spectroscopy (EDX) mapping of Cu-TTBT-Li. (a) SEM image of Cu-TTBT-Li. (b) The corresponding EDX element mappings of C. (c) The corresponding EDX element mappings of N. (d)The corresponding EDX element mappings of Cu.

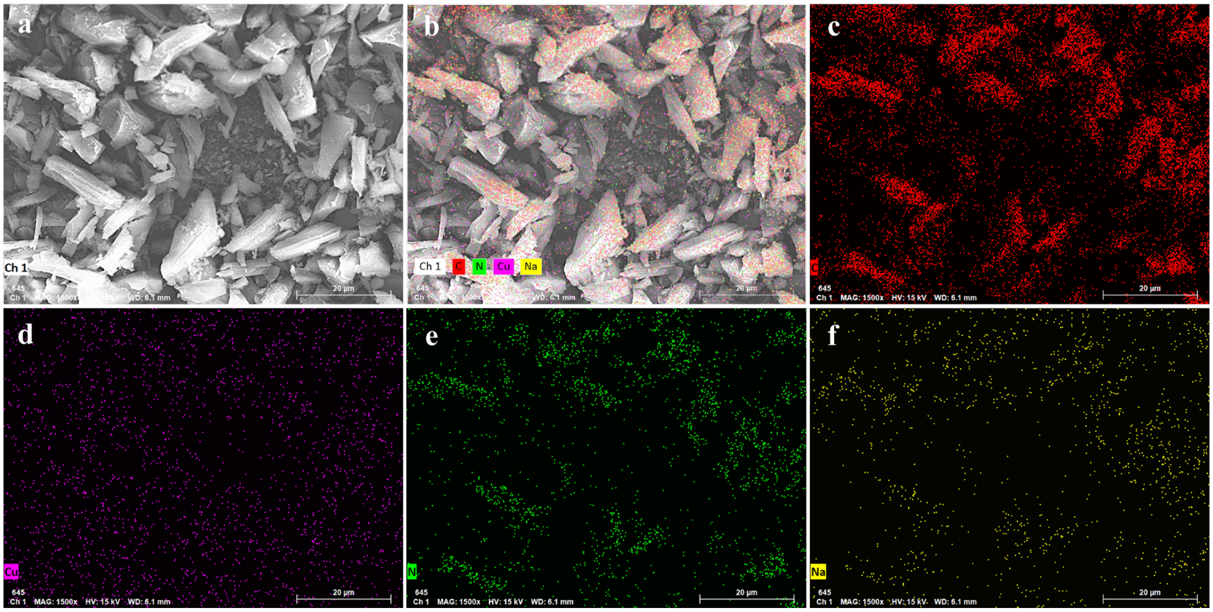

Figure S31. The energy-dispersive X-ray spectroscopy (EDX) mapping of Cu-TTBT-Na. (a) SEM image of Cu-TTBT-Na. (b) The corresponding EDX element mappings of C, Cu, N, and Na. (c) The corresponding EDX element mappings of C. (d) The corresponding EDX element mappings of Cu. (e)The corresponding EDX element mappings of N. (f) The corresponding EDX element mappings of Na.

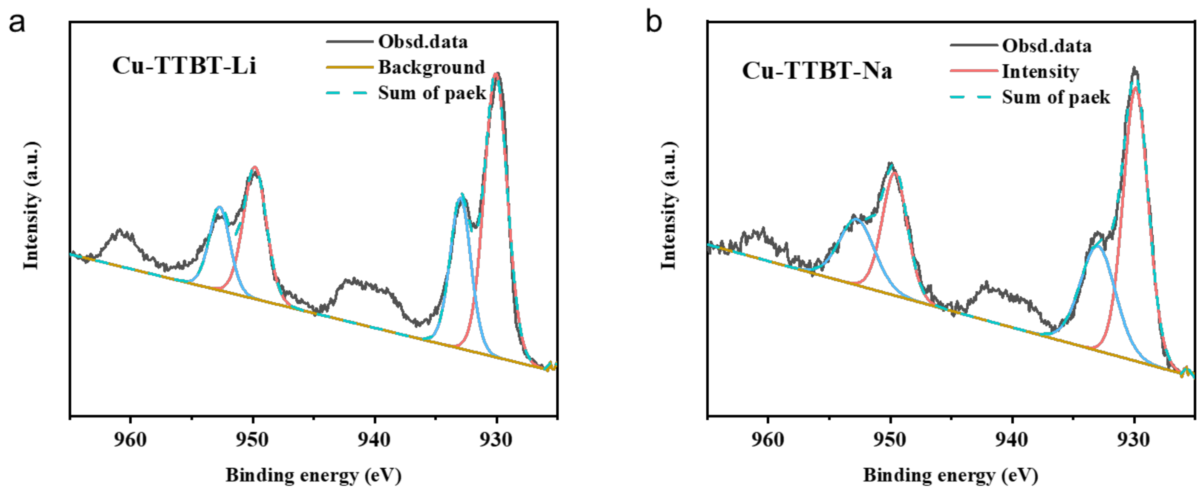

Figure S32. (a) XPS spectra of Cu-TTBT-Li. (b) XPS spectra of Cu-TTBT-Na.

Table S3. The ratio of Cu(I) and Cu(II) in Cu-TTBT, Cu-TTBT-Li, and Cu-TTBT-Na.

Materials	Cu(II)	Cu(I)
Cu-TTBT	0.3	0.7
Cu-TTBT-Li	0.32	0.68
Cu-TTBT-Na	0.35	0.65

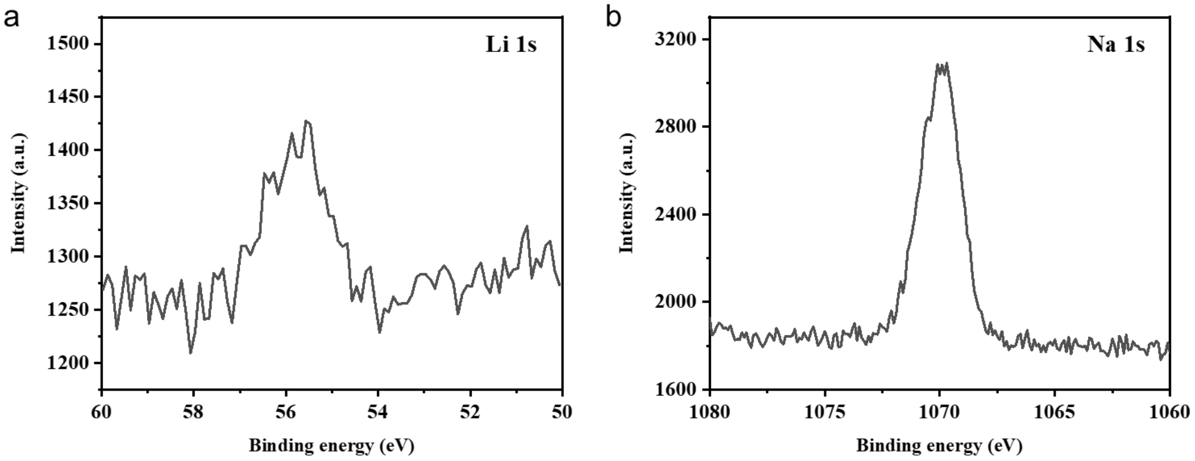

Figure S33. (a) XPS spectra of Cu-TTBT-Li. (b) XPS spectra of Cu-TTBT-Na.

Table S4. The result of ICP-MS for Cu-TTBT-Li.

Element	Concentration (μg/L)	Molar ratio
Li	183.86	1
Cu	16076.4	9

Table S5. The result of ICP-MS for Cu-TTBT-Na.

Element	Concentration (μg/L)	Molar ratio
Na	864	1.5
Cu	12650	0.85

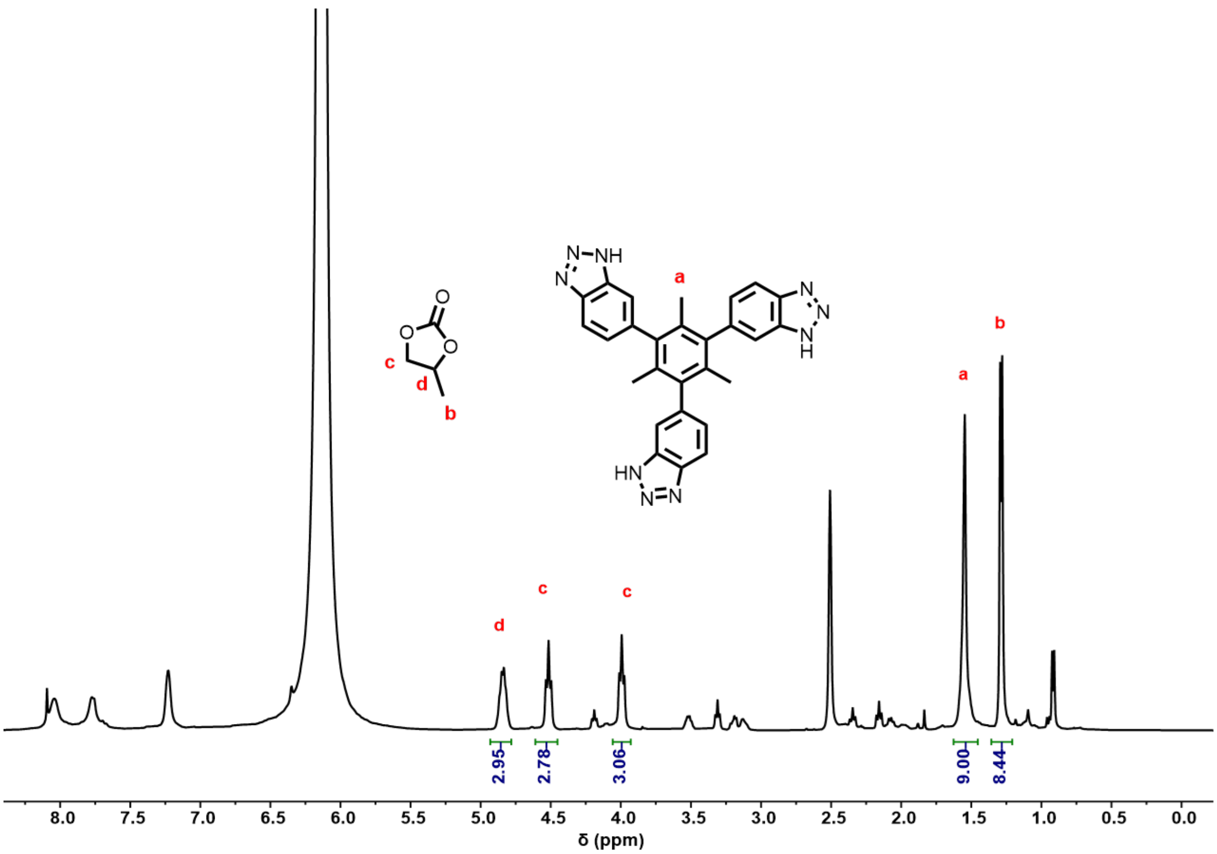

Figure S34. ^{1}H NMR spectrum of digested Cu-TTBT-Li.

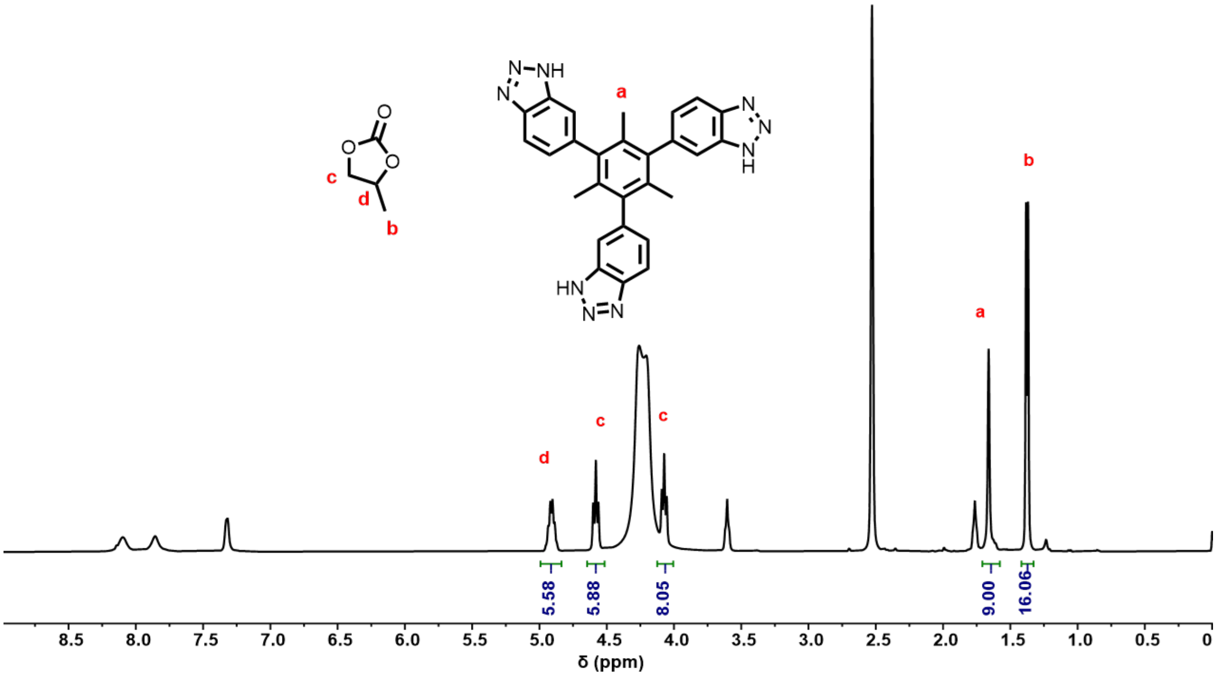

Figure S35. ^{1}H NMR spectrum of digested Cu-TTBT-Na.

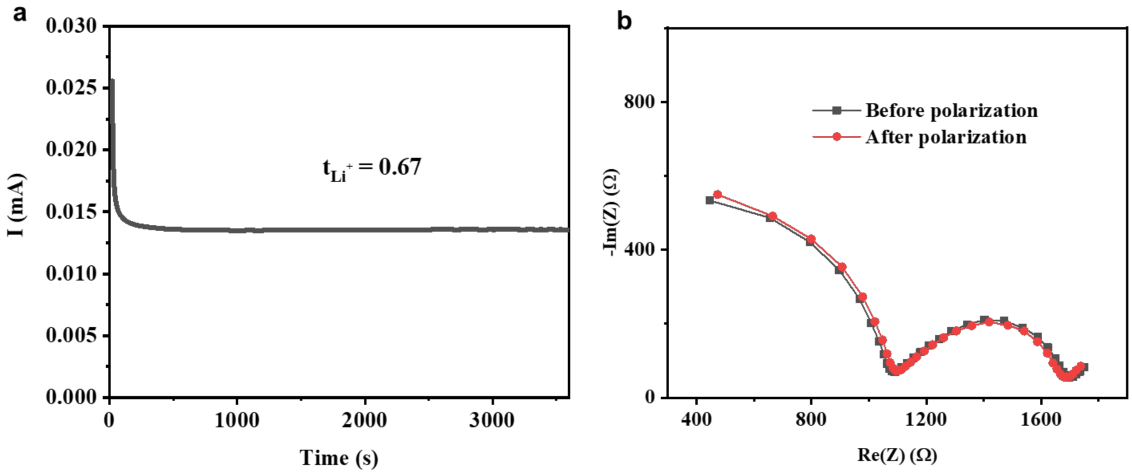

Figure S36. (a) Chronoamperogram during the polarization of a Li|Cu-TTBT-Li|Li cell at 25 °C with an applied potential of 50 mV. (b) Nyquist plots of the Li|Cu-TTBT-Li|Li cell at 25 °C before and after the polarization experiment.

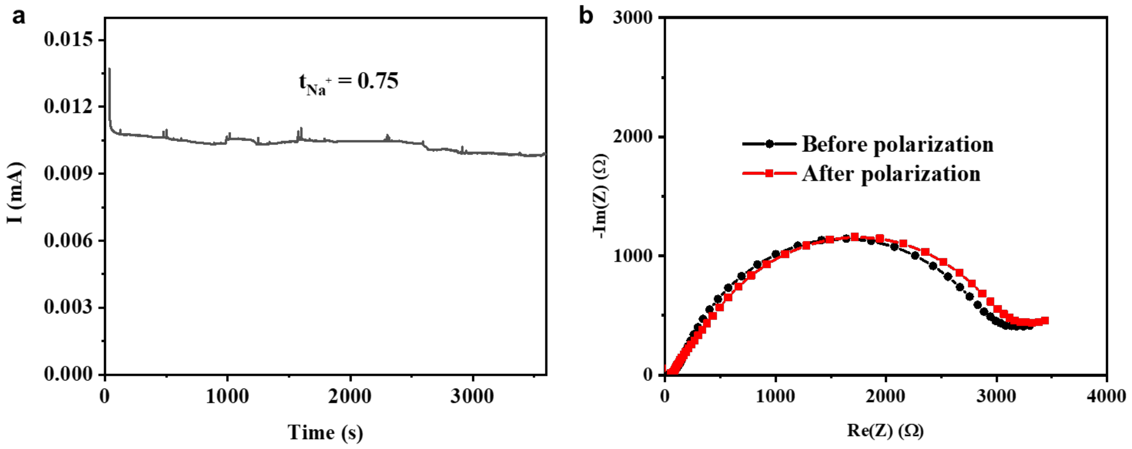

Figure S37. (a) Chronoamperogram during the polarization of a Na|Cu-TTBT-Na|Na cell at 25 °C with an applied potential of 50 mV. (b) Nyquist plots of the Na|Cu-TTBT-Na|Na cell at 25 °C before and after the polarization experiment.

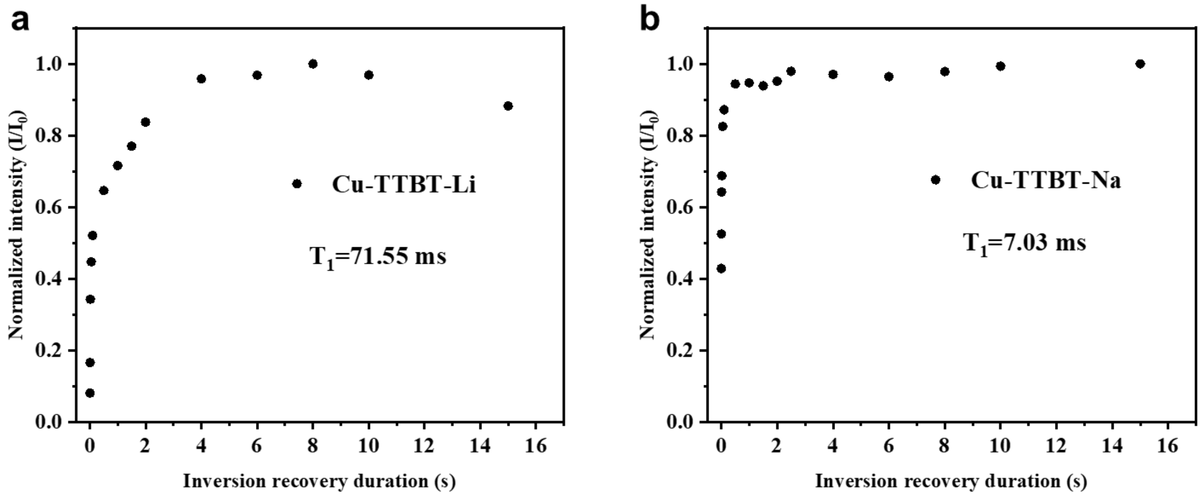

Figure S38. (a) Saturation recovery plot from ^{7}Li solid-state NMR spectra of Cu-TTBT-Li. (b) Saturation recovery plot from ^{23}Na solid-state NMR spectra of Cu-TTBT-Na.

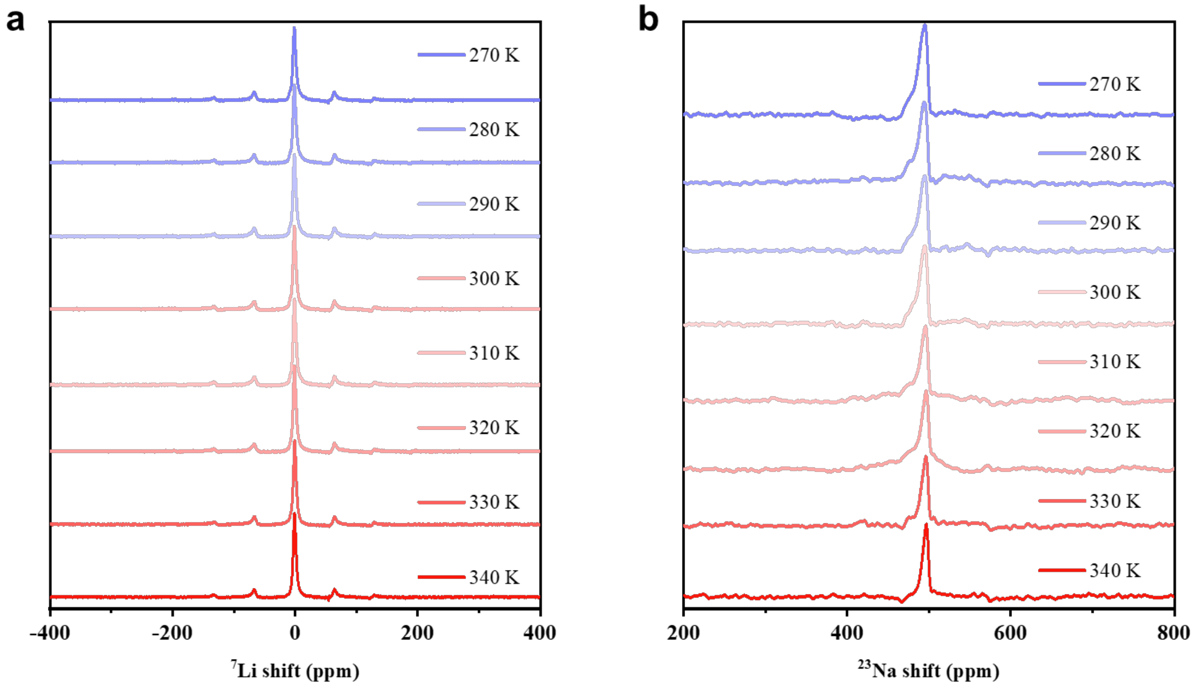

Figure S39. (a) Temperature-dependent evolution of the ^{7}Li line shape of Cu-TTBT-Li from 270 K to 340 K. (b) Temperature-dependent evolution of the ^{23}Na line shape of Cu-TTBT-Na from 270 K to 340 K.

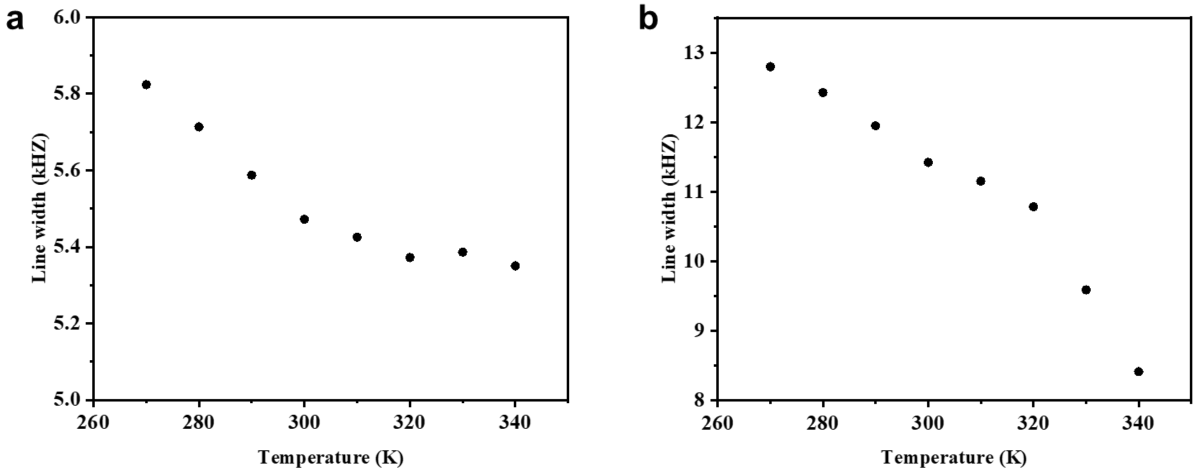

Figure S40. (a) Motional narrowing of the line width of the central transition of the ^{7}Li solid-state NMR of Cu-TTBT-Li. (b) Motional narrowing of the line width of the central transition of the ^{23}Na solid-state NMR of Cu-TTBT-Na.

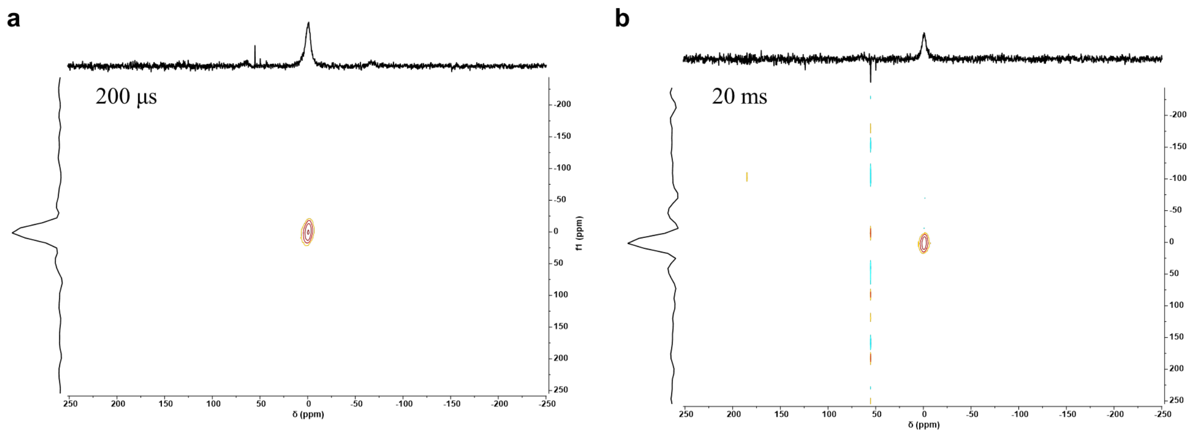

Figure S41. (a, b) ^{7}Li 1D solid-state NMR and ^{7}Li-^{7}Li 2D-EXSY of Cu-TTBT-Li with a mixing time of 200 μs and 20 ms.

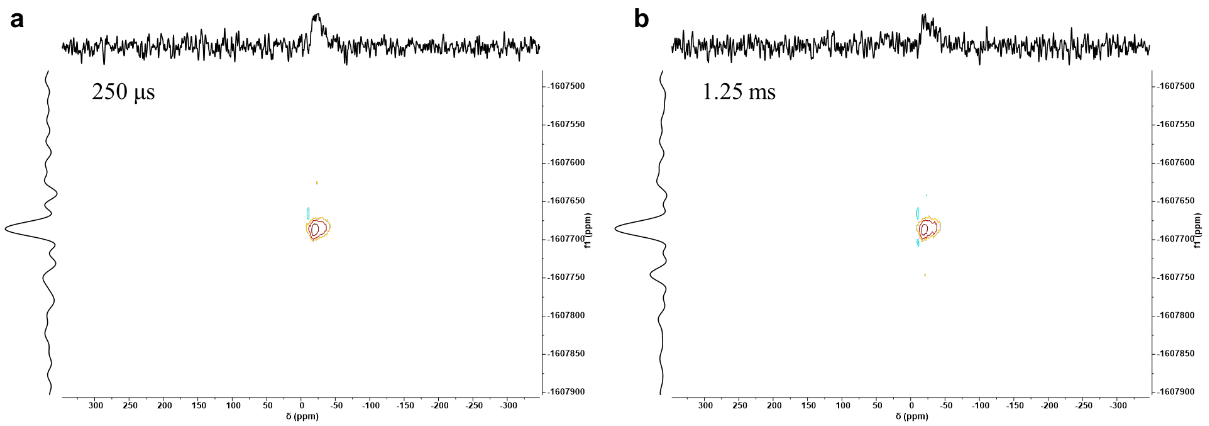

Figure S42. (a, b) ^{23}Na 1D solid-state NMR and ^{23}Na-^{23}Na 2D-EXSY of Cu-TTBT-Na with a mixing time of 250 μs and 1.25 ms.

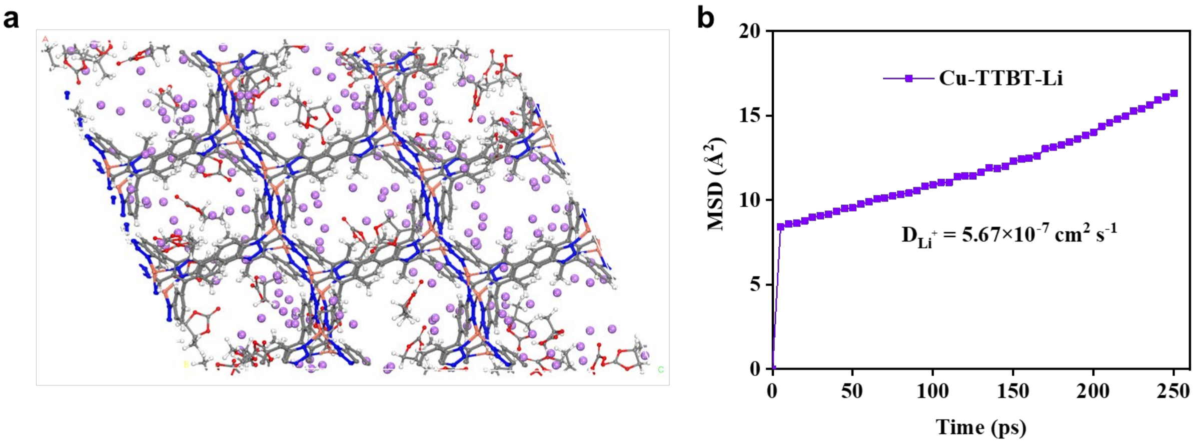

Figure S43. (a) Side view of 3D snapshots obtained by MD simulations for the Li^+ solvation structure in the Cu-TTBT-Li. (b) MSD of Li^+ in Cu-TTBT-Li as a function of the simulation time. H atoms were omitted for clarity; color code for structure: Cu, orange; C, grey; N, blue; Na, purple.

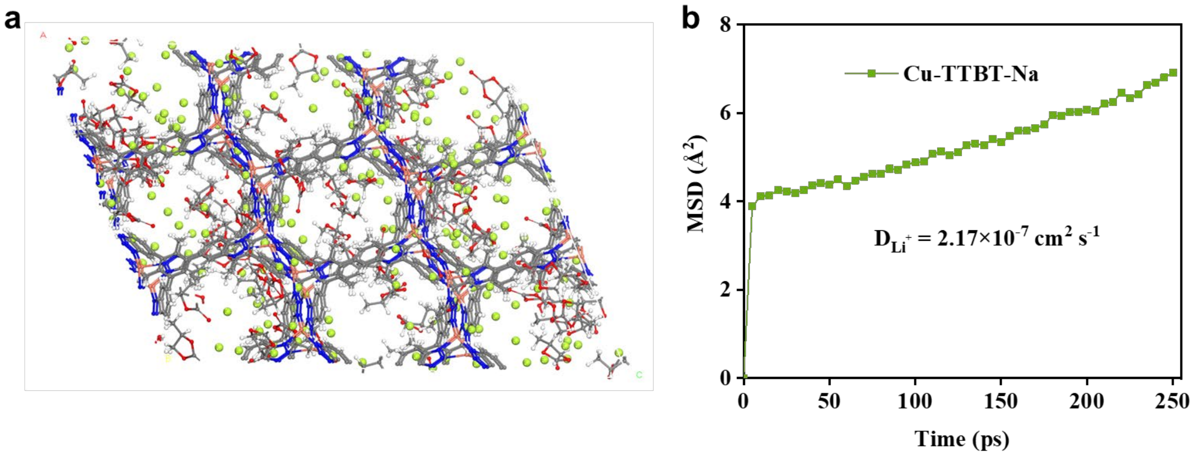

Figure S44. (a) Side view of 3D snapshots obtained by MD simulations for the Na^+ solvation structure in the Cu-TTBT-Na. (b) MSD of Na^+ in Cu-TTBT-Na as a function of the simulation time. H atoms were omitted for clarity; color code for structure: Cu, orange; C, grey; N, blue; Li, green.

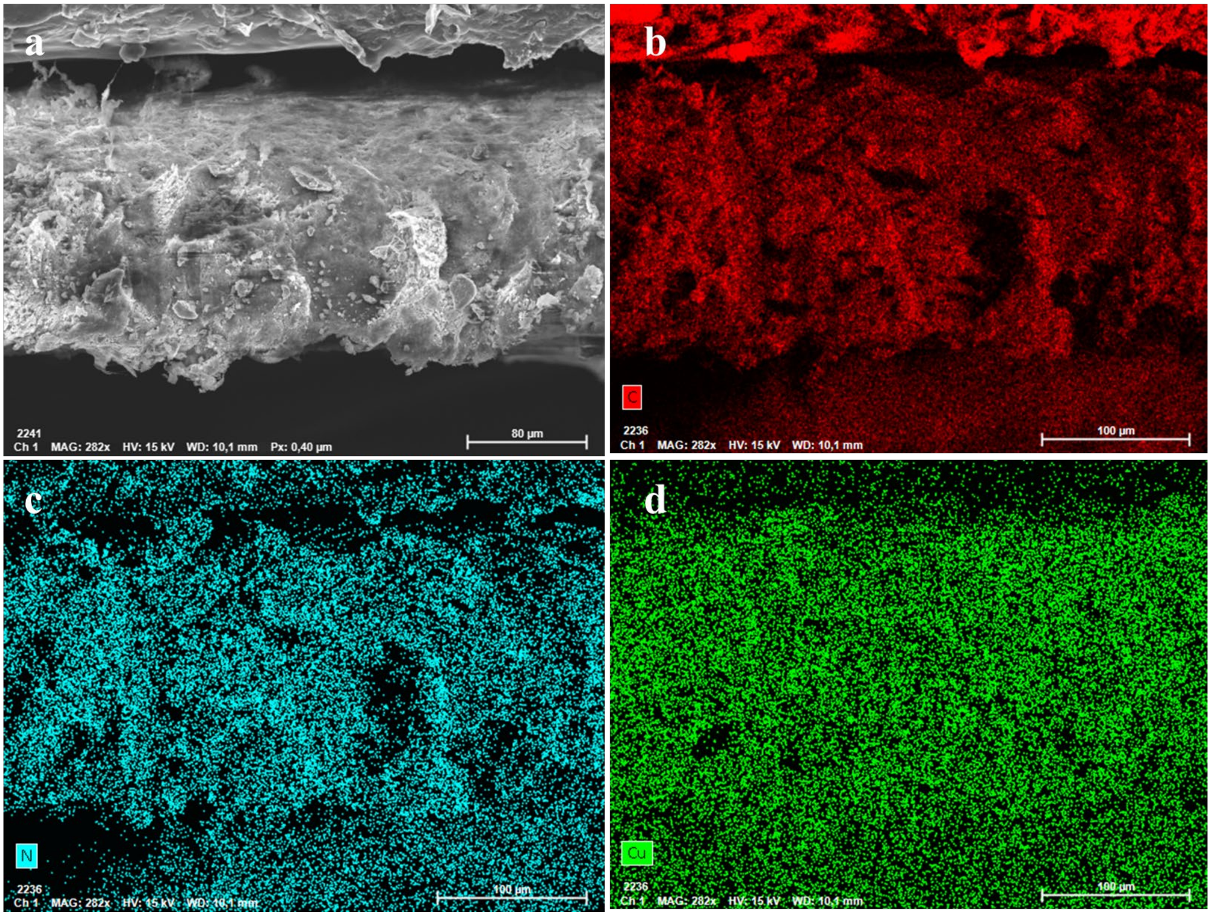

Figure S45. The cross-sectional EDX mapping images of Cu-TTBT-Li. (a) SEM images, (b) C, (c) N, (d) Cu element.

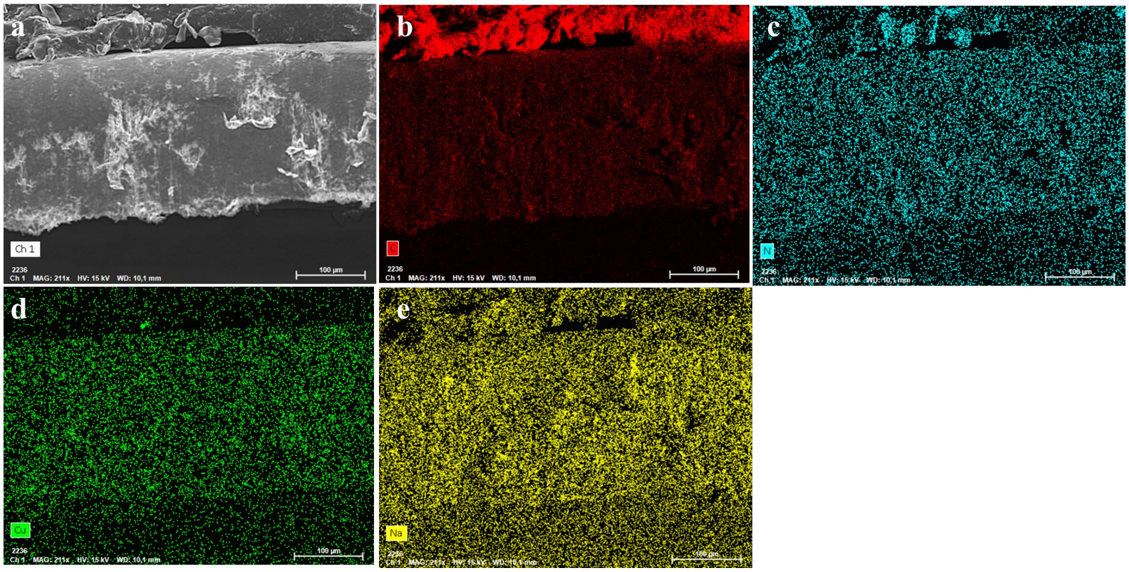

Figure S46. The cross-sectional EDX mapping images of Cu-TTBT-Na. (a) SEM images, (b) C, (c) N, (d) Cu, (e) Na element.

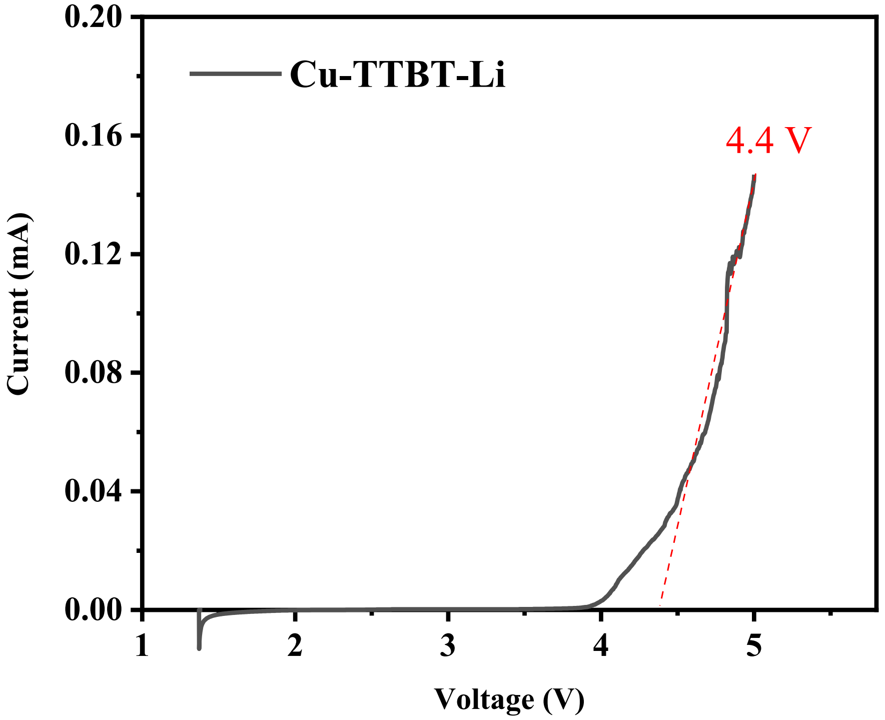

Figure S47. LSV curve of Li|Cu-TTBT-Li|stainless steel asymmetric cells obtained at a scan speed of 0.5 mV s^{-1}.

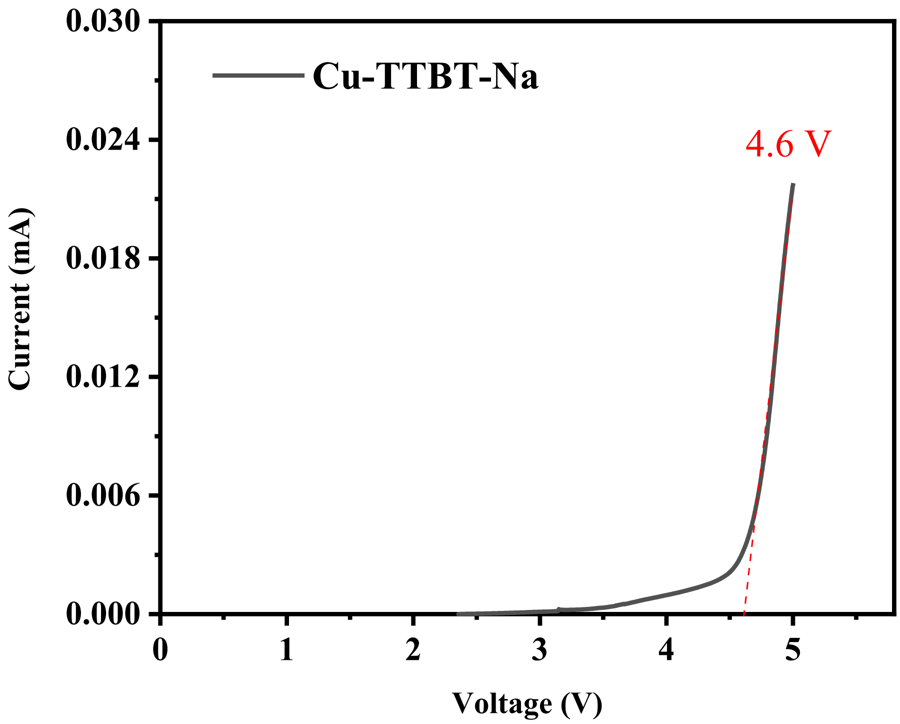

Figure S48. LSV curve of Na|Cu-TTBT-Na|stainless steel asymmetric cells obtained at a scan speed of 0.5 mV s^{-1}.

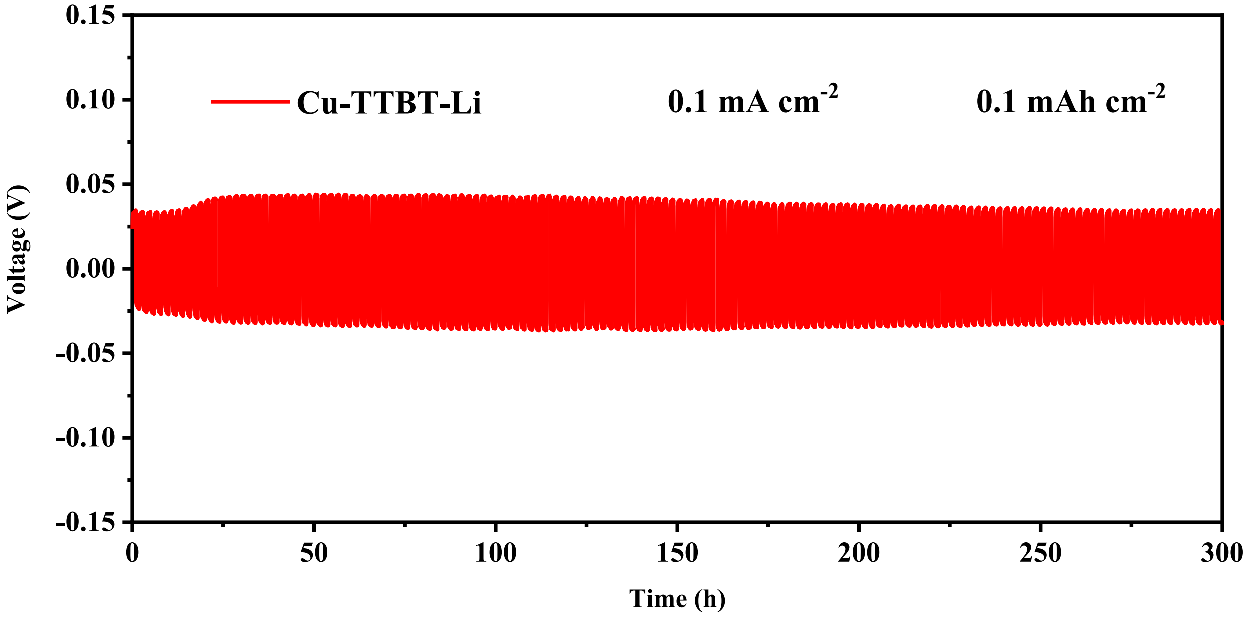

Figure S49. Voltage profiles of Li||Li symmetrical cells with Cu-TTBT-Li at 0.1 mA cm^{-2}.

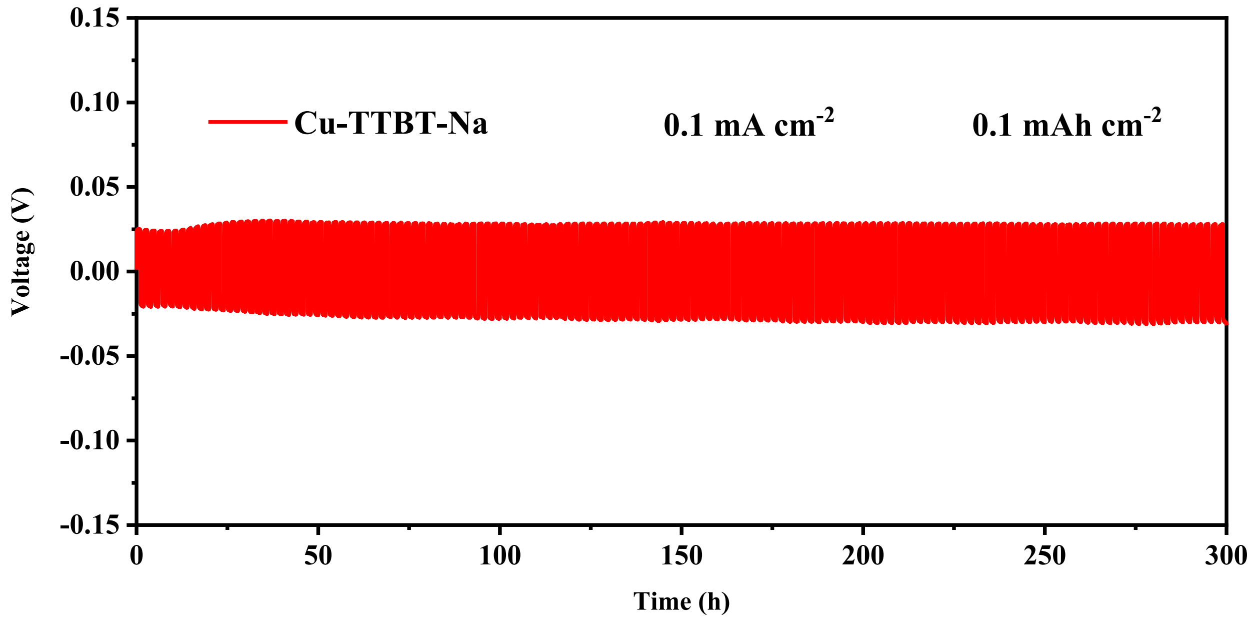

Figure S50. Voltage profiles of Na||Na symmetrical cells with Cu-TTBT-Na at 0.1 mA cm^{-2}.

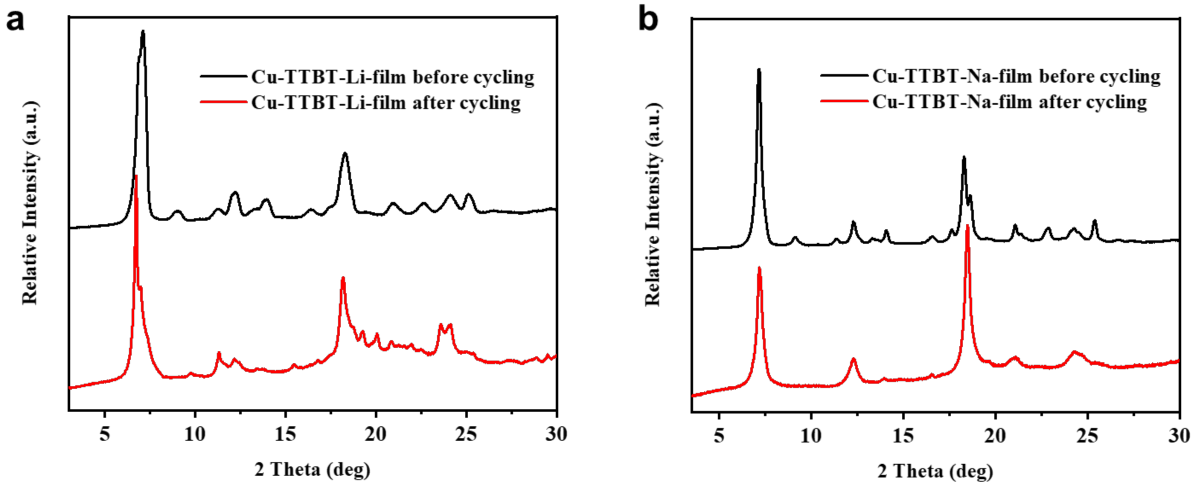

Figure S51. (a) PXRD patterns of Cu-TTBT-Li film before and after Li plating/stripping cycles. (b) PXRD patterns of Cu-TTBT-Na film before and after Na plating/stripping cycles.

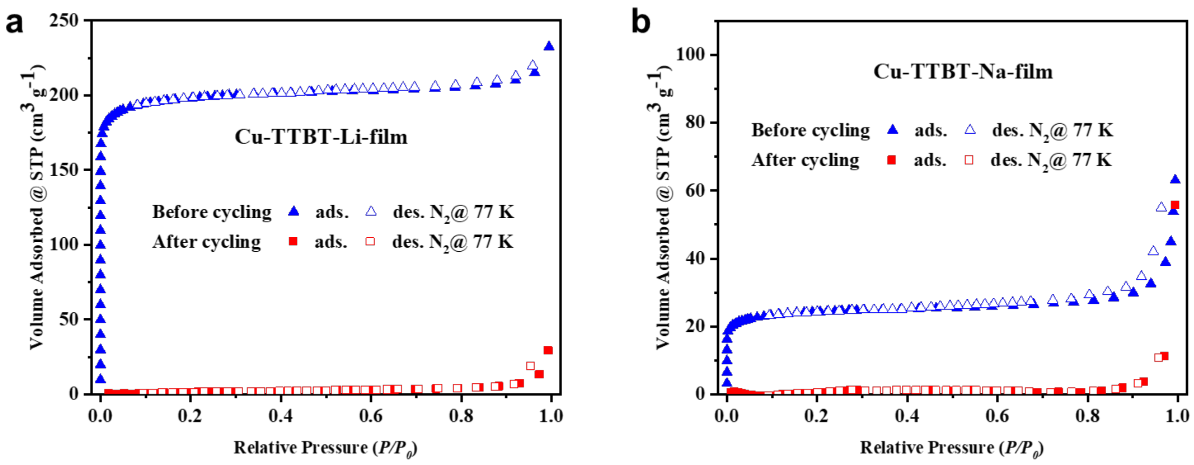

Figure S52. (a) Comparison of N_2 sorption isotherms at 77 K for Cu-TTBT-Li film before and after Li plating/stripping cycles. (b) Comparison of N_2 sorption isotherms at 77 K for Cu-TTBT-Na film before and after Na plating/stripping cycles.

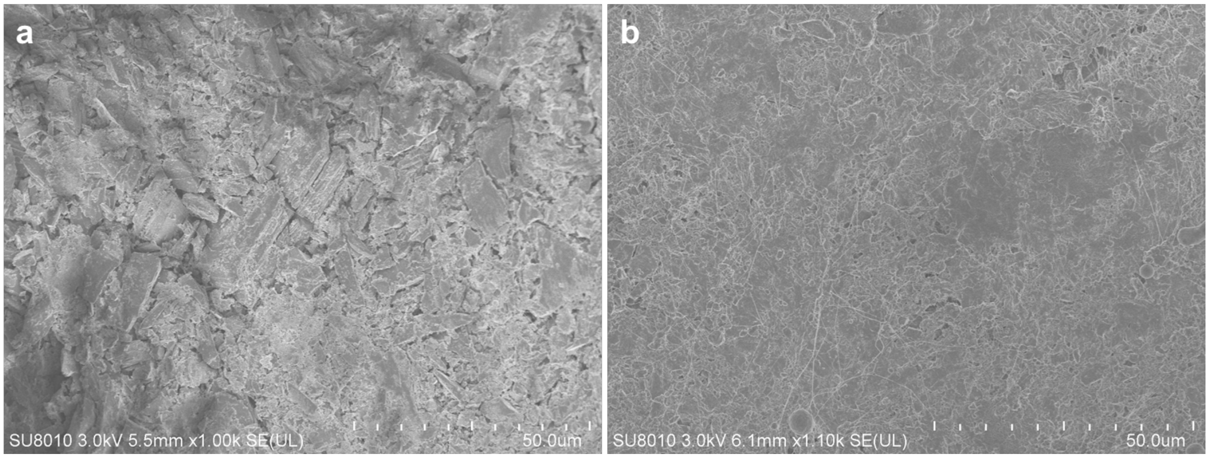

Figure S53. The SEM images of Cu-TTBT-Li (a) before and (b) after Li plating/stripping cycles.

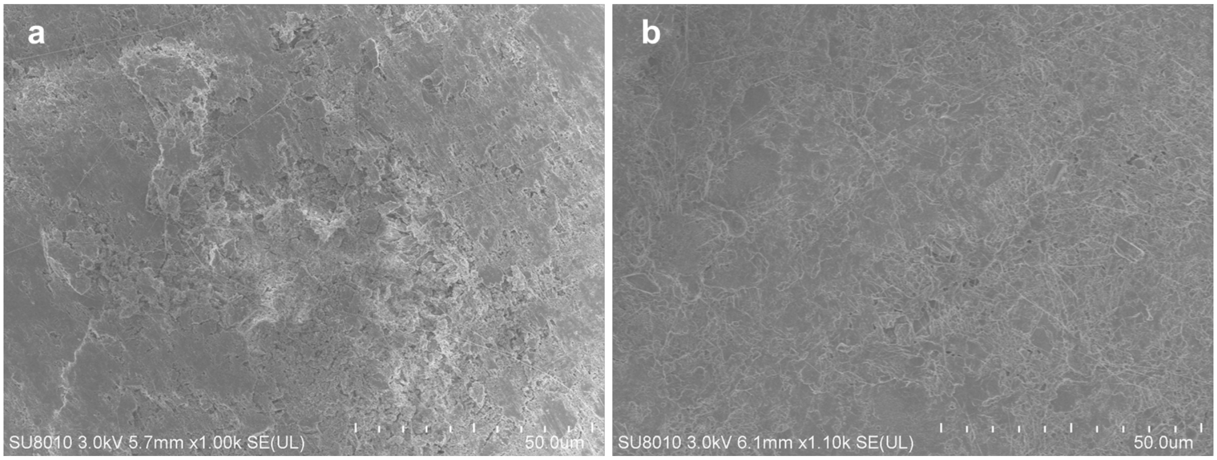

Figure S54. The SEM images of Cu-TTBT-Na (a) before and (b) after Na plating/stripping cycles.

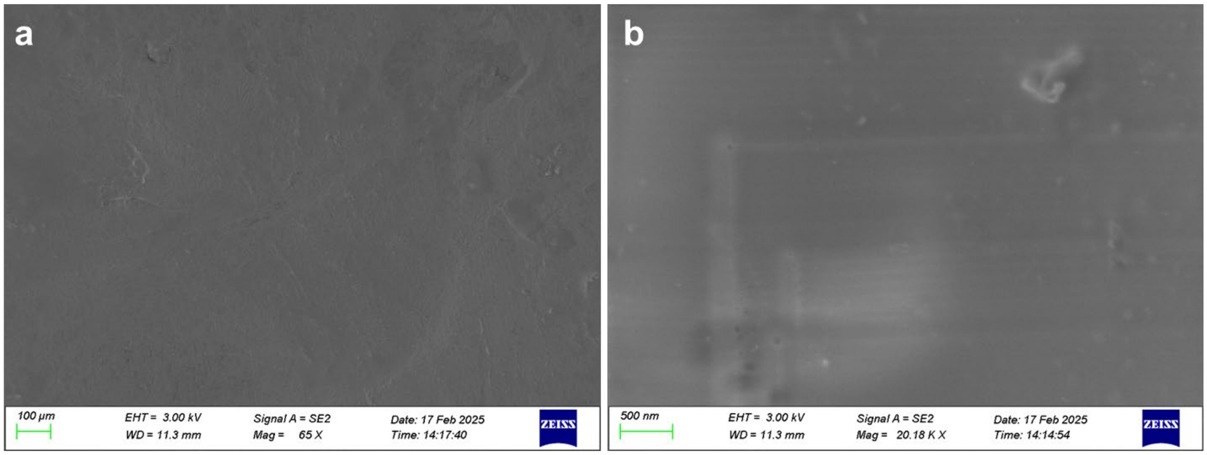

Figure S55. (a-b) SEM images of Li deposition on the Li metal electrode with different magnifications.

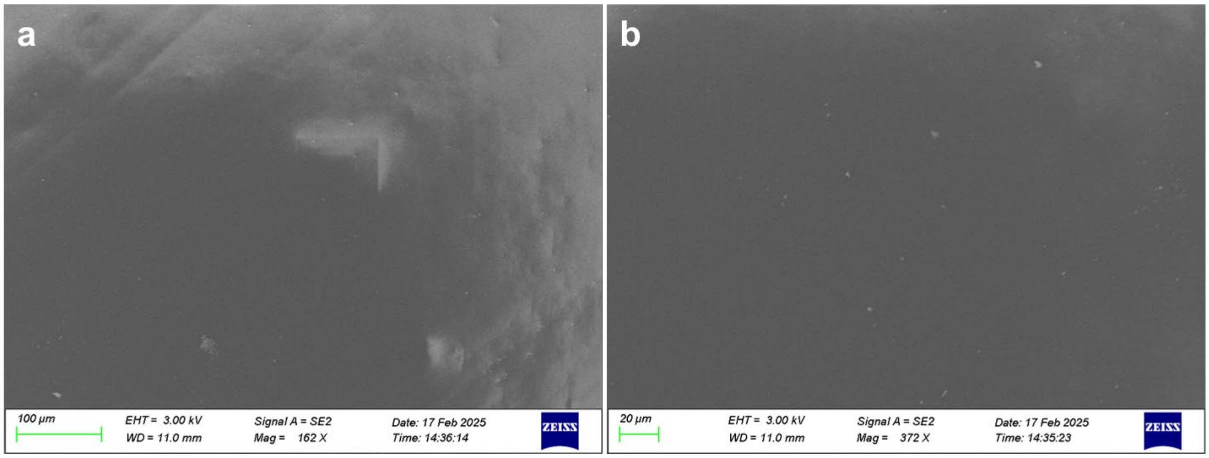

Figure S56. (a-b) SEM images of Na deposition on the Na metal electrode with different magnifications.

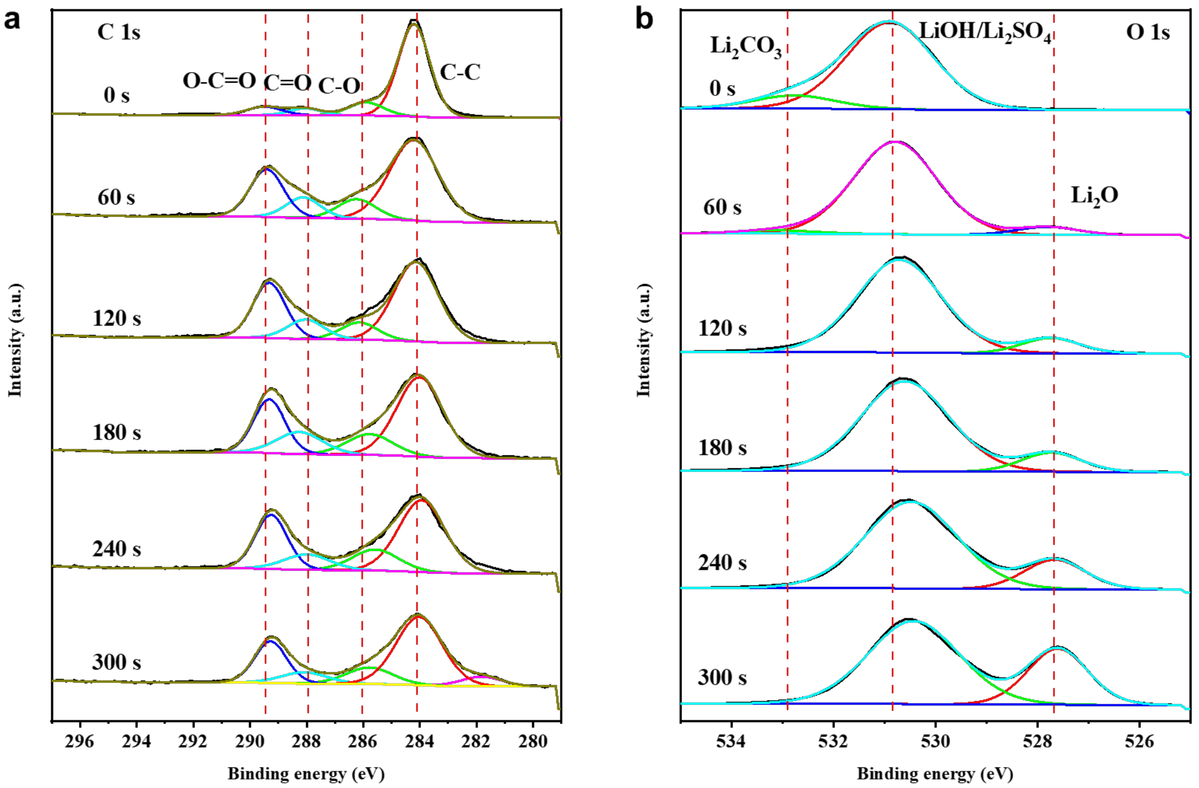

Figure S57. (a) XPS spectra for C 1s with different Ar^+ sputtering times on Li foils in the Cu-TTBT-Li. (b) XPS spectra for O 1s with different Ar^+ sputtering times on Li foils in the Cu-TTBT-Li.

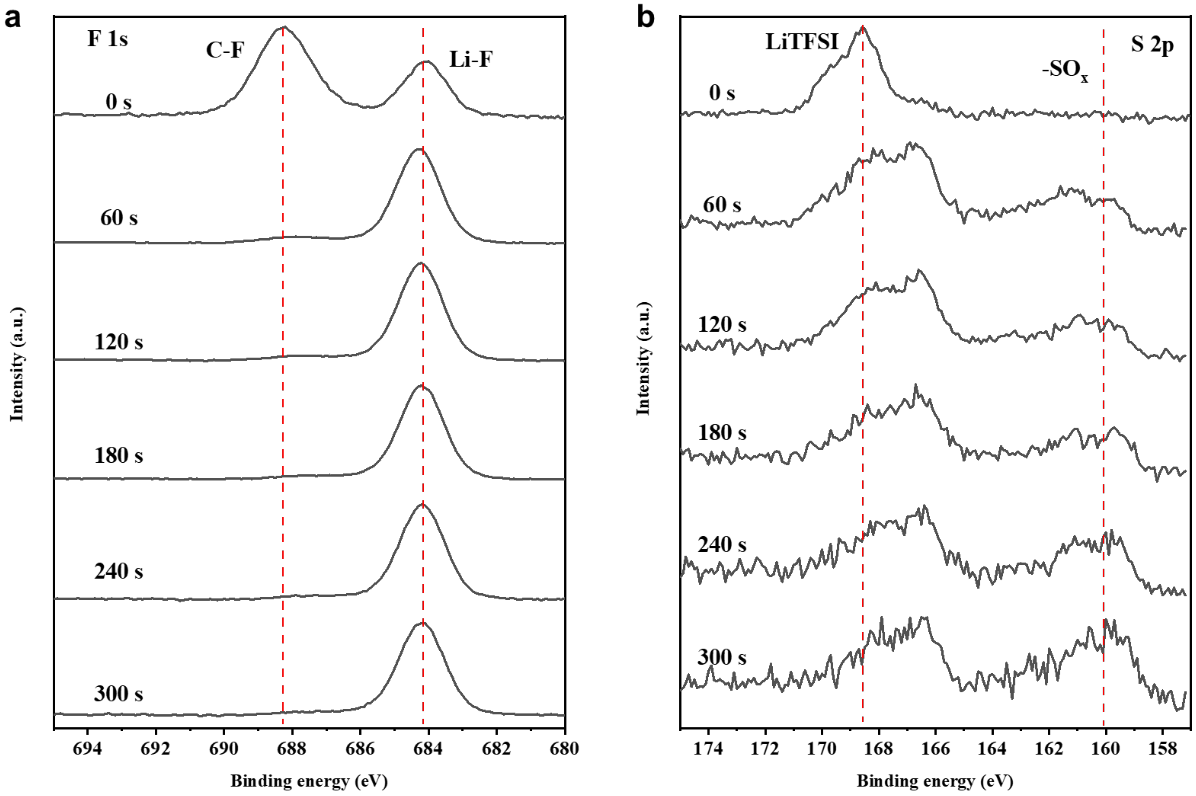

Figure S58. (a) XPS spectra for F 1s with different Ar^+ sputtering times on Li foils in the Cu-TTBT-Li. (b) XPS spectra for S 2p with different Ar^+ sputtering times on Li foils in the Cu-TTBT-Li.

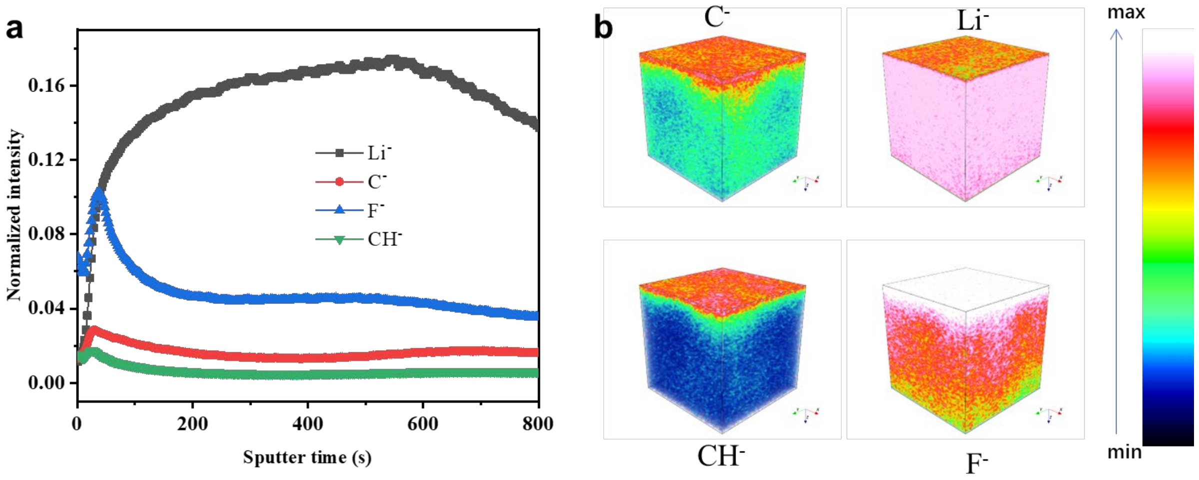

Figure S59. (a) TOF-SIMS depth profiling was conducted for 800 seconds of etching. (b) 3D reconstruction images for C^-, Li^-, F^-, and CH^- fragments with different depths.

9. Li metal battery and Na metal battery

9.1 The synthesis of MOF-based SSE membrane

400 mg Cu-TTBT-Li, 100 mg PTFE were mixed with an ethanol solution to form membrane. The ethanol was subsequently removed under vacuum. The obtained membrane was immersed in 1 M LiTFSI/PC solution for 24 h for activation. Finally, The Li^+ exchanged membrane was immersed in anhydrous propylene carbonate (5 mL × 3) over three days.

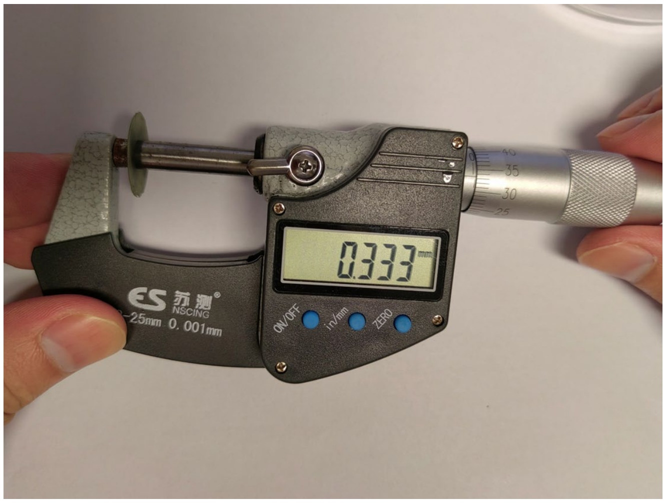

Figure S60. Optical image of the thickness of SSEs membrane.

9.2 Galvanostatic cycling measurements on coin cell battery

Lithium metal battery: the cathode slurry was prepared by mixing $LiFePO_4$, ECP-600JDV and PVDF with a mass ratio of 8:1:1 in *N*-Methylpyrrolidone (NMP). The cathode slurry was cast onto aluminum foil within a loading capacity of 2-4 mg and dried at 65 °C for 24 h. The cathode films were punched into round pellets with a diameter of 12 mm. Solid state lithium metal batteries were assembled with a lithium metal anode, an SSEs membrane, and a $LiFePO_4$ cathode in an Ar-filled glove box and sealed in a 2032-coin cell for testing. The charging/discharging curves were collected with a Land CT2001A battery testing system.

Sodium metal battery: the cathode slurry was prepared by mixing $Na_3V_2(PO_4)_3$, ECP-600JDV and PVDF with a mass ratio of 8:1:1 in NMP. The cathode slurry was cast onto aluminum foil within a loading capacity of 2-4 mg and dried at 65 °C for 24 h. The cathode films were punched into round pellets with a diameter of 12 mm. Solid state sodium metal batteries were assembled with a sodium metal anode, an SSEs membrane, and a $Na_3V_2(PO_4)_3$ cathode in an Ar-filled glove box and sealed in a 2032-coin cell for testing. The charging/discharging curves were collected with a Land CT2001A battery testing system.

Galvanostatic cycling measurements were carried out by cycling the battery between 2.8 V and 3.8 V.

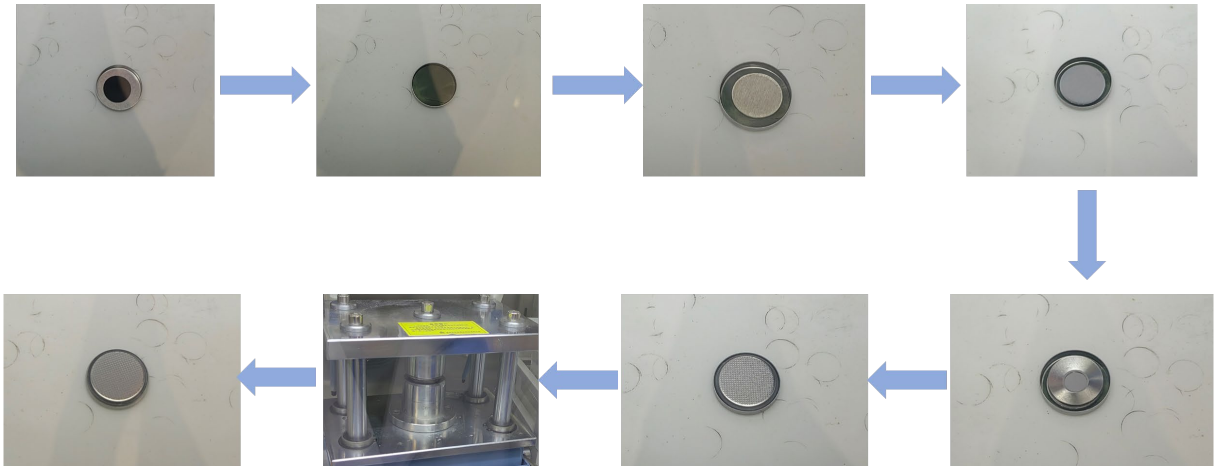

Figure S61. Schematic illustration for preparation lithium metal battery or sodium metal battery.

Table S6. The summary of benzotriazolate-based MOFs made by *de novo* synthesis.

MOF	Inorganic node	Organic linker	Ref.
MFU-4	Zn	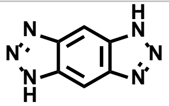	8
MFU-4l	Zn	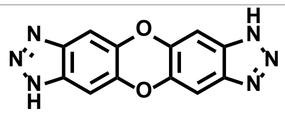	8
CFA-1	Zn	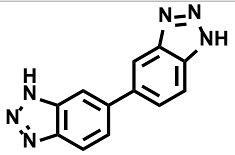	9
CFA-7	Zn	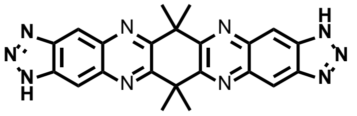	10
CFA-8	Cu	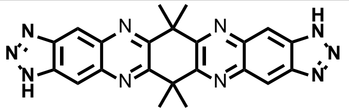	11
CFA-18	Mn	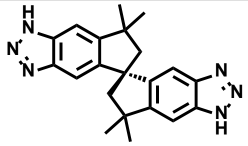	12
Mn_2Cl_2(BTDD)	Mn	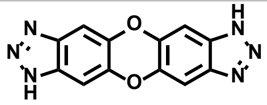	13
Co_2Cl_2(BTDD)	Co	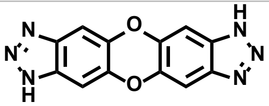	13
Ni_2Cl_2(BTDD)	Ni	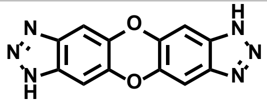	13
MIT-20	Cu	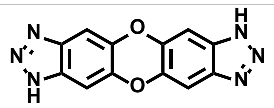	14
Mn_2Cl_2BBTA	Mn	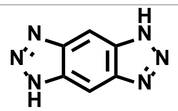	15
MAF-X27	Co	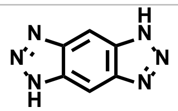	16

Ni_2Cl_2BBTA	Ni	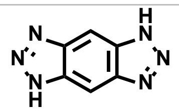	17
Cu_2Cl_2BBTA	Cu	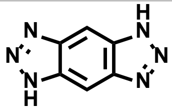	17
NU-2100	Cu	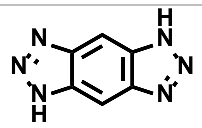	18
$V_2Cl_{2.8}$(btdd)	V	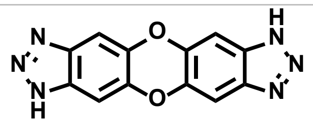	19
Fe_2Cl_2(btdd)	Fe	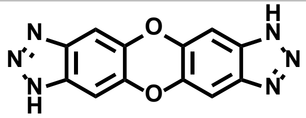	20
Fe_2Cl_2(bbta)	Fe	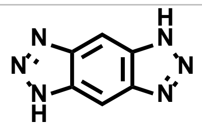	20
**Cu-TTBT**	**Cu**	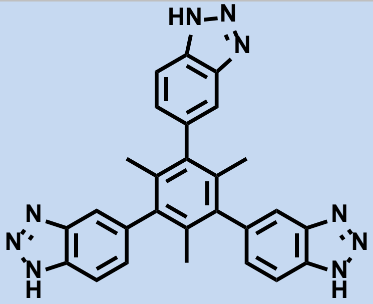	**This work**

Table S7. Comparison of Li-ion conducting performances of materials (RT: room temperature).

**Materials**	**Description**	**σ (S cm^{-1})**	**T**	**E_a (eV)**	**t_{Li^+}**	**Ref.**
MIT-20-LiCl	$Li[Cu_2Cl_3BTDD]\cdot 10PC$	1.3×10^{-5}	25 °C	0.32	0.66	14
MIT-20-LiBr	$Li_{0.8}[Cu_2Cl_2Br_{0.8}BTDD]\cdot 10PC$	4.4×10^{-5}	25 °C	0.29	/	14
$Cu_4(ttpm)_2$-LiCl	$Cu_4(ttpm)_2(CuCl_2)_{0.6}(LiCl)_{1.8}\cdot 19PC$	2.4×10^{-5}	25 °C	0.34	0.69	21
$Cu_4(ttpm)_2$-LiBr	$Cu_4(ttpm)_2(CuCl_2)_{0.6}(LiBr)_{1.8}\cdot 20PC$	3.2×10^{-5}	25 °C	0.30	0.42	21
$Cu_4(ttpm)_2$-LiI	$Cu_4(ttpm)_2(CuCl_2)_{0.6}(LiI)_{1.0}\cdot 20PC$	1.1×10^{-4}	25 °C	0.24	0.34	21
LiOiPr in Mg_2(dobdc)	$Mg_2(dobdc)\cdot 0.35LiOiPr\cdot 0.25LiBF_4\cdot EC\cdot DEC$	3.1×10^{-4}	27 °C	0.15	/	22
MOF-688	PVDF/PC	3.4×10^{-4}	20 °C	/	0.87	23
Al-Td-MOF-1	$Li[Al(C_6H_4O_2)_2]$	5.7×10^{-5}	RT	/	/	24
ICOF-2	PVDF/PC	3.05×10^{-5}	25 °C	0.24	0.8	25
EC/DEC@Ge-COF-1	24 wt % EC/DEC	1.6×10^{-5}	30 °C	0.24	/	26
H-Li-ImCOF	20 wt % PC	5.3×10^{-3}	RT	0.12	0.88	27
CH_3-Li-ImCOF	20 wt % PC	8.0×10^{-5}	RT	0.27	0.93	27
CF_3-Li-ImCOF	20 wt % PC	7.2×10^{-3}	RT	0.10	0.81	27
Li^+@TPB-BMTP-COF	$LiClO_4$	6.04×10^{-6}	40 °C	0.87	/	28
TpPa-SO_3Li	PVDF/PC	2.7×10^{-5}	RT	0.18	0.9	29
LiCON-1	20 wt % EC	2.13×10^{-7}	20 °C	0.25	0.86	30
LiCON-2	20 wt % EC	4.36×10^{-6}	20 °C	0.22	0.83	30
LiCON-3	20 wt % EC	3.21×10^{-5}	20 °C	0.13	0.83	30
COF-$SO_3^-$$Li^+$	/	2.58×10^{-5}	25 °C	0.18	0.89	31
COF-$CO_2^-$$Li^+$	/	0.32×10^{-5}	25 °C	0.21	0.86	31
COF-$TFSI^-$$Li^+$	/	8.26×10^{-5}	25 °C	0.14	0.91	31
**Cu-TTBT-Li**	**$Li_{0.23}Cu_2TTBT\cdot 3PC$**	**1.83×10^{-4}**	**25 °C**	**0.30**	**0.67**	**This work**

Table S8. Comparison of Na-ion conducting performances of materials (RT: room temperature).

Materials	Description	σ (S cm-1)	*T*	E_a (eV)	t_{Li}^+	Ref.
MIT-20-Na	$Na[Cu_2Cl_2(SCN)BTDD]\cdot 9PC$	1.8×10^{-5}	25 °C	0.39	/	[14]
TPDBD-CNa-QSSE	20 wt.% NaTFSI in methanol	1.30×10^{-4}	25 °C	/	0.9	[32]
NaOOC-COF	10.0 μL, 1.0 M of $NaPF_6$/PC	2.68×10^{-4}	20 °C	0.24	0.9	[33]
aiCOF-COONa@Na	NaTFSI/PVDF-HFP	1.2×10^{-4}	30 °C	0.07	/	[34]
**Cu-TTBT-Na**	**$Na_{0.35}Cu_2TTBT\cdot 5.5PC$**	**1.1×10^{-4}**	25 °C	**0.22**	**0.75**	**This work**

10. References

(1) Cichocka, M. O.; Ångström, J.; Wang, B.; Zou, X.; Smeets, S., High-throughput continuous rotation electron diffraction data acquisitionviasoftware automation. *J. Appl. Crystallogr.* **2018,** *51* (6), 1652-1661.

(2) Kabsch, W., Xds. *Acta Crystallogr. D Biol. Crystallogr.* **2010,** *66* (2), 125-132.

(3) Palatinus, L.; Brázda, P.; Jelínek, M.; Hrdá, J.; Steciuk, G.; Klementová, M., Specifics of the data processing of precession electron diffraction tomography data and their implementation in the program PETS2.0. *Acta Crystallogr. B Struct. Sci. Cryst. Eng. Mater.* **2019,** *75* (4), 512-522.

(4) Sheldrick, G. M., SHELXT– Integrated space-group and crystal-structure determination. *Acta Crystallogr. A: Found. Adv.* **2015,** *71* (1), 3-8.

(5) Sheldrick, G. M., Crystal structure refinement withSHELXL. *Acta Crystallogr.Sect. C Struct. Chem.* **2015,** *71* (1), 3-8.

(6) Dolomanov, O. V.; Bourhis, L. J.; Gildea, R. J.; Howard, J. A. K.; Puschmann, H., OLEX2: a complete structure solution, refinement and analysis program. *J. Appl. Crystallogr.* **2009,** *42* (2), 339-341.

(7) Zhu, H.-L.; Chen, H.-Y.; Han, Y.-X.; Zhao, Z.-H.; Liao, P.-Q.; Chen, X.-M., A porous π–π stacking framework with dicopper(I) sites and adjacent proton relays for electroreduction of CO2 to C2+ products. *J. Am. Chem. Soc.* **2022,** *144* (29), 13319-13326.

(8) Denysenko, D.; Grzywa, M.; Tonigold, M.; Streppel, B.; Krkljus, I.; Hirscher, M.; Mugnaioli, E.; Kolb, U.; Hanss, J.; Volkmer, D., Elucidating gating effects for hydrogen sorption in MFU-4-type triazolate-based metal–organic frameworks featuring different pore sizes. *Chem. Eur. J.* **2011,** *17* (6), 1837-1848.

(9) Schmieder, P.; Denysenko, D.; Grzywa, M.; Baumgärtner, B.; Senkovska, I.; Kaskel, S.; Sastre, G.; van Wüllen, L.; Volkmer, D., CFA-1: the first chiral metal–organic framework containing Kuratowski-type secondary building units. *Dalton Trans.* **2013,** *42* (30), 10786-10797.

(10) Schmieder, P.; Grzywa, M.; Denysenko, D.; Hambach, M.; Volkmer, D., CFA-7: an interpenetrated metal–organic framework of the MFU-4 family. *Dalton Trans.* **2015,** *44* (29), 13060-13070.

(11) Schmieder, P.; Denysenko, D.; Grzywa, M.; Magdysyuk, O.; Volkmer, D., A structurally flexible triazolate-based metal–organic framework featuring coordinatively unsaturated copper(i) sites. *Dalton Trans.* **2016,** *45* (35), 13853-13862.

(12) Knippen, K.; Bredenkötter, B.; Kanschat, L.; Kraft, M.; Vermeyen, T.; Herrebout, W.; Sugimoto, K.; Bultinck, P.; Volkmer, D., CFA-18: a homochiral metal–organic framework (MOF) constructed from rigid enantiopure bistriazolate linker molecules. *Dalton Trans.* **2020,** *49* (44), 15758-15768.

(13) Rieth, A. J.; Tulchinsky, Y.; Dincă, M., High and reversible ammonia uptake in mesoporous azolate metal–organic frameworks with open Mn, Co, and Ni sites. *J. Am. Chem. Soc.* **2016,** *138* (30), 9401-9404.

(14) Park, S. S.; Tulchinsky, Y.; Dincă, M., Single-ion Li^+, Na^+, and Mg^{2+} solid electrolytes supported by a mesoporous anionic Cu–azolate metal–organic framework. *J. Am. Chem. Soc.* **2017,** *139* (38), 13260-13263.

(15) Liao, P. Q.; Li, X. Y.; Bai, J.; He, C. T.; Zhou, D. D.; Zhang, W. X.; Zhang, J. P.; Chen, X. M., Drastic enhancement of catalytic activity via post-oxidation of a porous MnII triazolate framework. *Chem. Eur. J.* **2014,** *20* (36), 11303-11307.

(16) Liao, P.-Q.; Chen, H.; Zhou, D.-D.; Liu, S.-Y.; He, C.-T.; Rui, Z.; Ji, H.; Zhang, J.-P.; Chen, X.-M., Monodentate hydroxide as a super strong yet reversible active site for CO2 capture from high-humidity flue gas. *Energy Environ. Sci.* **2015,** *8* (3), 1011-1016.

(17) Rieth, A. J.; Dincă, M., Controlled gas uptake in metal–organic frameworks with record ammonia sorption. *J. Am. Chem. Soc.* **2018,** *140* (9), 3461-3466.

(18) Sengupta, D.; Melix, P.; Bose, S.; Duncan, J.; Wang, X.; Mian, M. R.; Kirlikovali, K. O.; Joodaki, F.; Islamoglu, T.; Yildirim, T.; Snurr, R. Q.; Farha, O. K., Air-stable Cu(I) metal–organic framework for hydrogen storage. *J. Am. Chem. Soc.* **2023,** *145* (37), 20492-20502.

(19) Jaramillo, D. E.; Reed, D. A.; Jiang, H. Z. H.; Oktawiec, J.; Mara, M. W.; Forse, A. C.; Lussier, D. J.; Murphy, R. A.; Cunningham, M.; Colombo, V.; Shuh, D. K.; Reimer, J. A.; Long, J. R., Selective nitrogen adsorption via backbonding in a metal–organic framework with exposed vanadium sites. *Nat. Mater.* **2020,** *19* (5), 517-521.

(20) Reed, D. A.; Keitz, B. K.; Oktawiec, J.; Mason, J. A.; Runčevski, T.; Xiao, D. J.; Darago, L. E.; Crocellà, V.; Bordiga, S.; Long, J. R., A spin transition mechanism for cooperative adsorption in metal–organic frameworks. *Nature* **2017,** *550* (7674), 96-100.

(21) Miner, E. M.; Park, S. S.; Dincă, M., High Li^+ and Mg^{2+} conductivity in a Cu-azolate metal–organic framework. *J. Am. Chem. Soc.* **2019,** *141* (10), 4422-4427.
(22) Wiers, B. M.; Foo, M.-L.; Balsara, N. P.; Long, J. R., A solid lithium electrolyte via addition of lithium isopropoxide to a metal–organic framework with open metal sites. *J. Am. Chem. Soc.* **2011,** *133* (37), 14522-14525.
(23) Xu, W.; Pei, X.; Diercks, C. S.; Lyu, H.; Ji, Z.; Yaghi, O. M., A metal–organic framework of organic vertices and polyoxometalate linkers as a solid-state electrolyte. *J. Am. Chem. Soc.* **2019,** *141* (44), 17522-17526.
(24) Fischer, S.; Roeser, J.; Lin, T. C.; DeBlock, R. H.; Lau, J.; Dunn, B. S.; Hoffmann, F.; Fröba, M.; Thomas, A.; Tolbert, S. H., A metal–organic framework with tetrahedral aluminate sites as a single-Ion Li+ solid electrolyte. *Angew. Chem. Int. Ed.* **2018,** *57* (51), 16683-16687.
(25) Du, Y.; Yang, H.; Whiteley, J. M.; Wan, S.; Jin, Y.; Lee, S. H.; Zhang, W., Ionic covalent organic frameworks with spiroborate linkage. *Angew. Chem. Int. Ed.* **2015,** *55* (5), 1737-1741.
(26) Ashraf, S.; Zuo, Y.; Li, S.; Liu, C.; Wang, H.; Feng, X.; Li, P.; Wang, B., Crystalline anionic germanate covalent organic framework for high CO2 selectivity and fast Li ion conduction. *Chem. Eur. J.* **2019,** *25* (59), 13479-13483.
(27) Hu, Y.; Dunlap, N.; Wan, S.; Lu, S.; Huang, S.; Sellinger, I.; Ortiz, M.; Jin, Y.; Lee, S.-h.; Zhang, W., Crystalline lithium imidazolate covalent organic frameworks with high Li-ion conductivity. *J. Am. Chem. Soc.* **2019,** *141* (18), 7518-7525.
(28) Xu, Q.; Tao, S.; Jiang, Q.; Jiang, D., Ion conduction in polyelectrolyte covalent organic frameworks. *J. Am. Chem. Soc.* **2018,** *140* (24), 7429-7432.
(29) Jeong, K.; Park, S.; Jung, G. Y.; Kim, S. H.; Lee, Y.-H.; Kwak, S. K.; Lee, S.-Y., Solvent-Free, Single Lithium-Ion Conducting Covalent Organic Frameworks. *J. Am. Chem. Soc.* **2019,** *141* (14), 5880-5885.
(30) Li, X.; Hou, Q.; Huang, W.; Xu, H.-S.; Wang, X.; Yu, W.; Li, R.; Zhang, K.; Wang, L.; Chen, Z.; Xie, K.; Loh, K. P., Solution-processable covalent organic framework electrolytes for all-solid-state Li–organic batteries. *ACS Energy Lett.* **2020,** *5* (11), 3498-3506.
(31) Choi, R. H.; So, J.; Kim, Y.; Lee, D.; Byon, H. R., Li^+ conduction of soft-base anion-immobilized covalent organic frameworks for all-solid-state lithium–metal batteries. *ACS Energy Lett.* **2024,** *9* (11), 5341-5348.

(32) Yan, Y.; Liu, Z.; Wan, T.; Li, W.; Qiu, Z.; Chi, C.; Huangfu, C.; Wang, G.; Qi, B.; Yan, Y.; Wei, T.; Fan, Z., Bioinspired design of Na-ion conduction channels in covalent organic frameworks for quasi-solid-state sodium batteries. *Nat. Commun.* **2023,** *14* (1), 3066.
(33) Zhao, G.; Xu, L.; Jiang, J.; Mei, Z.; An, Q.; Lv, P.; Yang, X.; Guo, H.; Sun, X., COFs-based electrolyte accelerates the Na^+ diffusion and restrains dendrite growth in quasi-solid-state organic batteries. *Nano Energy* **2022,** *92*.
(34) Jiang, G.; Zou, W.; Ou, Z.; Zhang, W.; Huo, J.; Qi, S.; Wang, L.; Du, L., Precise regulation of intra-nanopore charge microenvironment in covalent organic frameworks for efficient monovalent cation Transport. *Angew. Chem. Int. Ed.* **2025**, 10.1002/anie.202420333.
